# Supplementary material for: A modification of technology acceptance model for investigating driver-vehicle interaction systems usage
Source: PLoS One. 2025 Apr 22;20(4):e0322221. doi: 10.1371/journal.pone.0322221 (PMC12013904; doi:10.1371/journal.pone.0322221)
Supplement: S1 File — (DOCX) [file pone.0322221.s001.docx]

Blue&Me: <https://web.archive.org/web/20100511124618/http://www.fiat.com/cgi-bin/pbrand.dll/FIAT_COM/news/news.jsp?contentOID=1074078462>
