# Supplementary material for: A modification of technology acceptance model for investigating driver-vehicle interaction systems usage
Source: PLoS One. 2025 Apr 22;20(4):e0322221. doi: 10.1371/journal.pone.0322221 (PMC12013904; doi:10.1371/journal.pone.0322221)
Supplement: S1 Table — (DOCX) [file pone.0322221.s002.docx]

**Questionnaire content**

|  |  |
| --- | --- |
| Perceived usefulness | |
| PU1 | I have sufficient knowledge to use various functions in the vehicle. |
| PU2 | I can understand the marks presented by the interaction system accurately. |
| PU3 | I can understand the meaning of texts provided by the interaction system accurately. |
| PU4 | I can recognize the numbers provided by the interaction system immediately. |
|  |  |
| Perceived enjoyment | |
| PE1 | The environment and atmosphere inside the vehicle are comfortable to me. |
| PE2 | I am enjoying with the interaction functions in the system. |
|  |  |
| Satisfaction | |
| ST1 | I will not feel uncomfortable about the lights provided by the system when I drive at night. |
| ST2 | I am satisfied with the existing interaction systems. |
|  |  |
| Attitude | |
| AT1 | I would like to utilize different interaction functions in the system. |
| AT2 | I would like to renew its facilities, because I like to utilize the interaction system. |
|  |  |
| Interactive media | |
| IM1 | I think touchpad is useful for me to interact with the system. |
| IM2 | I think gesture is useful for me to interact with the system. |
| IM3 | I think voice is useful for me to interact with the system. |
|  |  |
| User interface | |
| UI1 | I would like to use simple UI. |
| UI2 | I would like to use complex UI. |
