# Supplementary material for: A modification of technology acceptance model for investigating driver-vehicle interaction systems usage
Source: PLoS One. 2025 Apr 22;20(4):e0322221. doi: 10.1371/journal.pone.0322221 (PMC12013904; doi:10.1371/journal.pone.0322221)
Supplement: Attachment — Submitted filename: Osswald2012.pdf [file pone.0322221.s003.pdf]

ResearchGate

See discussions, stats, and author profiles for this publication at: https://www.researchgate.net/publication/235798689

Predicting information technology usage in the car: towards a car technology acceptance model

Conference Paper · October 2012

DOI: 10.1145/2390256.2390264

CITATIONS
265

READS
15,100

5 authors, including:

Sebastian Osswald
BMW Group
49 PUBLICATIONS **508** CITATIONS
SEE PROFILE

Daniela Wurhofer
University of Salzburg
37 PUBLICATIONS **886** CITATIONS
SEE PROFILE

Sandra Trösterer
Virtual Vehicle
54 PUBLICATIONS **718** CITATIONS
SEE PROFILE

Elke Beck
36 PUBLICATIONS **716** CITATIONS
SEE PROFILE

All content following this page was uploaded by Sebastian Osswald on 17 January 2014.

The user has requested enhancement of the downloaded file.

Predicting Information Technology Usage in the Car: Towards a Car Technology Acceptance Model

Sebastian Osswald, Daniela Wurhofer, Sandra Trösterer,
Elke Beck and Manfred Tscheligi
CD Laboratory "Contextual Interfaces", ICT&S Center, University of Salzburg
Sigmund-Haffner Gasse-18, Salzburg, Austria
{sebastian.osswald, daniela.wurhofer, sandra.troesterer, elke.beck, manfred.tscheligi}@sbg.ac.at

ABSTRACT

This paper is aimed at studying information technology acceptance in an automotive context. Most models of technology acceptance focus on barriers of successful information technology implementation in organizations, while factors that take the contextual situation into account are neglected. We address this issue through deriving context-related determinants from an extensive literature review and a content analysis, and we further describe a technology acceptance modeling process to provide an explanation for drivers' acceptance of in-car technology. Based on our evaluation we take the determinants safety and anxiety into consideration, and propose a theoretical car technology acceptance model (CTAM) by incorporating these determinants into the Unified Theory of Acceptance and Use of Technology (UTAUT) model. Our modeling approach and proposed questionnaire support decision processes regarding in-vehicle information system implementation in the automotive industry as well as behavior prediction for research purposes.

Categories and Subject Descriptors

H.5.2 [**Information Interfaces and Presentation**]: HCI, Evaluation/methodology

Keywords

Automotive user interfaces, context, ctam, technology acceptance model

1. INTRODUCTION

The importance of automotive information systems is constantly rising as much effort is put into the development process of such systems. It is important to find out why individuals decide to use or not use an information system as the intention to use could have a major impact on traffic safety as well as on the decision to purchase a system/car. Being able to predict user acceptance would further be helpful in the development process to build appropriate systems and avoid issues that affect the acceptance of a system.

In the past, the technology acceptance model (TAM) [7] has received significant attention as it proposed to determine user acceptance towards a computer system. Recent research strives for applying the TAM or other models of technology acceptance in various contexts. In general, context can differ widely and possesses characteristics that need to be addressed when applying the TAM, as the original TAM explicitly focuses on a organizational context regarding desktop-based computer systems. However, the TAM is most often only partially altered and not reworked to aim for context specifics. Applying a technology acceptance model on the car context thus needs basic rework especially because state of the art models do not take contextual influences in the car like motion, environmental conditions or properties of novel information technologies into account. Motivating our work we want to point out three different car-related conditions that need to be addressed within a rework version of a technology acceptance model for the car context:

First, information technologies are closely connected to their context. They use contextual information like e.g. driving speed, position of the car or environmental conditions to provide driving-related information and support to the driver. As the characteristics of such *contextual interfaces* are neither addressed within the TAM nor in other technology acceptance models, we argue for reworking the TAM to make it applicable for the car context.

Second, the interaction with the information system is always subordinate during a driving situation. The driver often suffers from limited mental resources since the primary driving task competes with secondary/tertiary tasks. The space of interaction is limited as the driver is bound to his/her place and interaction is determined through e.g. reachability of interaction modalities or the capability of hand-eye coordination during system usage. Further, due to the motion of the car, the driving context comprises a constantly changing environment. Opposed to a comparably safe surrounding when using desktop-computer systems, the driver is constantly placed in a potentially risky situation. As the hazard for the physical condition of the driver persists as a contextual entity, we think that the perceived safety may influence the driver's technology acceptance of an information system in the automotive context.

Third, the driver gets assistance during the driving task through e.g. lane or speed keeping, range analysis or traffic information. The information needed for these services/ functions is dependent on sensors and the automated assis-

Permission to make digital or hard copies of all or part of this work for personal or classroom use is granted without fee provided that copies are not made or distributed for profit or commercial advantage and that copies bear this notice and the full citation on the first page. To copy otherwise, to republish, to post on servers or to redistribute to lists, requires prior specific permission and/or a fee.
AutomotiveUI'12, October 17-19, Portsmouth, NH, USA.
Copyright (c) 2012 ACM 978-1-4503-1751-1/12/10 ... $15.00

tance system that performs the desired activity. Especially within the upcoming era electric vehicles, one of the most addressed issues in this interdependency between assistance and hardware functionality is anxiety towards the energy range. People often do not rely on the information system that presents the information about the energy/fuel range of the vehicle. The system needs to be more reliable to allow for a seamless traveling experience and to reduce anxiety.

The paper proceeds as follows: First, technology acceptance is thoroughly reviewed to provide insights in the development process and the predictive character of acceptance models, which is followed by a description of the UTAUT model. Second, determinants that effect acceptance of in-car technologies are examined. We perform a literature review as well as a content analysis of qualitative results from a technology acceptance evaluation to derive and define the constructs "anxiety" and "perceived safety". Third, two novel determinants as well as the inherit determinants from the UTAUT model are described. They form the basis for a theoretical Car Technology Acceptance Model (CTAM) which is presented based on the UTAUT structure. Fourth, we describe the novel CTAM questionnaire for measuring the acceptance construct and report on the process and results from an initial questionnaire evaluation. Finally, the implications followed by future work are discussed.

2. RELATED WORK

The Technology Acceptance Model (TAM) [7] was developed to predict individual adoption and use of new information technologies and is "the single, most important factor in determining success or failure of information systems and technologies" [21]. It posits that individuals' behavioral intention to use an information technology is determined by two beliefs: (1) *perceived usefulness*, defined as the extent to which a person believes that using an information technology will enhance his or her performance and (2) *perceived ease of use*, defined as the degree to which a person believes that using an information technology will be free of effort. It further theorizes that the effect of external variables (e.g. design characteristics) on behavioral intention will be mediated by perceived usefulness and perceived ease of use.

Over the last two decades, there has been substantial empirical support in favor of the TAM (e.g. [1], [28],[27]). TAM consistently explains about 40% of the variance in individuals' intention to use an IT and the actual usage. Attempts to extend the TAM have generally taken one of three approaches: (1) introducing factors from related models, (2) introducing additional or alternative belief factors, and (3) examining antecedents and moderators of perceived usefulness and perceived ease of use [29]. Further work was put into developing and extending the TAM like with the TAM2 [26], the TAM3 [25], or the Unified Theories of Technology Acceptance Model (UTAUT) that unifies different models of technology acceptance [28]. Putting the focus away from desktop-based information systems, first work in adapting the technology acceptance model was carried out by Lowry et al. [13], who focused on the cultural differences in technology acceptance. Regarding the car context, initial work on applying the technology acceptance model in a preprototype stage like proposed in [6], was done by Meschtscherjakov et al. [15] and Osswald et al. [19]. In both studies, the authors adapted the original TAM in terms of slight alterations of the questionnaire wording and focus. Initial work regarding acceptance in the car context was performed by Van der Laan and colleagues [24] who proposed a simple procedure for the assessment of acceptance of advanced transport telematics. Also, Kantowitz and colleagues [12] evaluated driver acceptance of unreliable traffic information to estimate how reliable information has to be to trust and accept advices. Regarding safety and technology acceptance, Joshi and colleagues [11] focused on the perception of risk and control to understand acceptance of advanced driver interface systems.

2.1 Predictability of User Acceptance

Early prediction of usage behavior supports early evaluation of many different, competing designs of an information technology. Further, it helps avoiding pitfalls in the implementation process, which otherwise may have a lasting effect on the user perception which is hard to change. TAM accounts for around 40% of the variance in behavioral intention of use (BI) [7]. Through incorporating the social norm construct, the TAM2 reached a value of 52% in explaining BI [26]. The highest value nevertheless was reached by the UTAUT, a unified model of different overlapping constructs collected from various models that explain around 70% of the variance of BI [28]. This is why we think that combining factors that strive for explaining technology acceptance is a valuable approach. We further argue that the UTAUT model is a promising starting point for further investigations with regard to its high account values.

One of our goals for this research besides striving for explaining the behavioral intent, is to enable technology acceptance assessment early in the development process. Davis and Venkatesh [6] already discussed if the prospective user must have a direct hands-on experience with at least a working prototype. He showed that for estimating the *perceived ease of use* a direct behavioral experience through using a system is beneficial. But for predicting the *perceived usefulness* it is sufficient to provide information about a system's functionality. This enables us to evaluate technology user acceptance much earlier in a system development process than it has been the case traditionally. A preprototype acceptance evaluation has a "greater informational value than their postprototype counterparts" [6] because it is much more flexible, costs less and allows for early modification of system attributes. In our research towards car technology acceptance, we address this issue in the instrument development process, as the items measuring acceptance need to be designed for this purpose.

2.2 Unified Theory of Acceptance and Use of Technology (UTAUT)

The UTAUT [28] is one of the most recent instruments that aims for explaining technology acceptance. In this model, eight existing models of technology acceptance are synthesized. Venkatesh et al. [28] reviewed and integrated elements from: Technology Acceptance Model, Theory of Reasoned Action, Motivational Model, Theory of Planned Behavior, Model of PC Utilization, Innovation Diffusion Theory, and Social Cognition Theory.

Based on different categories and foci of the models, five constructs were combined to form the UTAUT model: performance expectancy, effort expectancy, social influence, facilitating conditions, and behavioral intention to use a system. Figure 1 shows the UTAUT research model without

the moderators gender, age, experience and voluntariness of use. We acknowledge this cross effect but dismiss these factors in our approach to reduce complexity. The model aims to explain user intention and usage behavior of an information technology. In a longitudinal evaluation, Venkatesh et al. [28] validated the model and described the factors mentioned above as key construct that affect usage intention and behavior. Unlike the other constructs who directly influenced behavioral intention, facilitating conditions was found to only have a significant effect on usage intention. Despite the criticism from Bagozzi [3], who stated that the extent of the model with its 41 independent variables for predicting intention and eight independent variables for predicting behavior is excessive, we decided to use the model as our starting point as the model is on the other hand the most complete one. The main reason for this decision was that the latest model, the TAM3 focuses on desktop-based computer systems even more than the previous models, and that the UTAUT represents a successfully integrated model that contains determinants from various resources.

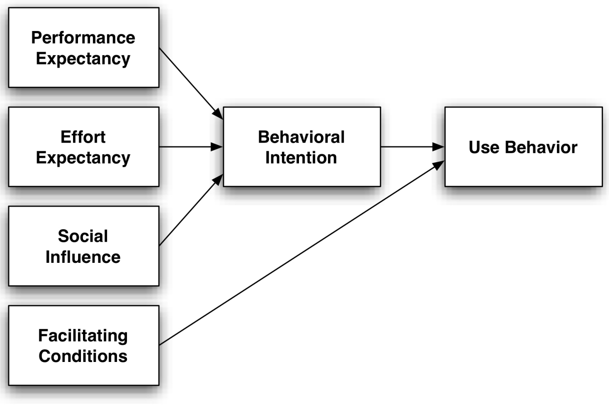

Figure 1: Unified Theory of Acceptance and Use of Technology (UTAUT) without the moderators: gender, age, experience and voluntariness of use

3. DETERMINANT EVALUATION

This section examines determinants that might affect technology acceptance of information systems in the car. First, perceived safety issues are identified by literature review and then examined with the help of a content analysis of responses of a technology acceptance questionnaire study (n=400). Second, anxiety issues that have appeared in the automotive research literature and earlier studies are discussed.

3.1 Perceived Safety While Driving

Perceived safety is crucial when driving a car. In a pilot study, Alm and Lindberg [2] showed that perceived accident risk and risk of violence are related to feelings of safety and worry associated with different transport modes (like e.g. car, taxi, city bus). Emotional factors and affective states can enhance or reduce traffic safety (e.g., [16], [8]). Nass et al. [17] showed that a match of user emotion and car voice emotion lead to fewer accidents and more attention on the road. Takayama & Nass [23] found that higher engagement with in-car systems compared to wireless systems leads to safer driving behavior.

Along with this first insight we got on perceived safety through related literature, we assumed that there has to be evidence about safety concerns in studies that dealt with new information technologies in the car. Based on our experience in investigating interactive technology in the car in a holistic way [14] through applying various methods to address safety concerns, we decided to examine a technology acceptance study (n=400) that we conducted earlier [19], to search for evidence of perceived safety. The study itself was designed to measure the technology acceptance of novel input modalities in the car with the help of a questionnaire. This questionnaire was based on the original TAM and was slightly altered in the wording to match the car context (e.g. the questionnaire was translated and the *job* was replaced through *driving*).

At the end of the questionnaire, the participants were provided a field for free comments of any kind. 81 subjects provided 114 different comments. To find out which are the most frequently expressed concerns, we decided to go for a content (topic) analysis of those comments. Due to efficiency reasons, the comments were split into three parts, and each of which was categorized by another HCI expert. After the categorization of topics was finished, the parts were merged again and the categories were checked for consistency to ensure that they all address the respective issue. In total, 13 categories could be identified: distraction, safety, usability, ease of use, learnability, design, familiarity, readability, (un)usefulness, comfort, novelty, robustness and simplicity. Finally, a recoding of all comments based on the defined categories was done by two experts. 29% of the coded comments addressed concerns about distraction from traffic, i.e. distraction that might be caused by the system. 27% of the comments were explicit concerns about safety, i.e. that the use of the system could be dangerous. 13% of the comments addressed usability issues, and 10% ease of use. All of the remaining categories appeared with an amount below 3% or 1% respectively. The results show that the topics distraction and safety are mentioned most often when it comes to the use of the system while driving. We assume that those categories can be summarized as *perceived safety* and appear to be prior regarding a driving situation. The high occurrence of safety issues (around one third of all comments addressed this issue; Cohen's Kappa = 0.792, i.e. good agreement) points out the importance of this factor in the context of driving and acceptance. Due to the apparent importance of safety with regard to technology acceptance in the car, we decided to add this factor to our proposed CTAM.

3.2 Anxiety in the Car Context

Öhman [18] points out the negative influence of anxiety on the attentional focus. Someone who is anxious may be less capable of handling a complex situation in which attention has to be divided over several objects or events. With regard to the automotive domain, anxiety seems to have an important influence on traffic safety. According to Spielberger et al. [22], trait anxiety is defined as the degree to which a person responds to situations with apprehension and uneasiness. One of the first taking this definition into account with regard to traffic anxiety were Ford and Alverson-Eiland [9]. They investigated the effects of anxiety on performance on a motorcycle course, concluding that

anxiety seems to be a moderately influential factor in predicting performance. . Banuls et al. [5] investigated the relation of accident involvement and anxiety in professional and novice drivers. Results showed that for novice drivers, anxiety responses to those situations that involve some kind of evaluation of driving may be connected to increased accident risk, whereas for professional drivers the more risky situations are anxiety responses to those situations that involve delays or impediments. Groeger [10] studied the relationships between drivers, their moods and their driving performance, considering anxiety as one specific mood (next to depression and hostility). The study showed that those who were more anxious performed worse in the driving task than less anxious participants. Anxiety addresses also privacy and data security issues (e.g. GPS monitoring, access to mobile phone). Related to this issue are the factors that govern reliability and complacency with safety systems like addressed in [20] within the context of human interaction with automation.

Anxiety as an acceptance determinant was already addressed within the UTAUT model [28] but was dropped as nonsignificant. The construct they used was based on factors borrowed from the Social Cognitive Theory (SCT) [4]. The SCT describes anxiety as a condition that evokes anxious or emotional reactions when it comes to showing a behavior. Regarding the strong connection we described between anxiety and driving, we state that anxiety appears to be a significant determinant of acceptance in the car context. We thus expect that anxiety to behave similar like the other determinants, which means that it has a direct effect on intention of use and is not absorbed by other determinants.

4. THE CTAM MODEL

In this section, we theorize that along with the two determinants *perceived safety* and *anxiety*, six further direct determinants from the UTAUT play a role within the car context: *performance expectancy, effort expectancy, social influence, facilitating conditions, self-efficacy, attitude towards using technology* and *self efficacy.* Figure 2 shows an overview about our proposed CTAM model. The elements used within this figure reflect the different determinant constructs that are briefly described below. The grey items represent the remaining determinants from the original UTAUT model. In the following, we give the description and definition of all determinant constructs. Further, we link the derived determinants to the original constructs and models.

4.1 Performance Expectancy

Performance expectancy is defined as the degree to which a driver believes that using the system will help him or her to reach goals in driving performance. We understand driving performance as one's individual goal completion during a car trip. This either can be a global goal like to reach a destination safely, spend less fuel or energy, or a task-related goal like to use an information system in a way that it allows for an efficient and effective task completion. According to [28], the performance expectancy construct is the strongest predictor of intention. The constructs used to build performance expectancy are: *perceived usefulness* from the TAM, *relative advantage* from the Innovation Diffusion Theory and *outcome expectations* from the SCT.

4.2 Effort Expectancy

Effort expectancy is defined as the degree of ease associated with the use of a system. Especially in the car it is of intrinsic importance to quickly be able to perform a task while driving without lasting periods of trial and error. As secondary tasks affect the driving performance, it is mandatory that a system is easy to use and the system input and output is clearly to understand. According to Davis [7], effort-oriented constructs are expected to be more salient in the early stages of a new behavior. Thus, evaluating effort expectancy allows for a valuable insight into the perceived difficulty in using an system. Two constructs from existing models capture the concept of effort expectancy: *perceived ease of use* from the TAM and *ease of use* from the Innovation Diffusion Theory.

4.3 Attitude Towards Using Technology

Attitude towards using technology is defined as an individual's overall affective reaction upon using a system. This determinant aims at reflecting the beliefs of the user regarding system usage and its effects. It addresses attributes of in-car information systems that lie beyond pure functionality and tries to determine the attitude towards using the system through affective items like fun and likeability. These constructs did not significantly affected the behavioral intent in the UTAUT evaluation, but we reintroduce this construct since we cannot estimate the effect in the car context in advance. The constructs and origins that influences attitude towards using technology are: *attitude towards behavior* from the Theory of Reasoned Action, *affect towards use* from the Model of PC Utilization and *affect* from the SCT.

4.4 Social Influence

Social influence is defined as the degree to which an individual believes that other people whose opinion is important to himself/herself think the same way about using a new system. The underlying constructs are: *subjective norm* from the TRA and *social factors* from the Model of PC Utilization. Especially the *social factor* items were adapted as they strongly focused on computer systems in organizations e.g.: "The senior management of this business has been helpful in the use of the system." The translated items can be found in table 1. Venkatesh et al. [28] further point out that "the role of social influence in technology acceptance decisions is complex and subject to a wide range of contingent influences." Social influence has an impact on the driver's individual behavior through three mechanisms: compliance, internalization, and identification. Since the car itself as well as integrated technology are often regarded as a status symbol, we think that the representative character and the properties and features of information technology can foster identification and highlight the connection between acceptance and the social environment.

4.5 Facilitating Conditions

Facilitating conditions are defined as the degree to which an individual believes that a technical infrastructure (like a help menu) or somebody (like a front seat passenger) exist to support in the use of the system. This comprises the perception of internal and external constraints on behavior and technology facilitating conditions as well as objective factors that driver agree make an task easy to perform. In the car this could be interpreted as the availability of a learning tool,

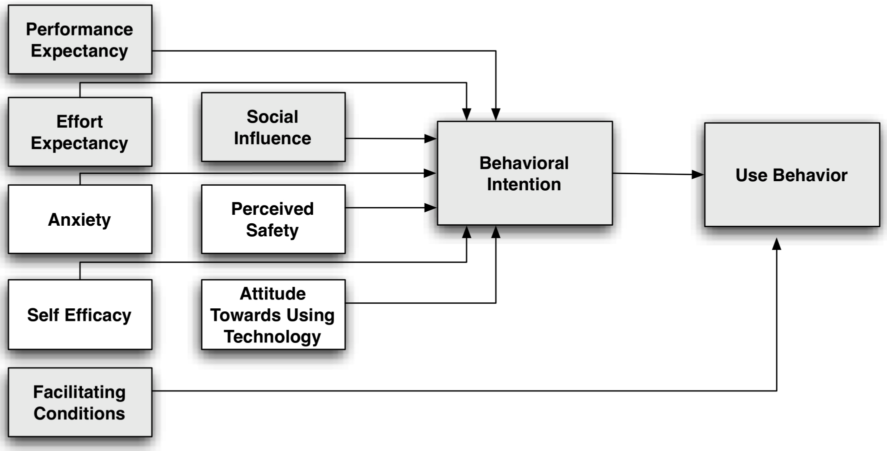

Figure 2: Car Technology Acceptance Research Model (The grey determinants are inherited from the UTAUT)

a help menu to search for hints or a manual that supports to understand or perform a certain task. Additionally, support can be carried out by passengers in the car e.g. through preselecting a functionality for the driver to ease e.g. navigational input.The constructs used are *perceived behavioral control* from the Theory of Planned Behavior and *facilitating conditions* from the Model of PC Utilization.

4.6 Self-Efficacy

The determinant self-efficacy is defined as a person's belief in his/her ability and competence to use a technology (e.g. a radio) to accomplish a particular task. It can play a major role in how one approaches a goal or a task as it is developed from external experiences and self-perception. In the car context, people with high self-efficacy - those who believe they can perform well - are more likely to view a difficult task as something to be mastered rather that something to be avoided. Alining results from this construct with results from the perceived safety construct, we believe that this will gives us an interesting insight into how an information system-related task is judged based on users' personality characteristics. We apply self-efficacy because it is the core determinant of the SCT and was proven valid in several studies, yet it was determined as non-significant within the UTAUT. The construct used is *self efficacy*, from the SCT.

4.7 Anxiety

Based on our findings we define anxiety in the car context as the degree to which a person responds to a situation with apprehension, uneasiness or feelings of arousal. The factors *Anxiety* and *Behavioral Anxiety* differ with regards to their origin, as *Anxiety* was derived from a computer anxiety construct described within the SCT and was used earlier in the UTAUT validation. *Behavioral Anxiety* otherwise reflects anxiety in a more general understanding towards the vehicle or system behavior which addresses e.g. the fear to lose control of the car. Anxiety was found not significant within the UTAUT model, but was found to be a significant determinant of intention in the SCT. The items used in the UTAUT strongly focused on computer-anxiety, which explicitly addresses the fear to use a computer because of a general technology inexperience. We thus assume a significant difference regarding the overall technology experience nowadays and within the car, and especially regarding the focus on computer anxiety.

4.8 Perceived Safety

We define perceived safety as the degree to which an individual believes that using a system will affect his or her well-being. We named the construct *perceived safety* considering the self-reflective character of perceiving a situation hazardous. Within the car, this also comprises the judgment of one's own driving skills and safety feeling in relation to other drivers. The impact of perceived safety is assumed as critical in the process of predicting the behavioral intention to use, as the user will estimate the potential effect of safety-related consequences through using an information technology while driving.

5. CAPTURING CAR TECHNOLOGY ACCEPTANCE

We have taken two steps to validate the model: The first step was to develop the instrument items to measure the determinant constructs listed in table 1. The second step was to use the instrument to study technology acceptance in a user study in order to instantiate the model, i.e. determining if the factor constructs that potentially affect user acceptance internally correlate.

5.1 Instruments

For capturing car technology acceptance, we developed the CTAM questionnaire items shown in table 1. The respective origin constructs are reported in the first column. An issue that appeared during the questionnaire construction was the temporal mismatch between the different measures in the model. The same problem could already be noticed in the original UTAUT model wherein sentences with predictive character using "would" and "will" constellations are mixed with declarative sentences. This can be traced to the different roots of technology acceptance models and is one of the reasons why we tried to unify the wording. We

Table 1: Items Used in Estimating CTAM

Item overview	
**Performance expectancy**	
U	The system would be useful while driving.
RA1	Using the system enables me to accomplish my goals more quickly.
RA2	Using the system increases my driving performance.
OE	If I would use the system I will reach my destination safely.
**Effort expectancy**	
EOU1	My interaction with the system would be clear and understandable.
EOU2	It would be easy for me to become skillful at using the system.
EOU3	I find the system easy to use.
EU	Learning how to operate the system is easy for me.
**Attitude towards using technology**	
A	Using the system is a good idea.
AF1	The system makes driving more interesting.
AF2	Interacting with the system would be fun.
Affect	I would like interacting with the system.
**Social influence**	
SN1	I would be proud to show the system to people who are close to me.
SN2	People whose opinions are important to me would like the system too.
SF1	My passenger(s) would be helpful when using the system.
SF2	In general, people who I like would encourage me to use the system.
**Facilitating conditions**	
PBC1	While using the system I can maintain an safely driving behavior.
PBC2	I have the knowledge necessary to use the system.
PBC3	The system is compatible with other systems I use.
FC	There would be somebody I can ask for assistance with system difficulties.
**Self efficacy**	I could complete a task or activity using the system..
SE1	.. if there was no one around to tell me what to do.
SE2	.. if I could call someone for help if I got stuck.
SE3	.. if I had a lot of time.
SE4	.. if I had just the built-in help facility for assistance.
**Anxiety**	
ANX1	I have concerns about using the system.
ANX2	I think I could have an accident because of using the system.
ANX3	The system is somewhat frightening to me.
BA1	I fear that I do not reach my destination because of the system.
BA2	I am afraid that I do not understand the system.
BA3	I am confident that the system does not affect my driving.
**Behavioral intention to use the system**	
BI1	Assuming I had access to the system, I intend to use it.
BI2	Given that I had access to the system, I predict that I would use.
BI3	If the system is available I plan to use the system in the next months.
**Perceived Safety**	
PS1	I believe that using the system is dangerous.
PS2	Using the system requires increased attention.
PS3	The system distracts me from driving.
PS4	I feel save while using the system.
PS5	Using the system decreases the accident risk.
PS6	I can use the system without looking at it.

U= perceived usefulness; RA= relative advantage; OE= outcome expectations; EOU= perceived ease of use; EU= ease of use; A= attitude; AF= affect towards use; Affect= affect; SN= subjective norm; SF= social factors; PBC= perceived behavioral control; FC= facilitating conditions; SE= self efficacy; ANX= anxiety; BA=behavioral anxiety ; BI= behavioral intention to use technology; PS = perceived safety .

noticed in an early pretest of the instruments that it was difficult and hence impossible to determine one's approval or disapproval of acceptance to some of the original questions. This was caused by declarative sentences which could not be used for estimating the acceptance of a future situation. We modified the questions in a way that they are more consistent among themselves and allow to comprehend the effect of a usage scenario.

5.2 Method

For a first validation of the developed CTAM questionnaire, we used it in a study that aimed at evaluating a preliminary prototype of an in-car text input system. For that purpose we set up a fixed-based driving simulator at a public event where people were invited to actively get in touch with university research activities. Formal training with the prototype was conducted with voluntary participants during the event. The text-input system was controlled by six buttons on the back of the steering wheel and the visual output was displayed in a head-up display. The potential users made themselves comfortable with the functions and wrote short messages during a driving situation. Afterwards we invited the participants to fill in either a paper-based version of the CTAM questionnaire or an online version. Additionally, we asked for demographical data like gender, age and driving license.

5.3 Results

In total, 21 subjects (16 male, 5 female) filled in the questionnaire. The mean age of the subjects was 33 (SD= 4,56), the youngest person was 20, the eldest 47 years. Except for one subject, all participants possessed a driving license. In order to determine the internal consistency of the scales, the reliability coefficient Cronbach's α was computed for each scale. Table 2 gives an overview of the reliability coefficients. We found good internal consistency for nearly all scales (Cronbach's α higher than 0.8), except Facilitating Conditions (Cronbach's $\alpha = 0.671$) and Anxiety (Cronbach's $\alpha = 0.782$).

A more detailed analysis of the Anxiety items showed that dropping the item BA2 "I am afraid that I do not understand the system" increases the reliability (Cronbach's $\alpha = 0.819$). For the items of the Facilitating Conditions scale we could not find an improvement of the reliability coefficient when dropping a specific item.

With regard to the new scale perceived safety, we found a Cronbach's α of 0.858, indicating a good internal consistency of the scale. We are aware that for a proper validation of the questionnaire the number of subjects needs to be higher. We also see that some of the constructs might not be independent from others However, we think that these initial results give a first hint that the developed instrument does not comprise severe weaknesses.

Table 2: Internal Reliabilities of the Instruments

Scale	*Cronbach's* α
Performance expectancy	0.876
Effort expectancy	0.921
Attitude towards using technology	0.900
Social influence	0.825
Facilitating conditions	0.671
Self efficacy	0.874
Anxiety	0.782
Behavioral intention to use the system	0.913
Perceived Safety	0.858

6. DISCUSSION

We developed a rich understanding of determinants of information technology adoption and use which aim at supporting decision making processes in the development and implementation phase. Determining technology acceptance can help to identify utilization weaknesses and support to lower barriers of adoption. In our study we ascertained a good consistency of the proposed scales and the results suggest that the participants are aware of the safety- and anxiety-related influences during information technology usage while driving. We cannot exclude that there will be further determinants that need to be considered within the car context, but based on our research we believe that we addressed the major determinants. Regarding the continuous development process of technology acceptance models it further can be said that the process itself is iterative and needs for further studies to enhance the overall generalizability of the CTAM model. We further have to critically address the state of the prototype in further evaluations and address effects and differences between a pre-prototype and a prototype state.

The low values for facilitating conditions further suggest that items of other constructs are substitutive, why we need to address this issue in future work to gain statistically significant results. We do need to look across the determinants to estimate how indented they are, why a questionnaire study aiming at a larger target group and other information technologies needs to be carried out to allow for a partial least squares analysis. This method would be used for the estimation of the path model that involves the latent constructs that are indirectly measured by the multiple indicators to estimate the weight and the loadings of the scales. This likewise describes the limitation of our approach: We gained a valuable insight into car technology acceptance and developed a consistent instrument for measuring acceptance but need for further validation to estimate boundary conditions.

7. CONCLUSION AND FUTURE WORK

Information technology in the car becomes increasingly complex. One of the major challenges is to design feasible solutions that support the driver but do not affect the driving performance. Addressing this challenge, this paper describes and defines car context-related acceptance determinants and proposes the Car Technology Acceptance Model (CTAM). It extends the UTAUT model's range to explain and predict technology acceptance of drivers regarding information technology in the moving context of a car. We introduced anxiety and perceived safety as relevant additional determinants and conducted an initial study to test the reliability of the single scales (constructs) with the aim to refine the proposed model in future work.

Future research needs to assess how the determinants perform over time as the expression of acceptance clearly vary regarding the familiarity with a certain information technology. It is assumed that acceptance can be seen as an

user experience factor and thus underlies similar influence regarding the time dimension (e.g. stimulation, identification). Further, the key moderators [28]: age, gender, experience and voluntariness of use, need to be addressed as they might influence on the severity of technology acceptance.

Acknowledgements

The financial support by the Federal Ministry of Economy, Family and Youth, the National Foundation for Research, Technology and Development and AUDIO MOBIL Elektronik GmbH is gratefully acknowledged (Christian Doppler Laboratory for "Contextual Interfaces").

8. REFERENCES

[1] D. Adams, R. Nelson, and P. Todd. Perceived usefulness, ease of use, and usage of information technology: a replication. *MIS quarterly*, pages 227–247, 1992.

[2] C. Alm and E. Lindberg. *Perceived Risk, Feelings of Safety and Worry Associated with Different Travel Modes: Pilot Study.* KFB, 2000.

[3] R. Bagozzi. The legacy of the technology acceptance model and a proposal for a paradigm shift. *Journal of the Association for Information Systems*, 8(4):244–254, 2007.

[4] A. Bandura. *Social Foundations of Thought and Action: A Social Cognitive Theory, Prentice.* Hall, Englewood Cliffs, NJ, 1986.

[5] R. Bañuls Egeda, E. Carbonell Vaya, M. Casanoves, and M. Chisvert. Different emotional responses in novice and professional drivers. In T. Rothengatter and E. C. Vaya, editors, *Traffic and Transport Psychology: Theory and Application.* Pergamon, 1996.

[6] F. Davis and V. Venkatesh. Toward preprototype user acceptance testing of new information systems: implications for software project management. *Engineering Management, IEEE*, 51(1):31 – 46, 2004.

[7] F. D. Davis. Perceived usefulness, perceived ease of use, and user acceptance of information technology. *MIS Quarterly*, 13(3):318+, 1989.

[8] F. Eyben, M. Wöllmer, T. Poitschke, B. Schuller, C. Blaschke, B. Färber, and N. Nguyen-Thien. Emotion on the road: necessity, acceptance, and feasibility of affective computing in the car. *Adv. in Hum.-Comp. Int.*, 2010:5:1–5:17, Jan. 2010.

[9] F. Ford and L. Alverson-Eiland. The relationship between anxiety and task performance and skill acquisition in the motorcycle safety foundation's motorcycle rider course. In *IMC'91*, pages 363–379, Bochum, 1991.

[10] J. Groeger. Mood and driving: Is there an effect of affect? In *Traffic and Transport Psychology: Theory and Application.* Pergamon, 1997.

[11] S. Joshi, T. Bellet, V. Bodard, and A. Amditis. Perceptions of risk and control: Understanding acceptance of advanced driver assistance systems. *INTERACT'09*, pages 524–527, 2009.

[12] B. Kantowitz, R. Hanowski, and S. Kantowitz. Driver acceptance of unreliable traffic information in familiar and unfamiliar settings. *Human Factors: The Journal of the Human Factors and Ergonomics Society*, 39(2):164–176, 1997.

[13] G. Lowry. Translation and validation of the technology acceptance model and instrument for use in the arab world. In *ACIS 2004*, 2004.

[14] A. Meschtscherjakov, D. Wilfinger, N. Gridling, K. Neureiter, and M. Tscheligi. Capture the car! qualitative in-situ methods to grasp the automotive context. In *AUI'11.* ACM, 2011.

[15] A. Meschtscherjakov, D. Wilfinger, T. Scherndl, and M. Tscheligi. Acceptance of future persuasive in-car interfaces towards a more economic driving behaviour. In *AUI'09*, pages 81–88. ACM, 2009.

[16] J. Mesken. *Measuring Emotions in Traffic.* SWOV Institute for Road Safety Research, 2002.

[17] C. Nass, I.-M. Jonsson, H. Harris, B. Reaves, J. Endo, S. Brave, and L. Takayama. Improving automotive safety by pairing driver emotion and car voice emotion. In *CHI EA '05*, pages 1973–1976, 2005.

[18] A. Öhman. Fear and anxiety: Evolutionary, cognitive and clinical perspectives. In M. Lewis and J. Haviland-Jones, editors, *Handbook of Emotions.* Guilford Press, 2000.

[19] S. Osswald, A. Meschtscherjakov, D. Wilfinger, and M. Tscheligi. Interacting with the steering wheel: Potential reductions in driver distraction. *AMI'11*, pages 11–20, 2011.

[20] R. Parasuraman, T. Sheridan, and C. Wickens. A model for types and levels of human interaction with automation. *Systems, Man and Cybernetics: Systems and Humans*, 30(3):286–297, 2000.

[21] C. Sauer. *Why information systems fail: a case study approach.* Alfred Waller Ltd., Oxfordshire, UK, 1993.

[22] C. Spielberger, R. Gorsuch, and L. Edward. *STAI manual for the state-trait anxiety inventory ("Self-evaluation questionnaire").* Consulting Psychologists Press, 1970.

[23] L. Takayama and C. Nass. Driver safety and information from afar: An experimental driving simulator study of wireless vs. in-car information services. *Int. J. HCS*, 66(3):173–184, Mar. 2008.

[24] J. Van Der Laan, A. Heino, and D. De Waard. A simple procedure for the assessment of acceptance of advanced transport telematics. *Transportation Research: Emerging Technologies*, 5(1):1–10, 1997.

[25] V. Venkatesh and H. Bala. Technology acceptance model 3 and a research agenda on interventions. *Decision Sciences*, 39(2):273–315, 2008.

[26] V. Venkatesh and F. Davis. A theoretical extension of the technology acceptance model: Four longitudinal field studies. *Management science*, pages 186–204, 2000.

[27] V. Venkatesh, F. Davis, and M. Morris. Dead or alive? the development, trajectory and future of technology adoption research. *Journal of the Association for Information Systems*, 8(4):267–286, 2007.

[28] V. Venkatesh, M. Morris, G. Davis, and F. Davis. User acceptance of information technology: Toward a unified view. *MIS quarterly*, pages 425–478, 2003.

[29] B. Wixom and P. Todd. A theoretical integration of user satisfaction and technology acceptance. *Information systems research*, 16(1):85–102, 2005.

View publication stats
