# Supplementary material for: A modification of technology acceptance model for investigating driver-vehicle interaction systems usage
Source: PLoS One. 2025 Apr 22;20(4):e0322221. doi: 10.1371/journal.pone.0322221 (PMC12013904; doi:10.1371/journal.pone.0322221)
Supplement: Attachment — Submitted filename: 1-s2.0-S0040162520311458-main.pdf [file pone.0322221.s004.pdf]

Technological Forecasting & Social Change 161 (2020) 120319

Contents lists available at ScienceDirect

Technological Forecasting & Social Change

journal homepage: www.elsevier.com/locate/techfore

What drives the acceptance of autonomous driving? An investigation of acceptance factors from an end-user's perspective

Ilja Nastjuk[a,*], Bernd Herrenkind[a], Mauricio Marrone[b], Alfred Benedikt Brendel[a], Lutz M. Kolbe[a]

[a] Georg-August-Universität Göttingen, Chair of Information Management, Platz der Göttinger Sieben 5, 37073 Göttingen, Germany
[b] Macquarie University – Faculty of Business and Economics, Balaclava Road, North Ryde NSW, 2109 Sydney, Australia

A R T I C L E I N F O

Keywords:
Autonomous driving
User acceptance
Technology acceptance model

A B S T R A C T

Autonomous driving is believed to provide numerous benefits for individuals and society, including increased road safety, reduced traffic congestion, and an improved ecological footprint. However, many barriers still hinder the widespread acceptance of autonomous vehicles. Research has proposed governmental policy strategies to accelerate the diffusion of autonomous driving, but less is known about end-user perceptions of this innovative technology. First, we employ a qualitative research design to identify the elements attributed to individual acceptance of autonomous driving. Furthermore, we organize a research model based on the technology acceptance model, validated with an online survey of 316 participants. The findings reveal how social influence, system characteristics, and individual factors determine individual acceptance of autonomous driving. The research helps to strengthen the existing body of knowledge by highlighting individual perceptions, with implications for practitioners.

1. Introduction

Full self-driving automation is the next big mobility-related disruptive innovation, and is expected to enter the mass market in the coming decades (Nieuwenhuijsen et al., 2018). Several automotive and technology companies have already demonstrated the practicability of autonomous driving through well-engineered prototypes and pilots. For example, in 2018, Waymo's test fleet logged over five million miles driving in autonomous mode on public roads (Merfeld et al., 2019a; Waymo, 2018).

Integrating intelligent automation technology into vehicles has several benefits. On an individual level, fully automated driving can lower commuter-related stress because the time can be used as desired when on the road, for example, for eating, sleeping, watching TV, or working (Eugensson et al., 2013; Shabanpour et al., 2017; Zmud et al., 2017). Another potential individual benefit is related to improved access to mobility, especially for elderly and physically impaired people, by ensuring the secure participation of these populations in traffic. In addition, autonomous driving might reduce chauffeuring burdens for family members and, in some cases, increase access to education and employment opportunities (Anderson et al., 2016; Hohenberger et al., 2017; König and Neumayr, 2017).

On a societal level, fully automated vehicles have the potential to enhance vehicle safety by practically eliminating human-related elements and errors that affect human drivers' performance, such as aging, disease, stress, fatigue, inexperience, or even drug abuse (Beiker, 2012; Shanker et al., 2013). Advocates have estimated that driverless cars may reduce traffic fatalities by up to 90% by mid-century (Bertoncello and Wee, 2015). In addition, research emphasizes an ecological potential for autonomous driving, stemming from reductions in carbon dioxide emissions and fuel consumption by optimizing traffic flow (Brown et al., 2014). Autonomous driving is also expected to optimize land usage, because the vehicles are able to drop off passengers in dense metropolitan areas before driving themselves to nearby satellite parking areas (Anderson et al., 2016). Furthermore, autonomous vehicles might help to counteract urbanization because commuting time becomes less important when it can be used for other activities (Marletto, 2019).

However, research has also outlined several individual and social concerns with regard to autonomous vehicle deployment. These include the high costs induced by maintenance expenses, road user fees, and software and hardware components (e.g., Casley et al., 2013; Fishman and Davies, 2016; Varghese and Boone, 2015); a rise in fuel consumption and carbon dioxide emissions resulting from increased travel demand (Brown et al., 2014); legal and ethical issues relating to the protection of users and pedestrians (Bonnefon et al., 2016;

* Corresponding author.
E-mail addresses: ilja.nastjuk@wiwi.uni-goettingen.de (I. Nastjuk), bernd.herrenkind@mhp.com (B. Herrenkind), mauricio.marrone@mq.edu.au (M. Marrone), abrende1@uni-goettingen.de (A.B. Brendel), lkolbe@uni-goettingen.de (L.M. Kolbe).

https://doi.org/10.1016/j.techfore.2020.120319
Received 10 February 2019; Received in revised form 9 July 2020; Accepted 13 September 2020
Available online 24 September 2020
0040-1625/ © 2020 The Author(s). Published by Elsevier Inc. This is an open access article under the CC BY-NC-ND license
(http://creativecommons.org/licenses/by-nc-nd/4.0/).

Hevelke and Nida-Ruemelin, 2015); privacy concerns and the potential for malicious hacking (Bansal et al., 2016; Douma and Palodichuk, 2012; Hulse et al., 2018); safety concerns in mixed traffic situations (Thomopoulos and Givoni, 2015); and loss of jobs for alternative transportation providers (Taeihagh and Lim, 2019).

However, it is argued that the biggest barrier to widespread adoption of autonomous driving is psychological, not technical, in nature (Shariff et al., 2017; Xu et al., 2018). Thus, the user's acceptance of autonomous driving is essential for autonomous driving to become a realistic part of future transportation (Panagiotopoulos and Dimitrakopoulos, 2018; Xu et al., 2018). Acceptance can be defined as the individual's willingness to use a technology for the tasks it is designed to support (Dillon and Morris, 1996). Since there are considerable reservations about autonomous driving, and this innovative technology has yet to be introduced to the market on a mass scale (Haboucha et al., 2017; König and Neumayr, 2017), understanding the individual's intentions and attitudes toward autonomous driving is crucial if this technological innovation is to be successful (Choi and Ji, 2015; Nordhoff et al., 2016; Xu et al., 2018). We, therefore, argue that a comprehensive understanding of the elements that influence user acceptance has the potential to inherently advance this technology's success in the market.

Although a number of studies in the broader literature have recognized the need for research into the factors that determine the acceptance of autonomous driving (e.g., Anania et al., 2018; Buckley et al., 2018a; Panagiotopoulos and Dimitrakopoulos, 2018; Wu et al., 2019; Xu et al., 2018), knowledge about the public acceptance of autonomous driving is still limited and more research is required to understand the psychological determinants of user acceptance (Buckley et al., 2018a; Xu et al., 2018). The particular research gaps that we address in this article are three-fold. First, a literature review on autonomous driving and acceptance by Gkartzonikas and Gkritza (2019) reveals that much research has focused on specific acceptance factors such as trust, driving pleasure, or safety to understand the acceptance of autonomous driving. The authors state that “most studies have examined the behavioral characteristics, perceptions, and attitudes related to AVs [autonomous vehicles] using descriptive analysis or some sort of econometric analysis” (p. 335). They call for more research on the interrelation between these factors and behavioral outcomes. To address this research gap, additional studies are needed to understand the interrelation between the various acceptance factors and behavioral intentions with the aid of established behavioral models. Second, Lee et al. (2019) and Xu et al. (2018) conclude that existing studies present incongruent or conflicting findings on the determinants influencing willingness to use autonomous driving, thus calling for more research in this field of study. In particular, there “is a need to study several psychological factors together to extend the understanding of user's perception of autonomous vehicles” (Lee et al., 2019, p. 412). Third, another limitation of previous studies is outlined by Merfeld et al. (2019a) and Buckley et al. (2018b), emphasizing that most insights in this research field have been assessed quantitively (see also Becker and Axhausen, 2017; Gkartzonikas and Gkritza, 2019). Thus, relevant factors that shape the acceptance of autonomous driving from an end-user perspective might have been overlooked (Venkatesh et al., 2013; Wu, 2012). Consequently, further studies are required to reveal individual perceptions of autonomous driving to gain a deeper understanding of related acceptance factors (Buckley et al., 2018b; Merfeld et al., 2019a; Nordhoff et al., 2019). Section 2 outlines the current state of the literature and elaborates in more detail on the research gaps specific to autonomous driving acceptance.

Thus, the main objective of this research is to generate an understanding of the elements potentially influencing individual acceptance, that is, the user's willingness to accept and adopt driverless vehicles (Nordhoff et al., 2016), employing fully autonomous vehicles with autonomy level 5 (SAE level 5 “Full Driving Automation” (SAE International, 2018)). These vehicles are designed to drive completely autonomously; in other words, the driver is not expected to control the vehicle during the trip. Level 5 offers significant benefits (e.g., Brell et al., 2019; König and Neumayr, 2017; Liljamo et al., 2018; Shabanpour et al., 2017; Skeete, 2018) and is also the center of attention for automotive manufacturers (Reese, 2016; Schweitzer et al., 2019). Although other studies focus on specific use cases of autonomous vehicles like public transportation, ownership, or sharing (Acheampong and Cugurullo, 2019; Kaur and Rampersad, 2018; Madigan et al., 2017), we aim to explore the acceptance of autonomous vehicles more generally to first fully understand the basic factors influencing acceptance from a user-centric perspective. With this approach, we identify relevant constructs that might influence the acceptance of autonomous vehicles but have not yet been addressed in prior research. Consequently, we present the following research question:

> What drives the acceptance of autonomous driving from an end-user perspective?

To address the research question and to overcome the above-mentioned limitations, we apply an exploratory sequential mixed methods design (Creswell et al., 2003) in which we first collect qualitative data to capture relevant acceptance criteria from an end-user point and then analyze and build on the results through quantitative data collection. By doing so, we contribute to existing research by disclosing factors that drive the acceptance of a technology from an end-user's point of view (Venkatesh et al., 2013; Wu, 2012). In addition, we contribute to existing research by identifying motivational patterns associated with the use of autonomous driving (Merfeld et al., 2019a), while at the same time revealing the relationships between these factors (Lee et al., 2019). Such an approach, to the best of our knowledge, has not been applied by prior research into autonomous driving acceptance and, thus, presents a valuable alternative to studies that derive acceptance factors that are based on literature (e.g., Hegner et al., 2019; Lee et al., 2019; Panagiotopoulos and Dimitrakopoulos, 2018).

The remainder of this paper is structured as follows. Section 2 discusses the current research on autonomous vehicle acceptance. Using an exploratory approach with a qualitative research design, we conducted semi-structured interviews to reveal relevant autonomous driving acceptance criteria from an end-user point of view, and details of this process are presented in Section 3. Based upon the well-established technology acceptance model (TAM) proposed by Davis et al. (1989), we then present a research model for approaching the stated research objective, as well as the hypothesized relationships formed from an online-based survey. Finally, we discuss the findings with a focus on implications for research and practice.

2. Literature review

Predicting the acceptance of autonomous driving is a fairly new topic (Bansal and Kockelman, 2017; Haboucha et al., 2017). However, several studies have been carried out to forecast its future. In order to identify the span of the research on the acceptance of autonomous driving, we conducted a literature review. The results of the 37 articles analyzed are documented in Appendix A. Throughout the review, the content of each article was examined to determine the study's objective, its research context and focus, methods used, theoretical framework applied, investigated constructs, derivation of extensions, and main findings. Appendix A, column (1) briefly describes the target of the study, while column (2) briefly describes some specifics of the research. The method column provides insights on the techniques and procedures used, and the theoretical framework column lists the theoretical foundations applied. The investigated constructs column summarizes the respective constructs used within the research while the derivation of extensions column describes how these constructs were obtained. The main findings column presents the results of each study. In the

following, the key content of each considered study is outlined briefly. We conclude by outlining the main research gaps and the targeted contribution of our study.

In an online survey of French drivers, Payre et al. (2014) reveal that the intention to use fully automated driving is determined by attitudes, contextual acceptability, sensation-seeking, and an interest in impaired driving. In an investigation of the influence of user experience with and acceptance of different automation levels in vehicles (in terms of fun, trust, perceived control, or attitudes), Roedel et al. (2014) show that both user experience and acceptance decrease continuously in relation to higher levels of automation. These results are in line with those of Cho et al. (2017), who also investigate the relationship between user experience and automation level. Based on the unified theory of acceptance and use of technology (UTAUT) model and the Pleasure-Arousal-Dominance (PAD) framework, Nordhoff et al. (2016) developed a conceptual model to understand the acceptance of autonomous driving; however, an empirical investigation of the proposed framework is still pending.

Using hypothetical scenarios, Anania et al. (2018) examine how the exposure of positive and negative information about driverless cars affects consumers' willingness to use such vehicles. Hudson et al. (2019) reveal that attitudes toward autonomous vehicles are determined by robot technophobia and self-interest. In addition, they emphasize that acceptance of autonomous driving differs between countries and depends on sociodemographic characteristics (e.g., gender, age, or level of education). In this regard, Nishihori et al. (2020) found that population density, gender, usage intentions, and the degree of commitment to autonomous vehicles affect the social acceptance of autonomous vehicles in Japan. Yuen et al. (2020) propose and validate a theoretical model to understand user acceptance of autonomous driving in Korea. The researchers identify that the impact of innovation diffusion attributes (e.g., trialability, relative advantage, complexity, compatibility, observability, and trust) on acceptance is mediated by the perceived value of autonomous driving. Manfreda et al. (2019) adopt and extend the UTAUT to investigate millennials' adoption of autonomous vehicles by considering the importance of technology adoption, perception of benefits, security, safety, mobility-related efficiencies, and concerns. Rahman et al. (2019), in turn, investigate older adults' (age > 60) perception of self-driving vehicles from the perspective of users and pedestrians as well as their willingness to use them.

Panagiotopoulos and Dimitrakopoulos (2018) enrich the TAM (Davis et al., 1989) with the constructs 'trust' and 'social influence' to understand behavioral intentions toward using autonomous vehicles. Choi and Ji (2015) found predictors for the intention to use autonomous driving, manifested as trust in and perceived usefulness of autonomous driving. In addition, Xu et al. (2018) added the trust construct to the technological acceptance model to study user acceptance of autonomous driving, showing that trust is positively related to behavioral intention. Based on causal models of trust, Liu et al. (2018) investigate how social trust shapes general public acceptance, willingness to pay, and intention to use autonomous vehicles. Zhang et al. (2020) study the role of social and personal factors like trust for automated vehicle SAE level 3 acceptance in China, while Zhang et al. (2019) also investigate the relation of perceived risk on the public's acceptance. Next to trust, Hegner et al. (2019) also consider aspects of control, personality characteristics, and extrinsic and intrinsic motivations in investigating the acceptance of autonomous vehicles. Buckley et al. (2018a) apply the theory of planned behavior and the TAM to understand the willingness to use autonomous vehicles in a simulated driving task. The researchers found that attitudes, subjective norm, perceived behavior control, and trust are significant factors predicting the intention to use autonomous vehicles.

Lee et al. (2019) reveal that the perception of ownership (i.e., owning autonomous vehicles vs. use of existing car-sharing services) and the ability to manage prospective situations influence the potential use of autonomous vehicles. Haboucha et al. (2017) investigate individual motivations for choosing to own and use autonomous vehicles and develop a model for autonomous vehicle long-term choice decision. Acheampong and Cugurullo (2019) investigate adoption decisions regarding three different autonomous vehicle modes—ownership, sharing, and public transport—extending the TAM by factors of perceived benefits, fears and anxieties, subjective norm, image, and perceived behavior control. Motamedi et al. (2020) study the user acceptance of personally owned and shared-use autonomous driving concepts. The researchers found, for example, that, in comparison to shared-use concepts, perceived usefulness, compatibility, and trust were shown to have a higher significant impact on intentions to use personally-owned vehicles, while perceived ease of use was found to be less important in predicting user acceptance. Paddeu et al. (2020) identify comfort and trust as relevant factors in predicting users' acceptance of shared autonomous vehicles. Madigan et al. (2017) use the UTAUT to investigate public acceptance of autonomous vehicles SAE level 4 for road transport systems. Using the same theoretical framework, Kaur and Rampersad (2018) investigate key adoption factors of driverless cars as a public transportation service in closed environments. Bernhard et al. (2020) explore the acceptance of an autonomous minibus in Germany. The researchers reveal that participants perceive the driving experience with an autonomous minibus as generally positive and that performance expectancy, experience, and degree of pleasure are relevant predictors of acceptance of automated public transport.

Wu et al. (2019) rely on the TAM to understand the impact of environmental concerns on public acceptance of autonomous electric vehicles. Koul and Eydgahi (2018) use the TAM as a theoretical framework to study autonomous driving acceptance, revealing a statistically positive relationship between perceived usefulness, perceived ease of use, and intention to use driverless cars and a statistically negative relationship between years of driving experience, age, and intention to use autonomous vehicles.

Using different scenarios, Bansal and Kockelman (2017) propose a simulation-based fleet evolution framework to assess the long-term adoption levels of connected autonomous vehicles in the U.S. Assuming an annual 5% price drop and constant willingness-to-pay values, the researchers forecast that the privately held light duty vehicle fleet will have a 24.8% autonomous vehicle penetration by 2045. This share is likely to increase with declining prices and higher willingness-to-pay values. On a related note, Daziano et al. (2017) reveal that the average U.S. household is willing to pay $3500 for partial automation (e.g., automated crash avoidance) and $4900 for fully automated technology (e.g., Google car). In addition, they show that a significant share of respondents is willing to pay above $10,000 for fully automated self-driving vehicles.

Using a descriptive analysis approach, Howard and Dai (2014) investigate public attitudes toward self-driving cars. Nordhoff et al. (2018) also examine acceptance of driverless vehicles and Schoettle and Sivak (2014) study public opinions of autonomous and self-driving vehicles in the U.S., the U.K., and Australia. Talebian and Mishra (2018) combine the theories of diffusion of innovations and agent-based modeling and use a descriptive approach to investigate how individuals utilize their social networks when evaluating the purchase of autonomous vehicles. They emphasize that perceived barriers related to autonomous vehicles can be reduced through peer-to-peer communication with satisfied adopters. Furthermore, Leicht et al. (2018) analyze the moderating effects of consumer innovativeness on the relationships between different antecedents of autonomous vehicle purchase intention by extending the UTAUT. Using the same framework, Benleulmi and Becker (2017) investigate drivers and inhibitors of autonomous cars' acceptance across cultures.

Research has also linked the topic of safety to the acceptance of autonomous vehicles. Hulse et al. (2018), for example, show that autonomous vehicles are perceived as riskier (in terms of collision and

injury) from the point of view of passengers compared to pedestrians. However, the study concludes that, compared to existing modes of transportation, autonomous vehicles are generally rated well in terms of risk perception and attitudes. Ro and Ha (2019) show that expectations regarding convenience and safety significantly influence both attitude and intention to use autonomous vehicles positively.

In summary, previous research has revealed relevant factors that influence the perception of autonomous driving. A number of studies rely on the TAM when investigating these factors, indicating the applicability of this model to investigate the acceptance of autonomous driving (e.g., Lee et al., 2019; Panagiotopoulos and Dimitrakopoulos, 2018; Wu et al., 2019). Nevertheless, there is substantial criticism of the technology acceptance research. It has been stated that using a simple TAM could lead researchers to overlook central influencing factors of technology acceptance. This in turn has resulted in a call for further extensions of the model to increase its overall explanatory power as well as understanding of each determinant (Bagozzi 2007; Benbasat and Barki 2007). In addition, Benbasat and Barki (2007), Bagozzi (2007), and Yang and Yoo (2004) emphasize that acceptance research should integrate constructs from the theory of planned behavior (Ajzen, 1991), such as the attitude construct. However, the literature review showed that the majority of TAM studies only integrate a limited number of additional study-specific factors (e.g., trust) to understand the adoption process of autonomous driving. Most of these studies also neglect to integrate the attitude construct to account for prediction of intentions and, thus, actual behavior (e.g., Choi and Ji, 2015; Hegener et al., 2019; Koul and Eydgahi, 2018). Additionally, the examined studies primarily rely on theoretical and/or literature-based approaches to identify these factors (e.g., Hegner et al., 2019; Lee et al., 2019; Wu et al., 2019). With this approach, relevant constructs from an end-user point of view may be overlooked (Venkatesh et al., 2013; Wu, 2012). To overcome these limitations, Venkatesh et al. (2013) suggest enhancing the TAM by using exploratory qualitative studies to reveal relevant factors to individuals' acceptance and then analyze them and their relationships quantitatively. Qualitative research can provide details to underpin the perception of autonomous driving and a deeper understanding of necessary constructs to be used in quantitative research (Buckley et al., 2018b). Moreover, qualitative methods are a powerful tool for exploring increased complexity in the field of travel behavior, as they allow for an understanding of one's own explanations of behavior and attitudes (Clifton and Handy, 2003). However, only a limited number of studies investigate this research field qualitatively. For example, based on 13 in-depth interviews, Bjørner (2015) explores the a priori acceptance and perceived driving pleasure of autonomous driving, revealing that parking assistance systems and the use of assistance systems when driving in city traffic positively affect attitudes toward autonomous driving systems. Buckley et al. (2018b) shows that trust (conceptualized as the vehicle's ability to meet desired actions, the relevance and usefulness of autonomous vehicles, and integrity in terms of hacking and privacy), affective responses, financial costs, efficacy, identity (e.g., as an early technology adopter), and social norms shape the perception of autonomous vehicles. In the same vein, Nordhoff et al. (2019) investigate impressions of users after riding in an automated shuttle.

3. Research model and hypotheses development

The TAM remains a reliable model for investigating the acceptance of technological innovations and has been employed for examining autonomous driving (Choi and Ji, 2015; Lee et al., 2019; Panagiotopoulos and Dimitrakopoulos, 2018; Wu et al., 2019). However, given the limitations outlined in the previous section, we aim to extend existing TAM research by deriving a set of end-user-focused constructs to understand autonomous driving acceptance. Therefore, we adhere to Venkatesh et al.'s (2013) suggestion of applying a mixed-method approach. Initially, we conducted interviews to understand further acceptance factors that might not have been considered through the standardized TAM. The corresponding literature on autonomous driving and acceptance was then analyzed to develop hypotheses geared toward the interview-derived acceptance criteria, developing appropriate constructs.

3.1. Interviews

Qualitative methods have the ability to generate a knowledge base with regard to those factors that determine whether a new product or service is eventually approved or rejected. Particularly in an innovation's initial market launch, qualitative methods can effectively identify any existing preferences relating to the use of this product, as well as any barriers encountered (Kleijnen et al., 2009). One of the greatest advantages of qualitative methods is their ability to identify a phenomenon that was previously unknown (Mayring, 2014). While it can be argued that quantitative approaches are better suited to the testing of hypotheses, qualitative methods retain the capacity to disclose novel hypotheses and concepts for the investigative field. Therefore, qualitative methods likewise assist in producing new insights for neglected research areas, rather than validating already present knowledge and information (Mayring, 2014). Several current acceptance studies within the field of mobility research use different types of mixed methods approaches in order to derive a sufficiently firm basis for suitable acceptance criteria (e.g.,; Distler et al., 2018; Kilian-Yasin et al., 2016).

We used semi-structured interviews (Hopf, 2004) to explore potential acceptance criteria for autonomous vehicle usage. In the same vein as existing research (e.g., Grimsley and Meehan, 2007), we pursued a developmental purpose (Venkatesh et al., 2013). We recruited participants for the interviews via direct contacts and social network announcements in Germany. The qualitative study drew on a sample of 20 participants, who ranged in age from 19 to 65 years. In our iterative process of analyzing the interview data, we found that a theoretical saturation (Creswell, 2007) occurred within the first 20 interviews. Thus, we decided to stop the recruitment of new interviewees. All sociodemographic details are summarized in Table 1.

Although several studies have investigated public opinion and willingness to use autonomous vehicles (e.g., Penmetsa et al., 2019), there are contradictory views on the representativeness of participants that should be included to study the acceptance related to autonomous driving (see Kyriakidis et al., 2015; Nordhoff et al., 2016 for a review), indicating that potential adopters of autonomous driving cannot be limited to a specific group.

In this context, Nordhoff et al. (2016) conclude that most studies focus on car drivers as the target population and suggest that studies need to include a variety of other potential adopters, including underserved groups, such as the elderly. On a related note, the focus on specific group participants in the context of automated driving has also been criticized by Koerber and Bengler (2014). We therefore argue that our subjects represent a relevant target group for the research context, due to the variety of their sociodemographic characteristics.

The interviews were mainly conducted face-to-face, were recorded, and lasted, on average, 40 min. After introducing participants to the aim of the study and collecting sociodemographic data, we showed participants a short video about autonomous driving and provided the following definition of an autonomous vehicle with level 5 automation to ensure an equal understanding of the topic among participants: "The vehicle's system can handle all situations autonomously. Passengers are not required to take over control of the vehicle during a trip." The video and the definition did not include evaluative elements or statements about advantages and disadvantages of autonomous driving to minimize influence on participants' statements. Users were also not given a specific context for usage, that is, whether autonomous vehicles were privately owned or used as a shared service.

We then asked the following questions about the potential

Table 1.
Sample details of interview participants.

Personal characteristic	Response category	*n*
Gender	Male	11
	Female	9
Age	< 20	1
	20–29	4
	30–39	3
	40–49	5
	50–59	4
	> = 60	3
Driver's license	Yes	18
	No	2
Accident experience	Yes (at least one with a car)	11
	No	9
Experience with driver assistance systems	Yes	12
	No	8
Highest education	School leaving examination	7
	Completed higher education studies	5
	Pupil, student, apprentice	8
Living situation	Rural area	8
	Urban area	12
Annual kilometers traveled by car	> 5000 km	3
	5001–10,000 km	6
	10,001–15,000 km	6
	15,001–20,000 km	3
	> 20,000 km	2
Use of public transport	Daily	6
	Several times a week	8
	Several times a month	4
	Rarely	2
	Never	–

advantages and disadvantages of autonomous driving. Based on the participant's answers, the interviewer asked for more detailed information on the topic at hand. (1) What do you associate with autonomous driving? (2) Where do you see the benefits of autonomous driving and what do you expect from it? (3) Where do you see barriers to autonomous driving and what would prevent you from using autonomous driving? (4) Which requirements of autonomous driving should be met in order to make you feel comfortable when using this form of transportation in the future?

To transform the recorded data into a standardized form, we performed qualitative content analysis, following Mayring (2014). Before conducting and analyzing the interviews, we pre-defined potential acceptance criteria, based on general and context-specific literature of acceptance research (e.g., Agarwal and Prasad, 1998; Ajzen, 1991; Burger and Cooper, 1979; Davis et al., 1989, 1992; Nordhoff et al., 2016; Venkatesh and Bala, 2008; Venkatesh and Davis, 2000).

First, we decided on the content-analytical method. We chose the structuring (deductive category assignment) method because we wanted to assign the context unit to pre-defined potential acceptance criteria that we derived from previous research (Mayring, 2014). According to Mayring (2014), this approach is particularly appropriate if there exist relevant previous research/theories within the topic of interest. We argue that this assumption is made for the following two reasons. First, acceptance research (including acceptance criteria) is an intensively studied and well-established field of research. Second, we pre-defined a theoretical acceptance model (see Fig. 1) that serves as a foundation for assigning the derived acceptance criteria.

Second, we tested the interview questionnaire with three subjects. These data were not included in the study and were only used for testing purposes. The pre-test led to minor adjustments in the questionnaire, that is, minor changes in the wording of the questionnaire to ensure understandability. In addition, the three test interviews were used to discuss and create an initial coding guide. The coding guideline contained a precise definition of acceptance criteria derived from the literature, sample statements from the test interviews, and the coding rules. The coding rules included two parts. First, interview statements were assigned to an acceptance category if they matched the definition of the category in terms of content (e.g., when the interview statement and category definition included similar or identical groups of words). Second, statements that could not be exactly matched in that way were put aside for joint discussion within the research team.

Third, we conducted the interviews. All interviews were conducted by the same person. The pre-test helped to address bias issues by allowing elimination of difficult or ambiguous questions by assessing whether each question was able to give an adequate range of responses, and by ensuring that responses could be interpreted in terms of the research aim (Chenail, 2011). The interviews were recorded and transcribed in typed form. The transcribed statements were condensed; that is, all statements that were not related to the research objective, repetitions, incomplete sentences, and filler words were deleted. The resulting core text formed the foundation of the analysis (context unit).

Fourth, the data analyses were conducted by two researchers. A fully crossed design was applied for data analysis; in other words, all responses were assessed independently by the two researchers (Hallgren, 2012). Both researchers have considerable expertise in acceptance research and autonomous driving.

Finally, as suggested by Mayring (2014), the research team performed a trial encoding from the first three interviews. After revising the matching process and ensuring that the research team had a common understanding of the data analysis procedure, the first three interviews were re-coded again. A repetitive revision and discussion of the category system was carried out after encoding three further interviews.

We argue that inter-rater reliability was not a threat to our study for the following three reasons. First, we followed a deductive category development approach. Therefore, the analysis relied on a solid theoretical ground which does not allow much space for subjective interpretations (Potter and Levine-Donnerstein, 1999). In this context, the task of matching statements to pre-defined categories is less complex compared to, for example, an inductive approach in which no

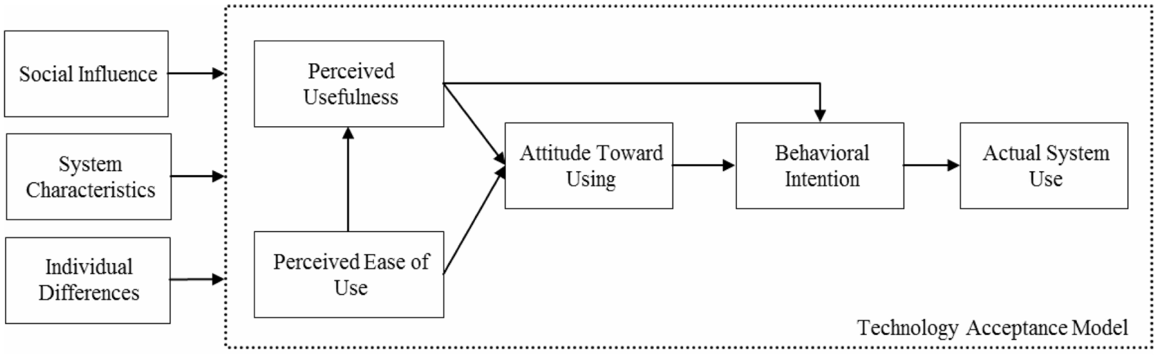

Fig. 1. Theoretical framework (adapted from Venkatesh and Bala, 2008).

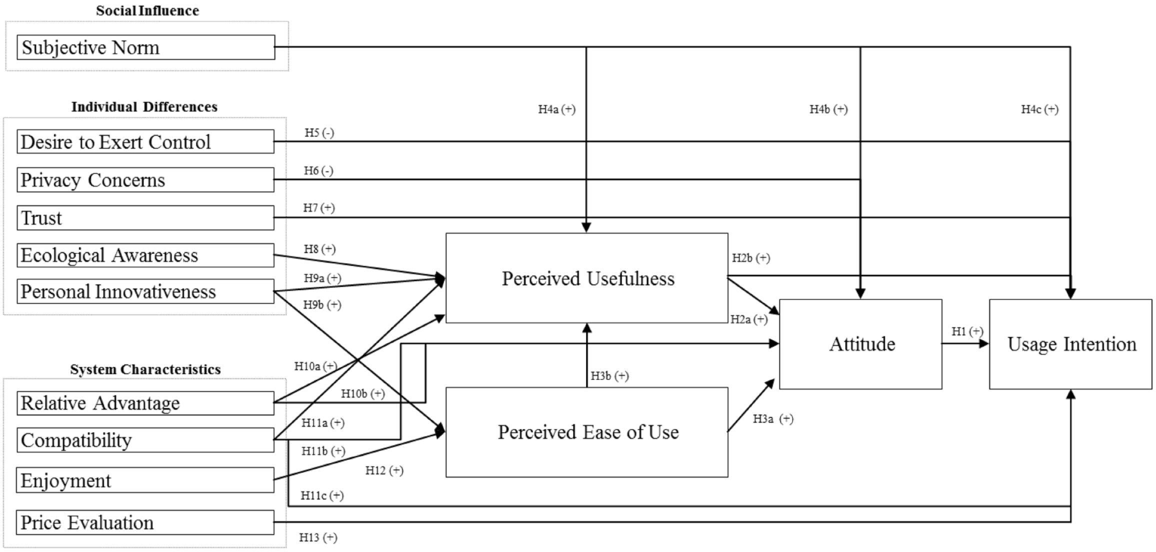

Fig. 2. Proposed research model for investigating the acceptance of autonomous driving.

identifiable theory guides the design of the analysis. Second, both researchers jointly critically reviewed and discussed the results of the coding after every three analyzed interviews. Third, there was no noteworthy dissent between the coders. In cases where coders were not sure whether a statement could be categorized, further experts in the fields of acceptance research and autonomous driving were consulted.

The qualitative interviews revealed 10 acceptance criteria, which became the foundation for hypothesis development: desire to exert control, perceived enjoyment, trust, price evaluation, ecological awareness, privacy concerns, subjective norm, personal innovativeness, relative advantage, and compatibility. The results of the interviews and definitions of the related constructs are presented in Appendix B.

3.2. Theoretical framework

The TAM is an adaptation of the theory of reasoned action (Ajzen and Fishbein, 1980) and aims to provide an explanation of the determinants for the acceptance of a technology (Davis et al., 1989). The TAM is subject to a basic assumption of the actual behavior of an individual, determined via the technology's intended use, aligned directly with the reasoned action theory, and, above all, influenced by the attitude toward the technology. Two main theoretical constructs, perceived usefulness and ease of use, are posited to explain a technology and its subsequent acceptance. Perceived usefulness can refer to the presence of a positive use– performance relationship, while at the same time perceived ease of use is the degree to which an individual expects that using a specific system will be free of effort (Davis et al., 1989). According to Davis et al. (1989), perceived usefulness is likely to influence attitude because positively evaluated performance outcomes can increase an individual's favorable opinion toward the technology to achieve these outcomes. Perceived ease of use is posited to influence the attitude because, when a system is easy to use, it enhances the individual's perceived sense of efficacy and personal control with regard to the ability to carry out actions needed to operate a technology. In addition, Davis et al. (1989) point out that perceived usefulness impacts behavioral intentions because behavioral intentions are mainly shaped by cognitive appraisals of the degree to which a technology improves the individual's performance. Finally, perceived usefulness is influenced by perceived ease of use. When a technology is easier to use, the effort saved as a result can be redeployed, which makes the technology more useful.

The TAM is a powerful and robust method to predict the acceptance of a variety of technologies, and compares favorably to alternative models, such as the theory of reasoned action or the theory of planned behavior; it typically explains about 40% of the variance in behavior-related intentions and actual behavior (King and He, 2006; Venkatesh, 2000). According to approximations from the TAM and the central determinants that influence it, we formed a theoretical framework applicable to the proposed research model (see Fig. 1).

Synthesizing prior research on acceptance, Venkatesh and Bala (2008) derive four different determinants (social influence, individual differences, system characteristics, and facilitating conditions) that influence the TAM, particularly the constructs of perceived usefulness and perceived ease of use. “Social influence” represents social processes (e.g., the opinion of important referents) that influence the individual's perception of a technology. “Individual differences” refer to the individual's personality, for example, certain traits and beliefs that might influence the perception of a technology. “System characteristics” are salient features of a technology that aid in forming the individual's favorable or unfavorable perceptions of certain aspects of the system. Finally, “facilitating conditions” reflect organizational support that eases the use of a technology. We excluded facilitating conditions as a determinant, as the organizational setting is not the focus of our research. The remaining categories serve as a framework for better structuring and allocating the results of the interviews in order to derive the proper extended determinants from an end-user's point of view.

3.3. Hypothesis development

Regarding the interview-based identification of factors that potentially influence the acceptance of autonomous driving, we screened existing literature on autonomous driving and acceptance research to develop the proposed hypotheses. Relying on the proposed framework (Fig. 1), we matched the interview-based criteria with the three main categories—individual differences, system characteristics, and social influence. The proposed research model is illustrated in Fig. 2.

3.3.1. Relationships within the TAM

The TAM is the main foundation of the research model. Since autonomous driving is still not available to the general public for everyday use, we removed the construct “actual system use” from the research model's methods. We use the construct “behavioural intention” as a final output of the model, which is demonstrated to be a reliable predictor of actual system use (Chin et al., 2008; Lee et al., 2003; Pai and Huang, 2011; Venkatesh et al., 2007). In alignment with the TAM and the theory of reasoned action (Ajzen and Fishbein, 1980), we posit that

behavioral intention is predicted by the attitude toward using autonomous vehicles. This causal relationship is present throughout other studies as well (e.g., Nysveen et al., 2005; Payre et al., 2014). The original TAM theorizes perceived usefulness and perceived ease of use to be the central determinants of general attitude to a technology's use. In addition, the beneficial relationship of perceived usefulness and perceived ease of use in relation to usage intention can be assumed. Likewise, perceived usefulness is assumed to be beneficially associated with perceived ease of use. Apart from the organizational field of application, the posited relationships have also been shown to be relevant in the context of consumers and autonomous driving (e.g., Bruner and Kumar, 2005; Choi and Ji, 2015; Lee et al., 2019; Nysveen et al., 2005; Panagiotopoulos and Dimitrakopoulos, 2018; Pedersen, 2005; Wu and Wang, 2005; Wu et al., 2019). We summarize our assumptions in the following hypotheses:

Hypothesis 1. The more positive the attitude toward autonomous driving, the higher the usage intention of autonomous driving.

Hypothesis 2a. The higher the perceived usefulness of autonomous driving, the more favorable the attitude toward autonomous driving.

Hypothesis 2b. The higher the perceived usefulness of autonomous driving, the higher the usage intention of autonomous driving.

Hypothesis 3a. The higher the perceived ease of use of autonomous driving, the more favorable the attitude toward autonomous driving.

Hypothesis 3b. The higher the perceived ease of use of autonomous driving, the greater the perceived usefulness of autonomous driving.

3.3.2. Relationship between social influence and the TAM

Research has demonstrated the influential consequences of an individual's subjective norm on perceived usefulness, performing a specific behavior and the intention to do so (Davis et al., 1989; Venkatesh and Davis, 2000). Moreover, Schepers and Wetzels's (2007) meta analysis reveals that the positive effect of subjective norm on intention to use is accompanied by a beneficial effect on the perceived usefulness of an innovation. They explain that subjective norm is likely to influence perceived usefulness because of a prevalent tendency for individuals to rely on information from important others as evidence about reality. In this regard, Thorpe and Motwani (2017) emphasize the importance of the individual's subjective norm in the success of autonomous driving because individuals tend to follow what other people actually do or think. The positive opinion of others about the usefulness of autonomous driving can therefore influence people's own perception of the utility of autonomous driving. Acheampong and Cugurullo (2019) found, for example, that subjective norm has a positive effect on the perceived benefits of autonomous vehicles. Buckley et al. (2018a) found that subjective norm is a significant factor for predicting the intention to use autonomous vehicles. Similarly, Panagiotopoulos and Dimitrakopoulos (2018) show that social influence affects the behavioral intention to use autonomous vehicles. Talebian and Mishra (2018) emphasize that perceived barriers related to autonomous vehicles can be reduced through peer-to-peer communication with satisfied adopters within their social network. Bansal et al. (2016) conducted a study that examined which social factors influence the acceptance and adoption of autonomous driving. The authors found that participants preferred to use autonomous driving when the technology was adopted by family members, friends, or neighbors. Hence, we put forth the subsequent hypotheses:

Hypothesis 4a. Subjective norm positively influences the perceived usefulness of autonomous driving.

Hypothesis 4b. Subjective norm positively influences attitudes toward using autonomous vehicles.

Hypothesis 4c. Subjective norm positively influences the intention to use autonomous vehicles.

3.3.3. Relationships between individual differences and the TAM

The perception of control is a fundamental aspect of autonomous driving. Research has shown that perceived lack of control by drivers accompanies an increased level of vehicle autonomy (Roedel et al., 2014). Eckoldt et al. (2012) reveal that the lack of vehicle control due to advanced driving assistance systems is perceived as a challenging endeavor by the individuals involved, and hence advances to a “distance between driver and car” (p. 167). The negative perception related to the loss of control is especially prevalent when driver experience with advanced driving assistance systems is low (Planing, 2014). Heiskanen et al. (2007) emphasize that a reduced perception of control due to technologies leads to a “loss of individual choice and the freedom to follow one's impulses” (p. 503). Since autonomous driving relies heavily on advanced driver-assistance systems, it can be assumed that a greater desire to exert control is negatively correlated with the intention to use autonomous driving.

Hypothesis 5. The greater the desire to exert control, the lower the intention to use autonomous vehicles.

Another factor impacting the overall acceptance of technological innovations is privacy concerns. Regarding autonomous vehicles and their increasing connectivity due to information systems, drivers themselves remain especially hesitant about in-vehicle technological connectivity, one example being the possibility of having their location revealed at any time (Mahoney and O'Sullivan, 2013). This effect is strengthened by the fact that control systems within modern cars are seen as fragile, and an easy target for hacking (Koscher et al., 2010). Research reveals that privacy concerns negatively influence the decision-making process, increasing reluctance (Nasri and Charfeddine, 2012; Phelps et al., 2000). We thus argue that privacy concerns are negatively associated with attitudes toward using autonomous vehicles and propose the following hypothesis:

Hypothesis 6. Privacy concerns associated with autonomous driving negatively influence attitudes toward autonomous driving.

Autonomous vehicles control steering, acceleration, and deceleration completely by built-in automated systems. Thus, trust in systems such as these is generally seen as a relevant determinant for the approved reception of autonomous driving (Adnan et al., 2018; Buckley et al., 2018a, b; Choi and Ji, 2015; Hengstler et al., 2016; Hohenberger et al., 2017). Adnan et al. (2018) studied the impact of trust on user acceptance of autonomous vehicles and found that the level of trust depends on sociodemographic user profiles. Choi and Ji (2015) ascertain that trust in autonomous driving is reflected in three dimensions: *system transparency* reflects the degree to which individuals understand how autonomous vehicles operate; *technical competence* is the process by which individuals assess the technical processes of autonomous vehicles; and *situational management* is an individual's belief as to their agency in recovering control in any situation during the drive. The authors reveal a beneficial influence of trust on the intention to use autonomous vehicles. Therefore, the following hypothesis is proposed:

Hypothesis 7. The higher the trust in autonomous driving, the greater the intention to use autonomous vehicles.

A further personality component constitutes the ecological awareness of potential end-users. In the vehicular context, it has already been revealed that the purchase of environmentally friendly vehicles mainly relies on the assessment of ecological aspects (see Peters et al., 2011). In alignment with the findings of Thongmak (2012), we argue that ecological awareness strengthens the perceived usefulness of autonomous driving by potentially, for example, counteracting global warming, reducing toxic emissions, or diminishing resource usage. The ecological potential of autonomous driving can be seen in the reduction of carbon dioxide emissions and the reduction of fuel consumption by optimizing traffic flow through less frequent braking and acceleration (Anderson et al., 2016; Brown et al., 2014). Shanker et al. (2013) claim

that autonomous vehicles are 30% more efficient than conventional ones. Wu et al. (2019) show that environmental concerns affect all factors: green perceived usefulness, perceived ease of use, and behavior intention of autonomous electric vehicles. This is in line with Grant-Muller and Usher (2014), who also state that intelligent transport systems will have environmental benefits through increased vehicle efficiency. We summarize our assumption in the following hypothesis:

Hypothesis 8. The higher the ecological awareness, the greater the perceived usefulness of autonomous driving.

Agawal and Prasad (1998) state that personal innovativeness remains crucial in the examination of technological innovations' acceptance. Following Midgley and Dowling (1993), we apply innovativeness in a context-specific manner, that is, personal innovativeness related to autonomous driving, rather than focusing on general personal innovativeness. By doing so, we relate personal innovativeness to the willingness of individuals to try new driver assistance systems with a high degree of automation. Various applicable studies demonstrate the overall beneficial influence of personal innovativeness on perceived usefulness and perceived ease of use (Kuo and Yen, 2009; Lin et al., 2007; Mao et al., 2005). Correspondingly, we suggest the following two hypotheses:

Hypothesis 9a. The higher the personal innovativeness regarding autonomous driving, the higher the perceived usefulness of autonomous driving.

Hypothesis 9b. The higher the personal innovativeness regarding autonomous driving, the higher the perceived ease of use of autonomous driving.

3.3.4. *Relationships between system characteristics and the TAM*

Tornatzky and Klein's (1982) meta analysis on innovation characteristics reveals an advantage, in this case relevant to the analysis, which simultaneously reveals the possibility of influencing the adoption decision. Kulviwat et al. (2007) show that individuals evaluate an innovation as more useful with increasing perception of the innovation as being better than its precursor. In addition, the authors argue that relative advantage retains a positive relation to attitude toward performing a behavior, specifically when compared to the organizational context; consumers have the agency and free will to compare and contrast the characteristics of an innovation within the decision-making process. Furthermore, Lee et al. (2019) show that relative advantage positively affects the perceived usefulness of autonomous vehicles. Hence, we argue for the following two hypotheses:

Hypothesis 10a. The higher the perceived relative advantage of autonomous driving, the greater the perceived usefulness of autonomous driving.

Hypothesis 10b. The higher the perceived relative advantage of autonomous driving, the more positive the attitude toward autonomous driving.

Compatibility is another relevant factor in the decision to adopt (Tornatzky and Klein, 1982). In the context of autonomous driving, we define compatibility as the extent to which autonomous driving accords with the individual's usual mobility behavior in terms of daily driving practice or traveling. The effect of the compatibility construct on the TAM constructs is ambiguous. Teo and Pok (2003), for example, demonstrate the positive effect of compatibility on attitude, while Karahanna et al. (2006) reveal a positive correlation between compatibility and attitude, as well as perceived usefulness and intention to use. A study conducted by Wu and Wang (2005) demonstrates how compatibility significantly influences perceived usefulness and intention to use; hence, we propose the following hypotheses:

Hypothesis 11a. The higher the compatibility of autonomous driving with the potential user's mobility needs, the greater the perceived usefulness of autonomous driving.

Hypothesis 11b. The higher the compatibility of autonomous driving with the potential user's mobility needs, the more favorable the perceived attitude toward autonomous driving.

Hypothesis 11c. The higher the compatibility of autonomous driving with the potential user's mobility needs, the greater the perceived intention to use autonomous driving.

Venkatesh and Bala (2008) emphasize the ways in which enjoyment fails to enhance the benefits when operating a system. Nevertheless, enjoyment reduces difficulty for individuals when there is an interaction with an applicable system, since some pleasure and enjoyment can be derived from the act itself. The beneficial elements of perceived enjoyment to perceived ease of use have been reinforced by several studies within various research contexts (e.g., Fagan et al., 2008; Saadé and Bali, 2005; Sun and Zhang, 2006; Venkatesh, 2000). Consequently, we hypothesize that:

Hypothesis 12. The greater the perceived enjoyment of autonomous driving, the greater the perceived ease of use of autonomous driving.

To determine the perceived worth of a product or service, the monetary price of that product or service is usually assessed together with its quality (Zeithaml, 1988). Venkatesh et al. (2012) emphasize that the value of the price is an essential step in explaining a technology's acceptance in the consumer-use setting, as the consumer usually bears the cost of using the technology (unlike in the organizational setting). They state that the "price value is positive when the benefits of using a technology are perceived to be greater than the monetary cost and such price value has a positive impact on intention" (p. 161). Bansal and Kockelman (2017) show that the penetration rate of future autonomous vehicles depends largely on reducing the cost of the technology. They argue that successful penetration is accompanied by the need to increase consumers' willingness to pay or to improve the price/performance ratio, for example, through political subsidies. Daziano et al. (2017) state that households are very different in their willingness to pay for autonomous driving. We follow these ideas and assume that the impact of price value on the intention to use autonomous driving is positive, specifically when the benefit of autonomous driving is greater than the beneficial monetary price of autonomous driving (positive price value). We propose the following hypothesis:

Hypothesis 13. The higher the (positive) price evaluation, the greater the intention to use autonomous driving.

4. Materials and methods

Based on the developed research model, we designed and conducted an online survey with the *SoSci Survey* software package to test the proposed hypotheses. Participants were recruited via direct acquisition, announcements at university lectures, and social network announcements.

4.1. *Participants*

In total, the study drew on a sample of 316 participants, of which 32.70% were women. All sociodemographic details of the participants are summarized in Table 2. Owing to the variety of participants' sociodemographic characteristics, we argue that our subjects represent a relevant target group for the overall research context (see Section 3.1 for a more detailed discussion).

4.2. *Measurement of constructs*

The respective constructs, number of measures, definition of constructs, and related references from which the items and definitions were adapted are presented in Appendix C. All items are based on a seven-point Likert scale ranging from 1 (totally disagree) to 7 (totally agree).

Table 2
Sample details of survey participants.

Personal characteristic	Response category	*n*
Gender	Male	212
	Female	104
Age	18–25	70
	26–30	69
	31–40	45
	41–50	30
	51–60	45
	61–70	37
	> 70	18
Driver's license	Yes	313
	No	3
Accident experience	Yes (at least one with a car)	165
	No	151
Experience with driver assistance systems	Yes	175
	No	121
Highest education	Completed academic studies	188
	General qualification for university entrance	64
	Pupil, student, apprentice	64
Living conditions	Urban areas	203
	Rural areas	113
Income	< 1500 EUR	74
	1501–2000 EUR	34
	2001–2500 EUR	24
	2501–3000 EUR	21
	3001–4000 EUR	52
	> 4000 EUR	75
	No information	32
Annual kilometers traveled by car	> 5000 km	61
	5001–10,000 km	62
	10,001–15,000 km	66
	15,001–20,000 km	60
	> 20,000 km	66
Use of public transport	Daily	51
	Several times a week	68
	Several times a month	44
	Rarely	118
	Never	35

5. Data analysis and results

We used variance-based partial least structural equation modeling (Lohmoeller, 1989) to test the proposed research model. We applied the widely adopted two-step modeling approach (Anderson and Gerbing, 1988) for data analysis, as implemented in the SmartPLS 2.0.M3 software. By doing so, we first assessed the quality of the measures and then tested the structural model. For both, we followed the guidelines for PLS estimations as suggested by Hair et al. (2012).

5.1. Validation of measurements

We first checked the survey data for the thread of common method bias, as all measures were assessed by a single source of information. We applied Harman's single-factor test to check the presence of common method bias and ran an exploratory factor analysis (Podsakoff et al., 2003). No single factor emerged from the factor analysis and no general factor accounts for the majority of variance among the measures. Therefore, we argue that common method bias is not of great concern to our study.

To evaluate the quality of the measurement model, we assessed content, convergent, and discriminant validities (Fornell and Larcker, 1981; Hair et al., 2011). We used validated items from previous studies; hence, we argue that content validity is given.

For convergent validity, three sectors were assessed: average variance extracted (AVE), composite construct reliability (CR), and individual item reliability (Fornell and Larcker, 1981). We tested individual-indicator reliability by extracting the factor and cross-loading all items to their respective latent constructs. Owing to low factor loadings, one item was dropped from each of the constructs of *price evaluation, desire to exert control, perceived ease of use*, and *personal innovativeness*. After this, all items loaded on their respective construct higher than the acceptable threshold of 0.60 (Bagozzi and Yi, 1988; Chin, 1998). Moreover, all items loaded on their own construct more highly than on any different construct, and each item's factor loading on its respective construct was significant ($p < 0.01$). The results of the factor and cross-loadings are presented in Appendix D. The CR ranged from 0.89 to 0.99, exceeding the recommended threshold of 0.70, while the variance shared between all constructs and their measures (AVE) was above the acceptable lower bound of 0.50 (Bagozzi and Yi, 1988; Bhattacherjee and Premkumar, 2004).

Finally, Fornell and Larcker's (1981) criterion was enabled for discriminant validity to be ascertained. The AVE for each measure exceeded 0.50 and the square root of the AVE was found to be greater than the respective construct correlations, thus confirming discriminant validity. The results are summarized in Table 3.

5.2. Hypothesis testing

Since the measurement analyses suggest that the model is both acceptable and reliable, we applied the bootstrapping re-sampling procedure (Chin, 1998) to assess the structural path of the proposed research model with a bootstrapping sample size of $n = 5000$ (Hair et al., 2011). Table 4 and Fig. 3 present the results of the structural model estimations.

Table 3
CA, AVE, CR, and inter-construct correlations.

	Construct	CA	AVE	CR	1	2	3	4	5	6	7	8	9	10	11	12	13	14
1	Attitude	0.94	0.79	0.96	**0.89**													
2	Compability	0.89	0.83	0.93	0.70	**0.91**												
3	Ecological Awareness	0.85	0.68	0.90	0.13	0.13	**0.83**											
4	Desire to Exert Control	0.81	0.73	0.89	−0.45	−0.45	−0.02	**0.85**										
5	Enjoyment	0.86	0.78	0.91	0.79	0.66	0.19	−0.38	**0.88**									
6	Privacy Concerns	0.91	0.79	0.94	0.34	0.37	0.03	−0.35	0.39	**0.89**								
7	Price Evaluation	0.89	0.82	0.93	0.67	0.64	0.11	−0.39	0.65	0.34	**0.91**							
8	Perceived Ease of Use	0.84	0.76	0.90	0.56	0.48	0.05	−0.39	0.45	0.35	0.40	**0.87**						
9	Personal Innovativeness	0.86	0.64	0.90	0.39	0.44	0.11	−0.23	0.37	0.23	0.38	0.31	**0.82**					
10	Perceived Usefulness	0.85	0.68	0.90	0.80	0.68	0.14	−0.42	0.71	0.32	0.68	0.52	0.43	**0.83**				
11	Relative Advantage	0.87	0.66	0.90	0.81	0.66	0.17	−0.40	0.76	0.37	0.66	0.56	0.40	0.81	**0.89**			
12	Subjective Norm	0.92	0.81	0.94	0.60	0.57	0.13	−0.36	0.56	0.32	0.51	0.44	0.33	0.63	0.63	**0.90**		
13	Trust	0.98	0.92	0.99	0.74	0.64	0.14	−0.55	0.68	0.40	0.63	0.60	0.44	0.76	0.75	0.55	**0.96**	
14	Usage Intention	0.87	0.74	0.92	0.81	0.67	0.11	−0.45	0.72	0.32	0.64	0.46	0.37	0.71	0.68	0.57	0.71	**0.86**

Note. CA: Cronbach's Alpha; bolded numbers: square root of AVE.

Table 4
Structural equation model statistics.

Proposed hypotheses<br>Relationships within the TAM		b	t-value	*p*-value	Conclusion
H1:	The more positive the attitude toward autonomous driving, the higher the usage intention of autonomous driving.	0.52	8.53	$p < 0.01$	Supported
H2a:	The higher the perceived usefulness of autonomous driving, the more favorable the attitude toward autonomous driving.	0.32	5.25	$p < 0.01$	Supported
H2b:	The higher the perceived usefulness of autonomous driving, the greater the usage intention of autonomous driving.	−0.02	0.28	n.s.	Not supported
H3a:	The higher the perceived ease of use of autonomous driving, the more favorable the attitude toward autonomous driving.	0.09	2.06	$p < 0.05$	Supported
H3b:	The higher the perceived ease of use of autonomous driving, the greater the perceived usefulness of autonomous driving.	0.02	0.44	n.s.	Not supported
Relationships between social influence and the TAM					
H4a:	Subjective norm positively influences the perceived usefulness of autonomous driving.	0.11	2.64	$p < 0.01$	Supported
H4b:	Subjective norm positively influences attitudes toward using autonomous vehicles.	0.05	1.09	n.s.	Not supported
H4c:	Subjective norm positively influences the intention to use autonomous vehicles.	0.06	1.29	n.s.	Not supported
Relationships between individual differences and the TAM					
H5:	The greater the desire to exert control, the lower the intention to use autonomous vehicles.	−0.01	0.36	n.s.	Not supported
H6:	Privacy concerns associated with autonomous driving negatively influence attitudes toward autonomous driving.	−0.01	0.12	n.s.	Not supported
H7:	The higher the trust in autonomous driving, the greater the intention to use autonomous vehicles.	0.18	3.34	$p < 0.01$	Supported
H8:	The higher the ecological awareness, the greater the perceived usefulness of autonomous driving.	−0.01	0.55	n.s.	Not supported
H9a:	The higher the personal innovativeness regarding autonomous driving, the higher the perceived usefulness of autonomous driving.	0.05	1.67	$p < 0.10$	Supported
H9b:	The higher the personal innovativeness regarding autonomous driving, the higher the perceived ease of use of autonomous driving.	0.17	3.21	$p < 0.01$	Supported
Relationships between system characteristics and the TAM					
H10a:	The higher the perceived relative advantage of autonomous driving, the greater the perceived usefulness of autonomous driving.	0.64	14.59	$p < 0.01$	Supported
H10b:	The higher the perceived relative advantage of autonomous driving, the more positive the attitude toward autonomous driving.	0.33	4.78	$p < 0.01$	Supported
H11a:	The higher the compatibility of autonomous driving with the potential user's mobility needs, the greater the perceived usefulness of autonomous driving.	0.17	4.03	$p < 0.01$	Supported
H11b:	The higher the compatibility of autonomous driving with the potential user's mobility needs, the more favorable the perceived attitude toward autonomous driving.	0.19	3.66	$p < 0.01$	Supported
H11c:	The higher the compatibility of autonomous driving with the potential user's mobility needs, the greater the perceived intention to use autonomous driving.	0.11	2.21	$p < 0.05$	Supported
H12:	The higher the perceived enjoyment of autonomous driving, the greater the perceived ease of use of autonomous driving.	0.39	7.95	$p < 0.01$	Supported
H13:	The higher the (positive) price evaluation, the greater the intention to use autonomous driving.	0.09	2.01	$p < 0.05$	Supported

6. Discussion

6.1. Findings regarding the TAM

In alignment with the findings of Buckley et al. (2018a,b), our study reveals attitude as a statistically significant predictor of the intention to use autonomous driving. This finding is in line with previous research on the attitude–intention relationship, showing that a generally positive attitude leads to an increased likelihood of usage intention and, thus, actual use (Ajzen, 1991; Chin et al., 2008; Davis, 1989). In addition, perceived usefulness and perceived ease of use were found to be positively associated with attitudes toward autonomous driving use.

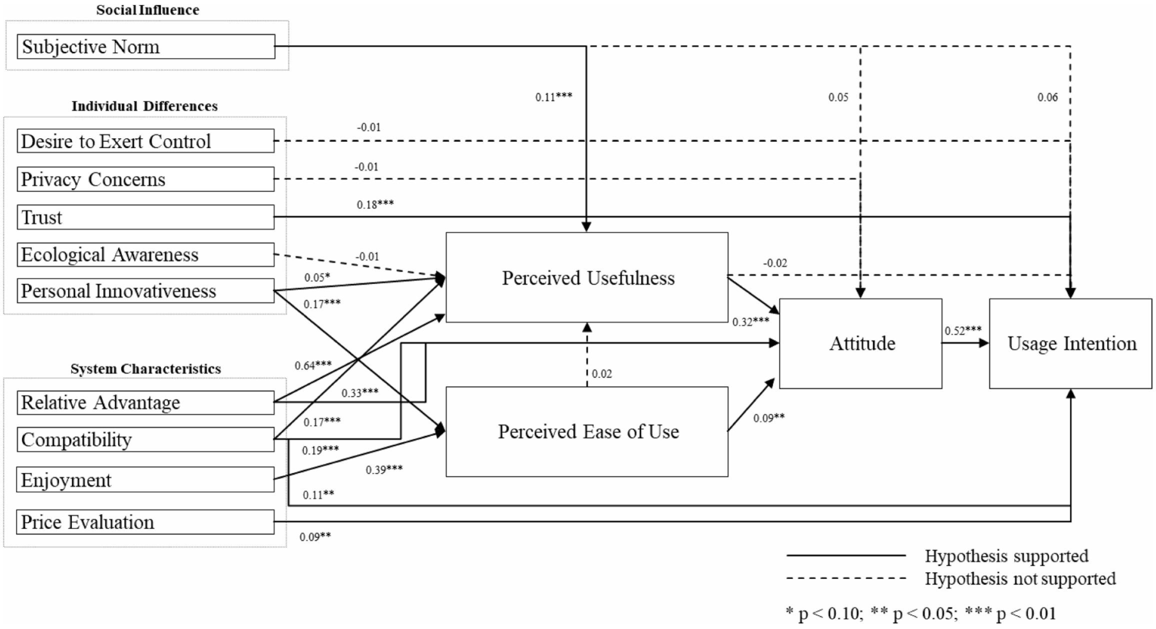

Fig. 3. Results of the structural equation model.

Although previous research has studied the relationship between perceived ease of use, perceived usefulness, and the intention to use autonomous driving (Panagiotopoulos and Dimitrakopoulos, 2018; Wu et al., 2019; Xu et al., 2018), their effect on attitude toward using autonomous driving has not been investigated to date. Both constructs reflect the user's expectation of autonomous driving (Choi and Ji, 2015). These relationships may be best explained by the role of perceived usefulness in improving driving performance and perceived ease of use as a form of perceived effortlessness associated with autonomous driving. Through these results, we demonstrate that attitudes toward using autonomous vehicles (and thus the intention to use autonomous vehicles) mainly depends on how easy and how useful autonomous driving is perceived to be.

6.2. Findings regarding social influence

Our study is the first to reveal a statistically significant prediction of the effect of subjective norm on perceived usefulness, which, in turn, is positively associated with attitude, and, thus, intention to use autonomous driving. Although previous research has revealed a significant effect of subjective norm on the intention to use autonomous driving (Buckley et al., 2018a), we did not find a direct, statistically significant prediction of the effect of subjective norm on attitude and intention to use autonomous driving. Subjective norms influence the perceived usefulness of technological innovations through certain internalization effects, which represent the "tendency to interpret information from important others as evidence about reality" (Schepers and Wetzels, 2007, p. 91). In particular, inexperienced individuals are likely to be influenced by the opinion of third parties in the formation of acceptance (Venkatesh and Davis, 2000).

6.3. Findings regarding individual differences

First, our findings reveal a statistically significant effect of trust on the intention to use autonomous driving. These results correlate with a previous study conducted by Choi and Ji (2015) suggest that system transparency, which refers to the degree of comprehensibility and predictability of the operations of autonomous vehicles, is positively associated with trust in autonomous driving. Accurate autonomous technology can improve the user's experience by helping them understand the operation of autonomous vehicles. The positive relationship between technology-related trust and intention to use has also been confirmed by other studies (e.g., Buckley et al., 2018a; Hengstler et al., 2016; Hohenberger et al., 2017).

Second, it is found that personal innovativeness positively affects perceived ease of use and perceived usefulness. These relationships have not been investigated by prior autonomous driving research. However, these findings are consistent with prior research on other technological innovations (e.g., Kuo and Yen, 2009; Lin et al., 2007; Mao et al., 2005). Individuals who would like to try and adopt autonomous vehicles earlier than other people seem to perceive autonomous driving as easier to use and more useful. We argue that innovative individuals perceive autonomous driving as more likely to be useful and easy to use because innovators are generally more ready to adapt to changes (Hurt et al., 1977). In addition, innovators actively seek information about new ideas (Rogers, 2003) and therefore are likely to form positive perceptions about the usefulness and ease of use of new innovations such as autonomous driving. Thus, innovators are also more likely to accept autonomous driving, especially when they have already experienced newer technologies in the automotive context (Dikmen and Burns, 2016).

Finally, the proposed effects of desire to exert control on intention, ecological awareness on perceived usefulness, and privacy concerns on attitude are not fully supported by this study and thus warrant further investigation. Nevertheless, given the findings of our qualitative study, we argue that the identified factors can influence an individual's acceptance. In addition, previous literature has stressed the importance of these constructs when assessing acceptance related to autonomous driving (Buckley et al., 2018a; Fraedrich and Lenz, 2016; Wu et al., 2019). Giving up control of the driving task to autonomous vehicles might lead to a certain degree of anxiety, which, in turn, reduces the overall acceptance of autonomous driving (Koo et al., 2016). Venkatesh (2000) conceptualizes anxiety in the context of technology acceptance as a certain emotional apprehensiveness related to the possibility of using a technology. The resulting anxiety about giving up control to autonomous vehicles can negatively impact the intention to use autonomous vehicles, due to the expected negative affective associations. With respect to the influence of ecological awareness on perceived usefulness, research has indicated that individuals interpret innovations providing environmental advantages differently from innovations that, for example, enable economic benefits (Melville, 2010). Since autonomous driving is expected to substantially contribute to solving environmental problems (Anderson et al., 2016; Brown et al., 2014), individuals with high ecological awareness perceive autonomous vehicles as more useful compared to individuals with a generally low tendency to behave ecologically correctly. This is in line with the recent research findings of Wu et al. (2019) who reveal a significant effect of environmental concerns on perceived usefulness of autonomous driving. Finally, privacy concerns related to the access and storage of an individual's movement data have been noted as highly concerning by potential users of autonomous vehicles, thus lowering the general interest in using autonomous vehicles (Fraedrich and Lenz, 2016). In particular, revealing information about the location of vehicles—which is inevitable for the success of autonomous driving—is seen as problematic by individuals (Mahoney and O'Sullivan, 2013). These concerns are mainly related to worries about processing personal information and the resulting invasion of personal space (Solove, 2006). In addition, autonomous vehicles are believed to be prone to hacks, which raises new concerns about data safety (Douma and Palodichuk, 2012). Hence, we argue that such privacy concerns might negatively influence willingness to use autonomous vehicles.

6.4. Findings regarding system characteristics

The study reveals that compatibility is a relevant predictor of the attitude toward and intention to use autonomous driving, as well as its perceived usefulness. Although research has emphasized the importance of considering compatibility in the acceptance-building process (e.g., Karahanna et al., 2006; Teo and Pok, 2003; Tornatzky and Klein, 1982), it has not been thoroughly studied in the context of autonomous driving. We argue that a higher perceived compatibility of autonomous vehicles with current mobility behavior results in lower perceived disruption and magnitude of change, which, in turn, reduces reluctance to use autonomous driving and increases acceptance.

In addition, the study identifies the relative advantages as positively associated with both perceived usefulness and attitude toward using autonomous driving. Previous research has emphasized the importance of considering relative advantage in the acceptance process (e.g., Kulviwat et al., 2007; Lin, 2011). However, insights into the relationship between relative advantage and acceptance in the context of autonomous driving is limited and requires further attention (Lee et al., 2019). Although relative advantage is considered similar to perceived usefulness, research has distinguished between the two constructs, as relative advantage refers to the usage of a technology in comparison to the idea it supersedes, rather than the actual usefulness of a current technology (Carter and Bélanger, 2005). Our findings, such as the significant relationship between relative advantage and attitude, extend the research of Lee et al. (2019), who could not report a significant effect between relative advantage and willingness to use autonomous vehicles.

In addition, the present study shows that perceived enjoyment

positively influences the perceived ease of use of autonomous driving. Although previous research has found that a low degree of automation, that is, manual driving, is perceived as more enjoyable compared to automated driving (Kyriakidis et al., 2015), perceived enjoyment has not been embedded in an acceptance model for autonomous driving. In alignment with Venkatesh (2000), we argue that perceived enjoyment increases the intrinsic motivation to use autonomous driving, and thus makes interaction with autonomous vehicles easier.

Finally, it is found that price evaluation has a beneficial association with intention to use autonomous driving. Autonomous vehicles need to be equipped with special, costly sensors, wireless networks, smart navigation systems, automated controls, as well as servers, software, and power supplies with high reliability standards, thus exceeding the price of current vehicles. The light-detection and ranging (LIDAR) system used in Google's self-driving vehicles costs around US$70,000 (Varghese and Boone, 2015). Currently, additional costs of up to US $50,000 per vehicle are expected (Merfeld et al., 2019b). Research indicates that there is a willing market for autonomous driving when the prices of those vehicles are close to those of conventional vehicles (Kyriakidis et al., 2015). Costs of autonomous driving are expected to fall rapidly with mass production (Fagnant and Kockelman, 2015). The resulting cost reductions will improve the perceived cost–benefit ratio, thus positively influencing the intention to use autonomous driving.

7. Overall contributions

7.1. Implications for research

First, our study overcomes a major limitation of prior studies, as pointed out by Gkartzonikas and Gkritza (2019): “studies have investigated the likelihood that AVs [autonomous vehicles] will be adopted and the process by which that might happen, the questions included in those studies’ respective surveys have not been based on well-established theories” (p. 335). Although such studies (e.g., Brell et al., 2019; Hudson et al., 2019; König and Neumayr, 2017) are valuable and useful for generating wide-ranging insight into public opinion about and attitude toward autonomous driving, they do not offer a detailed explanation of how the derived factors are interrelated and inform user acceptance. Our study thus extends these findings by relying on the well-established TAM to capture a set of factors that determine the acceptance of autonomous driving.

Second, we combine the advantages of qualitative and quantitative research methods to shed light on the acceptance of autonomous driving and thus address the following research gap. A majority of previous studies have investigated the acceptance of autonomous driving utilizing a quantitative research method (see Appendix A), while insights regarding the thoughts and psychological causes that lie behind the respective acceptance factors are still limited and require further attention, particularly in the context of autonomous driving (Buckley et al., 2018b; Merfeld et al., 2019a; Nordhoff et al., 2019). In addition, the majority of TAM studies focus on a limited, study-specific set of factors to understand the process of adopting autonomous driving (e.g., Anania et al., 2018; Hulse et al., 2018; Wu et al., 2019). In this regard, Wu (2012) points out that the majority of TAM studies adhere to the following path of empirical investigation: “review previous literature → select relevant factors such as PU [perceived usefulness] and PEOU [perceived ease of use] for the study → propose hypotheses/ model → collect empirical data from a quantitative survey → test the hypotheses and validate the model” (p. 174). Through our qualitative study, we have disclosed perceptions of autonomous driving from an end-user point of view and explored their relationships by means of a survey. By utilizing a mixed-method approach, our study identifies constructs that have not been captured by prior quantitatively conducted studies. Our study does, for example, reveal perceived enjoyment related to autonomous driving to be a statistically significant predictor of perceived ease of use, which, in turn, positively influences user acceptance. We thus extend existing literature (e.g., Haboucha et al., 2017; Hegener et al., 2019), which takes a different approach to conceptualizing perceived enjoyment, that is, the enjoyment of driving a car. In addition, the qualitative study identifies the desire to exert control as a potential factor predicting the acceptance of autonomous driving. Although our study could not confirm a statistically significant influence of desire to exert control on behavioral intentions, the interviews revealed that users tend to consider the degree to which they have a demand for control in connection with autonomous driving in their acceptance formation. Therefore, our study opens new avenues for further research, as prior studies have focused on the desire for control as a general personality trait (e.g., Choi and Ji, 2015). In addition, price evaluation has been investigated by previous research in terms of an individual's willingness to pay for autonomous driving (Bansal und Kockelman, 2017; Daziano et al., 2017; Talebian and Mishra, 2018). To the best of our knowledge, the impact of price evaluation conceptualized as the degree to which individuals consider the cost–benefit ratio in their usage decision, has not been investigated in detail. Our study shows that the perceived cost–benefit comparison significantly affects the intention to use autonomous vehicles. Finally, while previous research has focused on compatibility of autonomous driving related to transportation infrastructure (e.g., Rahman et al., 2019), our study takes a different approach by conceptualizing compatibility as the extent to which autonomous driving fits into the individual's mobility behavior.

Third, while some studies have revealed relevant relationships between psychological factors and willingness to use autonomous driving, Lee et al. (2019) and Xu et al. (2018) state that prior studies reveal incongruent and conflicting findings on the factors that influence acceptance formation of autonomous driving. Our research contributes to these shortcomings by providing new insights into the relationships between the psychological factors and their impact on willingness to use autonomous driving, as well as providing evidence for previously unsupported hypotheses. For example, our findings do not support a statistically significant relationship between privacy concerns and attitudes toward using autonomous vehicles and thus support previous research results (Zhang et al., 2019). In addition, our study reveals a statistically significant impact of trust on behavioral intentions and, thus, substantiates previous findings in the literature (e.g., Benleulmi and Becker, 2017; Choi und Ji, 2015; Panagiotopoulos and Dimitrakopoulos, 2018). Contradicting earlier findings (e.g., Wu et al., 2019), we could not validate that ecological awareness statistically predicts perceived usefulness. Nevertheless, it should be noted that Wu et al. (2019) analyze autonomous electric vehicles and conceptualize their perceived usefulness as the extent to which users believe that using autonomous vehicles will increase the environmental performance of their lives. Further research is required to confirm the impact of ecological awareness on acceptance formation because the usage context (shared or privately owned) and cultural differences might influence this relationship (Haboucha et al., 2017). Our study extends Liu et al.’s (2018) findings, which conceptualize willingness to pay as a final outcome variable of autonomous vehicle user acceptance but neglects to consider the relationship between willingness to pay and behavioral intentions. In addition, while Panagiotopoulos and Dimitrakopoulos (2018) reveal a statistically significant effect of social norm on behavioral intentions, we extend these findings by showing that social norm also predicts perceived usefulness. This finding is also confirmed by Zhang et al. (2020). Benleulmi and Becker (2017) and Hegner et al. (2019) show that personal innovativeness is positively associated with behavioral intentions toward using autonomous driving. Our study therefore extends and provides additional insights into these findings by revealing that personal innovativeness can also predict the perceived ease of use and perceived usefulness of autonomous driving.

Finally, we follow Benbasat and Barki's (2007) call and move closer to behavioral research by integrating the attitude construct as a

predictor for understanding behavioral intentions and behavior. The attitude construct is a key factor when investigating the acceptance of technologies (Venkatesh et al., 2003), particularly in the consumer context (e.g., Djamasbi et al., 2009; Furneaux and Nevo, 2008; Kulviwat et al., 2007). By including the attitude construct in our study, we contribute to the findings of existing studies that have focused on behavioral intentions to predict acceptance of autonomous driving (e.g., Choi and Ji, 2015; Hegner et al., 2019; Kaur and Rampersad, 2018). The attitude construct reflects the affective and cognitive perception related to an object and is a powerful influencing factor in predicting use of technological innovations (Yang and Yoo, 2004). Our findings underline the importance of integrating the attitude construct in the investigation of autonomous vehicle acceptance. For example, while Lee et al. (2019) do not show a statistically significant relationship between relative advantage and behavioral intentions, our study sheds new light on the relationship between relative advantage and user acceptance by revealing that relative advantage is positively associated with attitude toward using autonomous driving.

7.2. Implications for practice

Our study also identifies implications for practice. First, autonomous driving provides several benefits to individuals and society, including more accessible mobility, traffic flow optimization, greater safety precautions on the roads, and an improved ecological footprint. Benefits such as these have the ability to increase the perception of the usefulness of autonomous driving and therefore lead to increased user acceptance. However, individuals remain concerned about the use of autonomous driving technology (Kyriakidis et al., 2015), as it can, for example, increase total vehicle travel and thus emissions, reduce security and comfort, or increase congestion (e.g., Begg, 2014; Brown et al., 2014; Haboucha et al., 2017). Since there are both advantages and disadvantages associated with autonomous driving, we argue that it is important to design autonomous driving systems so that benefits are realized and disadvantages are minimized. Environmental benefits, for example, are more likely to be achieved when supporting shared vehicle services and self-driving buses, which, in turn, are also expected to reduce vehicle ownership (Brendel et al., 2018; Greenblatt and Shaheen, 2015; Merfeld et al., 2019b). This is particularly important considering the trend toward decreased personal ownership of vehicles and the rise of on-demand and shared transport services (e.g., Dacko and Spalteholz, 2014). In this regard, policymakers and operators of fleets should consider current mobility preferences of users in terms of daily driving practice or traveling. When potential end-users are convinced that autonomous driving fits their mobility behavior and preferences, they will judge autonomous driving as more useful, which in turn might lead to higher acceptance. Policymakers could, for example, set price incentives for car-sharing operators or car-riding concepts as a supplement and extension to public transport. Another advantage to be considered by policymakers is that autonomous vehicles in the field of mobility on demand solve the problem of the first and last mile (lack of proper connections between transport modes) and thus also significantly improve public transport, making it more accessible and attractive (Greenblatt and Shaheen, 2015). Depending on the scenario, autonomous vehicle fleets could even completely replace public passenger transport, especially when mobility concepts such as ridesharing cover many areas (Meyer et al., 2017). However, such scenarios are considered unrealistic, particularly in rural areas, in which autonomous driving is unlikely to replace private vehicle use. We argue that policymakers should, in cooperation with operators of fleets and research institutions, subsidize investigations in new, sustainable mobility, end-to-end solution concepts with regard to autonomous vehicles, particularly in rural areas. In addition, policymakers should incentivize the use of sharing mobility options with autonomous vehicles in less populated areas.

Further, increased safety is more likely to be realized when coordination is improved through vehicle-to-vehicle and vehicle-to-infrastructure communications, or autonomous vehicles can use existing (or additional) lanes (Fagnant and Kockelman, 2015). O'Toole (2014), for example, recommends that policymakers focus on improving existing infrastructure, such as dynamic traffic signal coordination, to reduce safety and congestion problems. However, such investments dramatically increase the total costs of autonomous driving (Fagnant and Kockelman, 2015) and thus might negatively impact the perceived cost–benefit perception for users. Policymakers should therefore ensure that such investment costs are not transferred to the end-user through, for example, extensive road usage fees. In addition, current autonomous vehicle technology costs are estimated to be approximately $100,000 or more and will thus not provide significant monetary benefits from savings in fuel, parking, or insurance (Fagnant and Kockelman, 2015). Mass production will drastically lower these costs. Fagnant and Kockelman (2015) predict that at an additional cost of $10,000 for an autonomous vehicle, it becomes a realistic investment for users. Mass-market penetration of autonomous driving is also expected to reduce substantially social costs related to congestion, traffic accidents, and air pollution (Anderson et al., 2016). However, the actual benefits and costs are still not well known and will depend greatly on the operational mode of autonomous driving and further circumstances, such as usage context (e.g., rural or urban), ownership structure, or demand (for a detailed discussion see Boesch et al., 2018). For example, the expected benefits related to traffic and emissions can be offset considering that autonomous driving enables a greater vehicle throughput on existing roads, increased travel speed, or enabling underserved groups to travel (Brown et al., 2014). Therefore, more research and tests are needed to validate the estimated private and public costs and benefits to assist policymakers in the decision as to whether and how to subsidize autonomous driving. Autonomous vehicles will, for example, be cheaper to operate for car-sharing operators because the costs of compensating drivers can be excluded, which, in turn, may discourage vehicle ownership (Bagloee et al., 2016). In this regard, Fagnant and Kockelman (2018) suggest that a fleet operator could generate a 19% return on investment considering a purchase price of $70,000 and travel fares of $1 per trip-mile.

In addition, policymakers and manufacturers can also act as a kind of expert, thereby strongly shaping the positive public perception of the usefulness of autonomous vehicles. They should, therefore, clearly highlight and communicate the advantages of autonomous over conventional vehicles. Thorpe and Motwani (2017) suggest different communication strategies to increase the success of autonomous driving. Benefits should be presented by using simple “Did you know?” statements that appeal to people's feelings of regret over not using autonomous vehicles (e.g., “Did you know that everyday more Australians like you are using autonomous vehicles and saving X days of commuting each year?”). The communication strategies should also be extended to social networking sites, because negative messages regarding risks are frequently expressed on such platforms (Kohl et al., 2017). In this regard, Hegner et al. (2019) posit that in the era of digital marketing, influencers can act as key opinion leaders and thus, manufacturers and fleet operators should cooperate with them to increase awareness about the benefits of autonomous vehicles as well as strengthen brand image. The Mercedes VIP leasing program is a good example of such an initiative; the company collaborates with influencers by offering cheaper leasing contracts to strengthen its brand image among young people.

Strategies should also aim at increasing trust in autonomous driving. Defining standards regarding self-driving maturity, with an independent, official quality label approved by policymakers, could increase the perceived brand image of autonomous vehicle suppliers and, therefore, be useful to support the formation of trust in this technology. Anderson et al. (2016) suggest that autonomous vehicles should only be made available to the public once they have been issued a specific safety certificate by manufacturers or authorized autonomous

technology certification facilities. In addition, research has shown that transparency about a technology's functionality can create a solid basis for trust in the technology (Hoff and Bashir, 2015). Policymakers and manufacturers could, therefore, provide the public with a transparent knowledge base on the functionality of autonomous driving, which is easily accessible and available to the broad public. Since test drives could also help to establish or strengthen confidence in autonomous driving, policymakers should cooperate with manufacturers and operators of fleets to support supervised test zones outside real-traffic areas. This would have the advantage of potential users experiencing autonomous driving technology without having concerns that other traffic participants or the test driver are in danger. In addition, such test zones could help to overcome trust barriers, as there is evidence that the first experience in a self-driving car is associated with a feeling of uneasiness due to the shift of control from the driver to the vehicle (Nunez, 2017).

Test drives and pilot studies should particularly be aimed at individuals with high levels of personal innovativeness. As these populations generally perceive the interaction with autonomous vehicles as easy and useful, their attitude and, thus, intention toward such technologies, is greater. Hence, these individuals can act as a reference group, having a positive social influence on other groups. Therefore, especially for countries with high barriers to autonomous driving, we suggest establishing policies that promote test drives and pilot studies with individuals who have high levels of personal innovativeness. This is especially important because policy efforts in promoting autonomous driving vary significantly between countries. While U.S. lawmakers tend to remove barriers for testing and field operations, European legal and administrative processes have tended to hamper the broad rollout of autonomous vehicles (Patti, 2019; Pattinson and Chen, 2020). While, for example, the testing of autonomous vehicles on public roads is still limited in most European countries, it is already permitted in California and will be further promoted in the future with the help of new laws. Authorities need to decide which legal instruments, that is, binding regulations, non-binding regulations, or exemptions under conditions, are suitable to accommodate and test self-driving cars (Vellinga, 2017). Vellinga (2017) further emphasizes that the legal instrument must have the ability to keep pace with technological developments, while also ensuring legal certainty for manufacturers, test organizations, and users. However, not only national law but also international laws and regulations need to be reconsidered. The Vienna Convention on Road Traffic 1968 and the Geneva Convention on Road Traffic 1949 greatly influence traffic laws worldwide. However, both conventions pose obstacles to the implementation of autonomous driving because they require a driver for moving vehicles, who is in control of the vehicle (see Vellinga, 2017 for a discussion on the impact of these conventions on autonomous driving).

Thus, clear and strong security standards must be defined by policymakers, as hacking or terrorism in the context of autonomous driving constitutes a threat to societal security. Such standards can enhance the trustworthiness of autonomous driving in general and thus lead to increased acceptance. Additionally, policymakers, manufacturers, and operators of fleets need to address issues relating to the access and storage of an individual's mobility data, clarifying which kind of information autonomous vehicles are allowed to gather and where, and for how long this information can be stored. Although it is technically possible to limit the generalization and distribution of personal data in autonomous driving, a variety of stakeholders are interested in using these data; for example, insurers, car manufacturers, or government authorities (Anderson et al., 2016). Policymakers need to develop a solid concept that reflects the interests of both the user and other parties. Anderson et al. (2016) state that policymakers need to clarify questions such as whether automakers are allowed to sell data to marketers or insurers, or whether the data should be discoverable in legal proceedings. Glancy (2012) summarizes that three types of privacy interests—personal autonomy, personal information, and surveillance—will become central in the discussion of legal issues. Although data protection directives, such as the General Data Protection Regulation, regulate the processing of personal data and require that the user gives consent to the processing of personal data, such regulations often fail to address exploitative contracting or improvident users (Hacker, 2017). To make such directives more applicable to the context of autonomous driving, policymakers could, for example, set up regulations (with at least two options) that autonomous vehicle service providers are legally required to present to the user. In the first option, the user might consent to personal data being collected and processed (the user can be compensated by a lower price for buying or using self-driving cars), while in the second option, the user's personal data might not be collected, and the service provider may be compensated by a higher price (Hacker, 2017).

However, a focus on performance improvement is not, by itself, sufficient for successful market penetration; the enjoyment factor should not be neglected by manufacturers, as strict regulations could also reduce the pleasure of using autonomous driving. Policymakers should subsidize relevant research to investigate such aspects in more detail.

8. Limitations and future research

There are some limitations with respect to the generalization of the results. First, the paper is based on the assumption that autonomous vehicles will be at SAE level 5. It does not consider whether there will be a gradual introduction of different levels of automation with different characteristics.

Another limitation of this study is that participants were not given a specific usage context for autonomous driving. The aim of our research was to explore the acceptance of autonomous vehicles from a general point of view. However, research has shown that the usage context, such as private ownership, leasing, or shared autonomous vehicles and rides, leads to different costs and benefits to drivers, and thus the factors that shape the user's acceptance might differ (Acheampong and Cugurullo, 2019; Lee et al., 2019). Future research should, therefore, investigate users' acceptance based on different usage contexts.

Third, albeit the TAM has been suitably shown to predict future technologies, it should be stated that the results of the model may still be skewed, ultimately due to the lack of direct experience (Nordhoff et al., 2016). Although the provided video and definition of autonomous driving might help participants to put themselves mentally into the mindset of a driver, it cannot substitute for real driving experience with autonomous vehicles and stated preferences and actions in a hypothetical, imagined situation do not always match actual preferences and actions (Brandts and Charness, 2000). Also, Rahman et al. (2017) note that acceptance of autonomous systems increases when experience with them also increases. For that reason, our present findings should be interpreted carefully, since the results rely to a large extent on the individual's imagination of autonomous driving. We recommend reproducing this study after autonomous driving has been established, thereby including experienced individuals.

As autonomous driving enables access to mobility for underserved groups, for example, the elderly, individuals without a driver's license, or physically impaired people, these populations should be included in further research. Furthermore, future research could investigate whether sociodemographic factors like age, gender, or living conditions moderate the investigated relationships.

Since the paper's focus is on individual acceptance, future research should also investigate social acceptance (see Nordhoff et al., 2016), that is, the reaction of the public to the introduction of a traffic system with autonomous vehicles.

Finally, it should be noted that the study was conducted in a European country setting, that is, Germany. The generalizability of this study is therefore limited to sample- and country-specific characteristics. Future research, therefore, should duplicate this research in other

geographical areas to determine the generalizability of the findings.

9. Conclusion

Based on a qualitative research design, the present study has developed a research model that was validated with an online-based survey. The results indicate that three main factors—social influence, individual differences, and system characteristics—are likely to influence the acceptance of autonomous driving. With regard to our research question—what drives the acceptance of autonomous driving from an end-user perspective?—we investigate 14 acceptance factors (i.e., subjective norm, desire to exert control, privacy concerns, trust, ecological awareness, personal innovativeness, relative advantage, compatibility, enjoyment, price evaluation, perceived usefulness, perceived ease of use, attitude, and usage intention) and examine their relationships using structural equation modeling. Our results show that subjective norm can be a significant factor that affects perceived usefulness, while trust is positively related to usage intentions. In addition, the findings show that personal innovativeness predicts perceived usefulness and perceived ease of use, which in turn, positively affects attitude toward using autonomous driving, a significant predictor of usage intentions. Further, relative advantage and compatibility positively influence attitude toward using autonomous vehicles as well as perceived usefulness. In addition, compatibility positively affects usage intentions. Finally, our results confirm a positive relationship between enjoyment and perceived ease of use and a positive relationship between price evaluation and usage intentions. Thus, our study contributes to existing research by deriving relevant acceptance factors that have not yet been extensively examined in previous research. In addition, our study reveals novel relationships between factors that shape the formation of acceptance and shed lights on incongruent and conflicting findings. The study also brings new aspects into the discussion for practitioners. For example, on an individual level, practitioners need to ensure that the implemented technology is trustworthy from an end-user's point of view because this, in turn, has a lasting effect on acceptance. The developed service based on autonomous driving should also fit current mobility patterns as compatibility remains a relevant factor as a system characteristic. On a social level, practitioners should ensure a positive response toward the usefulness of autonomous driving, as this might influence people's own perceptions of the utility of autonomous driving. Practitioners should take up the factors presented and, for example, incorporate and consider adapting them into their communication strategies and service development, thus reducing the time to market. In conclusion, our study reveals that although autonomous driving offers tremendous advantages for individuals and society, several user-related aspects need to be addressed before this technological innovation is ready to enter the mass market.

CRediT authorship contribution statement

Ilja Nastjuk: Conceptualization, Methodology, Formal analysis, Investigation, Writing - original draft, Writing - review & editing, Visualization. **Bernd Herrenkind:** Conceptualization, Methodology, Formal analysis, Investigation, Writing - original draft, Writing - review & editing, Visualization. **Mauricio Marrone:** Supervision, Conceptualization. **Alfred Benedikt Brendel:** Supervision, Conceptualization. **Lutz M. Kolbe:** Supervision, Conceptualization.

Declaration of Competing Interest

None.

Appendices

Appendix A

References	Study objective(s)	Research context/ Focus	Method(s)	Theoretical framework(s)	Investigated constructs	Derivation of extensions	Main finding(s)
Acheampong and Cugurull-(2019)	Understanding and predicting autonomous vehicle interest and adoption intentions as well as user adoption decisions regarding three different autonomous vehicle modes	Autonomous vehicles in context of ownership, sharing, and public transport (NHTSA level 4)	Confirmatory factor analysis	Theory of planned behavior; socio-ecological model of behavior; technology acceptance model; technology diffusion model; perceived characteristics of innovating belief	Model 1: PEOU, PB, fears and anxieties, SN, image, ATT toward technology, PBC, sociodemographics, adoption intention<br>Model 2: Adding ATT toward environment, ATT toward collaborative consumption<br>Model 3: Adding ATT toward public transit<br>Model 4: Adding ATT toward car ownership	Theory- and literature-based	• Public fears, anxieties → ATT (+)<br>• Public fears, anxieties → PB (+)<br>• SN → PB, PBC (+)<br>• Environmental ATT, CC ATT, and technology ATT correlate positively with each other<br>• PB → environmental and technology ATT (+)<br>• PB positively correlate with pro-environmental and pro-technology ATT while negative technology ATT correlates negatively with PB and pro-environmental ATT<br>• Environmental ATT → public transit ATT (+), car ownership (-)
Anania et al. (2018)	Investigating different types of information (positive or negative) on consumer perceptions regarding autonomous vehicles as well as moderating effects regarding socio-demographic factors	Consumer perceptions and willingness to ride autonomous vehicles (NHTSA levels 3–4)	Descriptive statistics (reliability analysis; inferential analysis)	–	Willingness to ride depending on received (positive/ negative) information	–	• Individuals are more willing to ride in autonomous vehicles after hearing positive information about them and vice versa<br>• Differences in terms of nationality were found. There were no consistent differences in terms of gender
					–	–	

Bansal and Kockelman (2017)	Proposing a new simulation-based fleet evolution framework to forecast Americans' long-term adoption of connected autonomous vehicles with different technology levels (depending on certain scenarios)	Americans' long-term adoption of connected autonomous vehicles (NHTSA level 1–4)	Descriptive analysis; simulation	Simulation-based fleet evolution framework			• Insights into Americans' adoption of autonomous vehicles based on willingness to pay. Framework to forecast the adoption under different scenarios regarding price, willingness to pay, and regulations
Benleulmi and Becker (2017)	Investigating drivers and inhibitors of autonomous cars' acceptance across cultures	Acceptance of autonomous cars with a special focus on the different risks deterring consumers from Germany and the U.S. (SAE levels 4 and 5)	Structural equation modeling	Unified theory of acceptance and use of technology	Performance expectancy, effort expectancy, social influence, hedonic motivation, BI; extension: desirability of control, perceived convenience, personal innovativeness in IT, intention to prefer autonomous over conventional car, privacy risk, performance risk, financial risk, socio-psychological risk safety, trust, disposition to trust autonomous car	Literature-based	• Trust, intention to prefer autonomous car, perceived convenience, personal innovativeness in information technology, hedonic motivation, effort expectancy, and social influence → BI (+)• Desirability of control → BI (-)<br>• *Perceived convenience, performance expectancy → BI*<br>• Performance risk, safety, disposition to trust autonomous car → trust (-)<br>• Socio-psychological risk → trust (+)<br>• *Financial and private risk → trust*
Bernhard et al. (2020)	Exploring crucial determinants for the use of an autonomous minibus	Autonomous minibus in Germany	Descriptive analysis, multiple linear regression	Unified theory of acceptance and use of technology	Performance expectancy, effort expectancy, questionnaire type (experience), BI to use autonomous public transport, willingness-to- use the autonomous minibus<br>Additional post-ride: perceived safety, valence, minibus characteristics (need for an operator, spaciousness, velocity, braking), environmental friendliness	Literature-based	• Average rating is positive, post-ride slightly higher<br>• Performance expectancy, effort expectancy → BI (+)<br>• Age, Experience → BI (-)<br>• Gender → BI (-)<br>• Valence, spaciousness, performance expectancy → willingness-to-use the autonomous minibus (+)<br>• Need for an operator, velocity, effort expectancy, gender → willingness-to-use the autonomous minibus (+)<br>• Age, braking → willingness-to-use the autonomous minibus (-)
Buckley et al. (2018a)	Investigating the relevance of psychological constructs regarding intention to use automated vehicles	Intention to use conditional automated vehicles in the US (SAE level 3)	Descriptive analysis; bivariate correlations; hierarchical regression analysis	Theory of planned behavior; technology acceptance model	ATT, SN, PBC, PU, PEOU, BI; extension: trust	Theory- and literature-based	• Theory of planned behavior constructs as well as PEOU significantly influence the BI of automated vehicles<br>• Trust contributed variance to both models
Cho et al. (2017)	Deriving factors that affect the user's experience and acceptance of autonomous vehicles as well as investigating changes dependent on the level of automation	Behavioral intention based on user experience assessment with a driving simulator in Korea (NHTSA levels 1–4)	Linear regression	Car technology acceptance model, unified theory of acceptance and use of technology	Performance expectancy, effort expectancy, social influence, BI, self-efficacy, anxiety, perceived safety; extension: trust, affective satisfaction, level of automation	Theory- and literature-based	• Performance expectancy, social influence, trust, affective satisfaction, and perceived safety → BI (+)<br>• Anxiety → BI (-)<br>• *Effort expectancy, self-efficacy → BI*<br>• Social influence, perceived safety, trust, and affective satisfaction increase up to 2nd level of automation, decrease to minimum in 3rd level, and increase again in 4th level, anxiety behaves vice versa (due to increase in experience)
Choi and Ji (2015)	Investigating the user's adoption aspects of autonomous vehicles as well as examining the factors that drive people to trust an autonomous vehicle	Adoption of autonomous vehicles at an early stage in Korea (NHTSA levels 3 and 4)	Structural equation modeling	Technology acceptance model; trust theory	PU, PEOU, BI; extensions: trust; perceived risk, system transparency, technical competence, situation management, locus of control, sensation-seeking	Theory- and literature-based	• System transparency, technical competence and situation → trust (+)<br>• Trust → PU, BI (+)<br>• Trust → perceived risk (-)<br>• Locus of control, PEOU → BI (+)<br>• *Sensation seeking, perceived risk → BI*

Daziano et al. (2017)	Investigating semi-parametric estimates of willingness to pay for automation	Willingness to pay for different automation levels (NHTSA aggregated levels)	Discrete choice experimental methodologies; mixed logit model	Vehicle-purchase discrete choice models	–	–	• Quantification of how households currently perceive and value automated vehicle technologies • Derivation of the willingness to pay for several levels of automation
Haboucha et al. (2017)	Investigating individual motivations for choosing to own and use autonomous vehicles and develop a model for autonomous vehicle long-term choice decision	Sharing or owning of autonomous vehicles from a users' perspective in Israel and North America (NHTSA level 4)	Confirmatory factor analysis; logit kernel model	Model for autonomous vehicle long-term choice decisions	Pro-autonomous vehicle sentiments, environmental concerns, technology interests, public transit ATT, driving enjoyment	Literature-based	• Significance of the following factors: driving enjoyment, environmental concerns and pro-autonomous vehicle sentiments • Israelis more willing to accept autonomous vehicles
Hegner et al. (2019)	Investigating the influence of trust in the technology, concerns in giving up control, perceived usefulness, PEOU, personality factor innovativeness, and enjoyment of driving a car on the a priori intention to adopt an autonomous vehicle	Customer's intention and willingness to adopt autonomous vehicles (SAE level 5)	Structural equation modeling	Technology acceptance model	PU, PEOU, BI; extensions: concern giving up control, trust in technology, driving enjoyment, personal innovativeness	Literature-based	• PU, trust/control concerns, personal innovativeness → BI (+) • Driving enjoyment → BI (-) • *PEOU, gender, age, education → BI* • PEOU → PU, trust/control (+) • PU → trust/control (+)
Howard and Dai (2014)	Investigating people's attitudes toward autonomous vehicles	Case study in Berkeley, California (NHTSA level 4)	Descriptive analysis	–	–	–	• Individuals are most attracted to potential safety benefits, the convenience of not having to find parking, and amenities such as multitasking while on route
Hudson et al. (2019)	Analyzing people's attitudes toward autonomous vehicles	People's attitudes toward autonomous vehicles in Europe using Eurobarameter data relating to Nov/Dec 2014 (no level assignment to SAE or NHTSA)	Regression analysis, factor analysis	–	People's acceptance linked to ATT, socioeconomic and demographic variables reflecting their self-interest, gender, age, education, location, personal prosperity	Literature-based	• People are more uncomfortable with autonomous vehicles with strong variations • Young more in favor than the elderly; old, unemployed, less educated women tend to be less in favor of autonomous vehicles; large towns and cities more in favor • Higher accident rates within countries may lead to a higher support for autonomous vehicles
Hulse et al. (2018)	Investigating percept-ions of autonomous cars (particularly road safety and acceptance) and comparing them in relation to road usage, risk-taking, risk perception, age, and gender	Relationships between road usage (e.g., pedestrian), risk, gender, and age compared to autonomous vehicle acceptance (no level assignment to SAE or NHTSA)	Factor analysis, multinomial logistic	–	Road user risk-taking, perceived risk, general ATT	Literature-based	• Autonomous vehicles seen as a low-risk form of transport with just a few concerns compared to other transportation modes • Gender, age, and risk-taking had varied relationships with different vehicle types and general ATT toward autonomous vehicles (e.g., passengers rate autonomous vehicles riskier than pedestrians), low objections for autonomous vehicles being used on public roads, and little opposition against autonomous vehicles in terms of ATT
Kaur and Rampersad (2018)	Investigating the key factors influencing the adoption of driverless cars	Driverless cars as a public transportation service in closed environments in Australia (SAE level 5)	Confirmatory factor analysis	Unified theory of acceptance and use of technology	Performance expectancy, reliability; extensions: security, privacy, trust, adoption scenarios	Literature-based	• Performance expectancy, trust → BI (+) • Reliability, security, privacy → trust (+)
Koul and Eydgahi (2018)	Investigating antecedents of driverless car technology adoption	Acceptance of driverless car technology (no level assignment to SAE or NHTSA)	Pearson's correlation; multiple linear regression	Technology acceptance model	PU, PEOU, BI driverless cars; extensions: years of driving experience, age; moderators: gender, level of education, household income	Literature-based	• PU, PEOU → BI (+) • Years of driving experience, age → BI (-)

Lee et al. (2019)	Investigating influential factors on intention to use autonomous vehicles	Comparative and psychological perspectives that can affect user-vehicle relationships (SAE levels)	Structural equation modeling; partial least squares	Technology acceptance model; factors for autonomous vehicle use: psychological ownership	PU, PEOU, BI; extensions: perceived risk, self-efficacy, relative advantage, psychological ownership	Literature-based	• Self-efficacy, PU, psychological ownership → BI (+)<br>• Perceived risk → BI (-)<br>• Self-efficacy → PEOU<br>• Relative advantage, PEOU → PU<br>• *Psychological ownership → PU, PEOU, perceived risk*<br>• *PEOU, relative advantage → BI*<br>• *Self-efficacy → perceived risk*
Leicht et al. (2018)	Analyzing the moderating effects of consumer innovativeness on the relationships between different antecedents of autonomous vehicle purchase intention	Mass market adoption (purchase intention) of fully autonomous cars (SAE level 5)	Structural equation modeling; multi-group analysis	Unified theory of acceptance and use of technology	Performance expectancy, effort expectancy, and social influence, BI; extension: consumer innovativeness	Literature-based	• Performance expectancy, effort expectancy, social influence → purchase intention (+)<br>• Consumer innovativeness as a moderator (higher effects when consumer innovativeness is high)
Liu et al. (2018)	Investigating three acceptance measures of autonomous vehicles: general acceptance, willingness to pay, and behavioral intention	Public acceptance of autonomous vehicles with regard to social trust and risk/benefit perceptions (SAE level 5)	Structural equation modeling; prediction analysis; partial least squares	Trust heuristic; psychological model	Social trust, perceived risk, PB, BI, general acceptance, willingness to pay	Theory-based	• Social trust → general acceptance, willingness to pay, BI<br>• Social trust also has a significant positive indirect effect on all three through PB<br>• Perceived risk → willingness to pay, general acceptance (-)
Madigan et al. (2017)	Investigating the factors that influence the public's acceptance of automated road transport systems	Behavioral intention toward automated public transport in Germany (CityMobil2 automated road transport systems) (SAE level 4)	Multiple regression analysis	Unified theory of acceptance and use of technology II	Performance expectancy, effort expectancy, social influence, PBC, facilitating conditions, hedonic motivation, age, gender, experience	–	• Performance expectancy, social influence, facilitating conditions, hedonic motivation → BI (+)<br>• *Effort expectancy, age, gender, and experience → BI*<br>• *No moderating effects*
Manfreda et al. (2019)	Investigating millennials' adoption of autonomous vehicles by considering the importance of technology adoption, perception of benefits, security, safety, mobility-related efficiencies, and concerns	Adoption of fully automated vehicles within smart cities from the millennial point of view (no level assignment to SAE or NHTSA)	Structural equation modeling	Unified theory of acceptance and use of technology	Technological mindset, personal and societal benefits, security, mobility-related efficiencies, safety, technological and legal concerns, adoption of autonomous vehicles	Literature-based	• Technological mindset, safety, personal and societal benefits → adoption of autonomous vehicles (+)<br>• Technological and legal concerns significantly influence adoption of autonomous vehicles (-)<br>• Safety → technological and societal concerns (-)<br>• *Security and mobility-related efficiencies → adoption of autonomous vehicles*
Motamedi et al. (2020)	Developing user acceptance models for personally owned and shared autonomous vehicles.	Acceptance of personally owned vs. shared automated vehicles in California (SAE level 5)	Focus groups, confirmatory factor analysis; structural equation modeling	Technology acceptance model, car technology acceptance model	PEOU, PU, perceived safety, BI; extension: trust, compatibility	Literature-based	• Almost same results for personally owned and shared concepts<br>• Compatibility →trust (+)<br>• Trust → perceived safety (+)<br>• Perceived safety → PU, PEOU (+)<br>• PEOU → PU (+)<br>• PU → BI (+)<br>• Compatibility, trust → PU (+)<br>• Perceived safety, trust → PEOU (+)<br>• PEOU → BI (+)
Nishihori et al. (2020)	Examining the current situation and determining factors regarding the social acceptance of autonomous vehicles	Acceptance of autonomous vehicles in Japan	Meta-analysis	–	Pros and cons, BI; explanatory variables: gender, driver's license, car ownership, occupation, family composition, population density, risk perception of dreadful for autonomous vehicles, risk perception of unknown for autonomous vehicles, recognition level of demonstration experiment, BI	–	• Correlation pros and cons and BI (+)<br>• Pros and cons differ by gender, driver license, car ownership, family composition, population density, risk perception for autonomous vehicles, and recognition level of autonomous vehicles demonstration experiment<br>• BI, recognition level of autonomous vehicles demonstration experiment, gender, population density, risk perception of autonomous vehicles → pros and cons

Nordhoff et al. (2018)	Investigating the acceptance of driverless vehicles	Autonomous shuttles; cross-national questionnaire (no level assignment to SAE or NHTSA)	Descriptive analysis, principal component analysis	–	–	–	• Participants would enjoy taking a ride in a driverless vehicle<br>• Identification of new components (e.g., being comfortable with technology, supporting a car-free environment)<br>• Acceptance is more strongly determined by domain-specific ATT than by sociodemographic characteristics
Paddeu et al. (2020)	Investigating which attributes of traveling in a shared autonomous vehicle affect comfort and trust ratings	Acceptance (indicated by trust and comfort) of a real-world shared autonomous vehicle	Two-way ANOVA	-	Direction of face, speed, trust, comfort, nausea	–	• Direction of face → trust (forward +)<br>• Speed → trust (-)<br>• Direction of face → comfort<br>• Speed → comfort (-)
Panagiotopoulos and Dimitrakopoulos (2018)	Investigating factors that influence consumers' intentions to use and accept autonomous vehicles	Behavioral intention toward partially or fully autonomous and self-driving vehicles (SAE level 5)	Descriptive analysis; multiple regression analysis; structural equation modeling	Technology acceptance model	PU, PEOU, BI; extensions: perceived trust, social influence	Literature-based	• PU, PEOU, perceived trust, social influence → BI (+)<br>• PEOU → PU (+)
Payre et al. (2014)	Investigating drivers' intention to use fully automated vehicles	Predicting intention by a priori acceptability, attitudes, personality traits, and behavioral adaptation toward automated driving (NHTSA level 4)	Confirmatory factor analysis; descriptive analysis; hierarchical linear regression analysis	Priori acceptability (based on the technology acceptance model)	PU, BI; extensions: ATT, sensation-seeking, locus of control, interest in impaired driving, contextual acceptability	Literature-based	• ATT → BI (+)<br>• Driving-related sensation-seeking, being impaired → BI (+)<br>• Men have a stronger ATT and higher BI than women
Rahman et al. (2019)	Investigating older adults' (age > 60) perception of self-driving vehicles from the perspective of users and pedestrians as well as their willingness to use them	Self-driving vehicles in the context of people who cannot drive (medical- or age-related reasons) (SAE level 5)	Confirmatory factor analysis	Technology acceptance model; theory of planned behavior; automation acceptance model	ATT, PU, BI, social norm; trust, compatibility; extensions: demographic variables	Theory- and literature-based	• ATT, PU, trust, compatibility → acceptance (+)<br>• ATT, trust, social norms were rated positively by users and neutral by pedestrians<br>• PU was rated positively by both users and pedestrians<br>• Compatibility was rated negatively by both users and pedestrians
Ro and Ha (2019)	Exploring consumers' expectations for autonomous cars in Korea	Autonomous cars in Korea (no level assignment to SAE or NHTSA)	Factor analysis	Theory of reasoned action; unified theory of acceptance and use of technology	Expectation in terms of convenience, safety, monetary costs, economy, accountability, ethics, and licensing (derived by exploratory factor analysis)	Theory- and literature-based	• Expectations regarding convenience, safety → ATT, BI (+)<br>• Expectations regarding monetary costs → ATT, BI (-)<br>• Expectations regarding ethics and licensing → ATT (+)<br>• *Expectations regarding ethics and licensing significantly → BI*<br>• *Expectations regarding economy and accountability → ATT, BI*<br>• ATT → BI (+)
Roedel et al. (2014)	Investigating how user acceptance and user experience factors differ with levels of autonomy	User experience and acceptance toward automated vehicles (NHTSA levels 0–4)	Descriptive analysis, mean comparison	Technology acceptance model; theory of planned behavior	PEOU, ATT toward the system, BI, PBC; extensions: trust, fun	Theory- and literature-based	• User acceptance and experience differ significantly between levels of autonomy<br>• PEOU → ATT, BI (+)<br>• PBC, trust, and fun decrease with higher levels of autonomy
Schoettle and Sivak (2014)	Investigating public opinion about autonomous and self-driving vehicles	Focus on the U.S., the U.K., and Australia (NHTSA level 4)	Descriptive analysis	–	–	–	• Positive initial opinion of the technology<br>• High expectations about the benefits of the technology<br>• High levels of concern about riding a self-driving car and security issues<br>• High desire to have this technology in the vehicle
Talebian and Mishra (2018)	Predicting the adoption of connected autonomous vehicles	Influence of peer-to-peer communication on willingness to pay and adoption of connected autonomous vehicles (no level assignment to SAE or NHTSA)	Descriptive analysis; sensitivity analysis; discrete choice modeling; agent-based simulation modeling	Theory of diffusion of innovations; agent-based modeling	Functional barriers, psychological barriers, incentives, peer-to-peer communication, media, willingness to pay	Theory- and literature-based	• Perceptions are dynamic and change through peer-to-peer communication and media exposure, while to-pay is dynamic and changes as result of per-to-peer communication<br>• Resistance defers adoption

Wu et al. (2019)	Investigating the role of environmental concern in the public acceptance of autonomous electric vehicles	Public acceptance of autonomous electric vehicles in China (no level assignment to SAE or NHTSA)	Structural equation modeling; confirmatory factor analysis	Technology acceptance model	Green PU, PEOU, BI;<br>extension: environmental concern	Literature-based	• Green PU, PEOU, and environmental concerns→ BI (+)
Xu et al. (2018)	Understanding the influence of direct experience of an automated vehicle (SAE level 3); explaining and predicting public acceptance of autonomous vehicles through a psychological model	Experiencing SAE level 3 vehicles and their implication on the acceptance on SAE level 5 vehicles in China	Mediation analysis; out-of-sample prediction analysis; structural equation modeling	Technology acceptance model	PU, PEOU, BI;<br>extensions: willingness to re-ride, trust, perceived safety	Literature-based	• Experience with a SAE level 3 vehicle increases trust, PU, and PEOU but not BI<br>• PU, trust, perceived safety → BI, willingness to re-ride (+)<br>• PEOU → BI (+)<br>• Trust → PU, PEOU, perceived safety (+)
Yuen et al. (2020)	Investigating determinants of public acceptance of autonomous cars from an innovation diffusion perspective	Acceptance of autonomous vehicles in Korea (no level assignment to SAE or NHTSA)	Structural equation modeling	Innovation diffusion theory; perceived value theory;<br>trust theory	Relative advantage, compatibility, complexity, trialability, observability; perceived value; trust; public acceptance	Theory-based	• Relative advantage, compatibility, trialability, observability → perceived value (+)<br>• Complexity → perceived value (-)<br>• Perceived value → trust, public acceptance (+)<br>• Trust → public acceptance (+)
Zhang et al. (2019)	Exploring factors affecting users' acceptance of automated vehicles	China<br>(SAE level 3)	Structural equation modeling	Technology acceptance model	PEOU, PU, ATT toward using, BI;<br>extensions: perceived safety risk, perceived privacy risk, initial trust	Literature-based	• ATT, PU → BI (+)<br>• Initial trust, PEOU → ATT (+)<br>• *PU → ATT*<br>• Perceived safety risk → initial trust (-)<br>• PU → initial trust (+)<br>• *PEOU, perceived privacy risk → initial trust*
Zhang et al. (2020)	Investigating the role of social and personal factors in automated vehicle acceptance	Intention to use automated vehicles in China (SAE level 3)	Structural equation modeling	Technology acceptance model	PEOU, PU, BI;<br>extensions: initial trust, social influence, big five, sensation-seeking	Literature-based	• PEOU, PU, trust, social influence, sensation-seeking → BI (+)<br>• Social influence → PEOU, PU, trust (+)<br>• From the Big Five, only neuroticism significantly negatively influences trust and openness significantly positively influences it<br>• *> The other personality traits have no significant effect.*

SAE = Society of Automotive Engineers International; NHTSA = National Highway Traffic Safety Administration; ATT = attitude; BI = behavioral intention; CC = collaborative consumption; PEOU = perceived ease of use; PU = perceived usefulness; PB = perceived benefit; PBC = perceived behavioral control; SN = subjective norm; (+) = positive relationship; (-) = negative relationship; *italicized = not significant.*

Appendix B Results of interviews and construct definitions of related acceptance criteria.

Example statements	Related acceptance criteria	Original construct definition	References
I like driving myself and being in control of the vehicle.	Desire to exert control	The ability to control events in one's own life.	Burger and Cooper (1979)
I can imagine that autonomous driving can be really relaxing and, therefore, more fun because you can concentrate completely on other things, such as computer games or other fun activities.	Perceived enjoyment	The degree to which using a technology is perceived to be enjoyable as an activity, apart from any performance consequences that may be anticipated.	Davis et al. (1992)
I would have to be sure that the technology is safe [...], 100% trust is a requirement.	Trust	The system of beliefs concerning predictability and functionality of system characteristics.	Thatcher et al. (2011)
There should be an appropriate cost–benefit ratio [...] whether it is worth paying for it.	Price evaluation	Individual's evaluation of the trade-off between the perceived benefits of a technology and the monetary cost for using it.	Dodds et al. (1991)
As I am concerned with environmental protection and other environmental aspects, autonomous driving is generally interesting for me if it contributes to sustainability.	Ecological awareness	Individual's behavior which accounts for the influence on ecological impacts.	Roberts (1995)
For me it is important that there exists transparency on the protection of personal data before I would consider using autonomous vehicles.	Privacy concerns	Individual's concern about gathering and use of individual-specific consumer information.	Phelps et al. (2000)
In general, I would say that it is important if other people have had good experience with autonomous driving and what my social environment thinks about it.	Subjective norm	Perceived social pressures to perform or not perform a specific behavior.	Ajzen (1991)
For me personally, as someone who is interested in new technologies, to try out this high-tech innovation would be very interesting.	Personal innovativeness	Individual's willingness to try out a new technology.	Agarwal and Prasad (1998)
The first thing that I associate with autonomous driving is that I can do whatever I want when driving. This is a great advantage compared	Relative advantage	The extent to which an innovation is perceived as better than the idea it supersedes.	Rogers (2003)

to conventional vehicles. I can, for example, prepare a last-minute appointment while driving.			
As part of my job, I travel a lot to different places. The infrastructure for autonomous vehicles needs to be developed in a way that I could use autonomous vehicles everywhere, for example, for long distances or in rural areas.	Compatibility	The extent to which an innovation is perceived as consistent with the existing values, prior experiences, and needs of potential adopters.	Rogers (2003)

Appendix C Construct conceptualization regarding autonomous driving.

Construct (number of items)	Sample items	Conceptualization	Related references
Desire to exert control (3)	I would prefer to be in control when using autonomous vehicles.<br>I would prefer to make my own decisions when driving instead of being guided by the autonomous vehicle technology.<br>It is not important for me to be able to take back control from an autonomous vehicle.*	The degree to which an individual has a demand for control in connection with autonomous driving.	Burger and Cooper (1979); Nees (2016); Planing (2014)
Privacy concerns (4)	I believe that my privacy would be compromised when using autonomous vehicles.<br>I believe that my data would be secure when using autonomous vehicles.*<br>I would be worried about my privacy when using autonomous vehicles.<br>I believe that using autonomous vehicles would threaten my privacy.	The individual's concern about the collection and use of individual-specific consumer information when using autonomous driving.	Nasri and Charfeddine (2012); Vijayasarathy (2004)
Trust (6)	Overall, I would trust autonomous vehicles.<br>I expect autonomous vehicles to be reliable.<br>I expect autonomous vehicles to be dependable.<br>Overall, I would trust autonomous vehicle technology.<br>I believe that using autonomous vehicles will be safe.<br>Overall, I would not trust an autonomous vehicle to get me safely to my destination.*	The ability to forecast the predictability and functionality of autonomous driving.	Choi and Ji (2015); Nasri and Charfeddine (2012); Nees (2016)
Ecological awareness (4)	The impact of autonomous vehicles on the environment would influence my usage decision.<br>The degree to which autonomous vehicles cause pollution would influence my usage decision.<br>If I understand the impact autonomous vehicles have on the environment, it will influence my usage decision.<br>The extent to which autonomous vehicles impact the environment would not influence my usage decision.*	The individual's perception of behaving ecologically correctly when using autonomous driving.	Roberts (1995)
Personal innovativeness (5)	I would like to experiment with autonomous vehicles.<br>Among my peers, I would be one of the first to try out autonomous vehicles.<br>In general, I am hesitant to try out autonomous vehicles.*<br>If I heard about a new development in the area of autonomous vehicles, I would look for ways to experiment with it.<br>Generally, I would not like to experiment with autonomous vehicles.*	The willingness of an individual to try out autonomous driving.	Agarwal and Prasad (1998); Parasuraman (2000)
Relative advantage (5)	Using autonomous vehicles would enable me to accomplish driving tasks more efficiently.<br>Using autonomous vehicles would improve my driving performance.<br>Overall, I think using autonomous driving will be disadvantageous for me.*<br>Using autonomous vehicles would not improve my driving performance.*<br>Using autonomous vehicles would enable me to accomplish driving tasks more effectively.	The degree to which autonomous driving is perceived as better than current available mobility options.	Moore and Benbasat (1991)
Compatibility (3)	Using autonomous vehicles would be compatible with my mobility behavior.<br>I think using autonomous vehicles would not fit well into mobility behavior.*<br>I think using autonomous vehicles is compatible with all aspects of my mobility behavior.	The degree to which autonomous driving is perceived as consistent with existing mobility options.	Moore and Benbasat (1991)
Enjoyment (3)	I expect using autonomous vehicles to be enjoyable.<br>I expect using autonomous vehicles to be pleasant.<br>I expect using autonomous vehicles to be unpleasant.*	The degree to which using autonomous driving is perceived to be enjoyable in its own right, apart from any performance consequences that may be anticipated.	Venkatesh and Bala (2008)
Price evaluation (3)	The benefits of autonomous vehicles would outweigh the amount of money they would cost.<br>I would be willing to pay more to use autonomous vehicles.	Individual's evaluation of the trade-off between the perceived benefits of using autonomous vehicles and the monetary cost.	Nees (2016); Venkatesh et al. (2012)

	I expect the costs of using autonomous vehicles to outweigh the value of using autonomous vehicles.*		
Subjective norm (4)	People who influence my behavior would think that I should use autonomous vehicles.<br>People who are important to me think that I should use autonomous vehicles.<br>People who influence me would think that I should use autonomous vehicles.<br>People whose opinions I value would prefer that I do not use autonomous vehicles.*	The perceived social pressure to use autonomous vehicles.	Nasri and Charfeddine (2012); Venkatesh and Bala (2008)
Perceived usefulness (4)	Autonomous vehicles would be useful to me.<br>Using autonomous vehicles would increase my productivity.<br>Autonomous driving would not be useful to me.*<br>Using autonomous vehicles would allow me to be more productive.	The degree to which autonomous driving results in a positive use–performance relationship.	Nees (2016); Choi and Ji (2015); Venkatesh and Davis (2000)
Perceived ease of use (3)	I expect learning to use autonomous vehicles will be easy for me.<br>I expect autonomous vehicles will be easy to use.<br>I think that interacting with autonomous vehicles would require a lot of mental effort.*	The degree to which an individual believes that using autonomous driving is free of effort.	Nees (2016); Choi and Ji (2015); Venkatesh and Davis (2000)
Attitude (6)	I think that using autonomous vehicles is a wise idea.<br>I think that using autonomous vehicles is a good idea.<br>In my opinion, it is desirable to use autonomous vehicles.<br>I like the idea of using autonomous vehicles.<br>I think that using autonomous vehicles would be beneficial for me.<br>I think that using autonomous vehicles is a bad idea.*	The degree to which an individual has a favorable or unfavorable evaluation of using autonomous driving.	Nasri and Charfeddine (2012); Taylor and Todd (1995)
Usage intention (4)	If I had access to an autonomous vehicle, I predict that I would use it.<br>Assuming I had access to an autonomous vehicle, I intend to use it.<br>I intend to use autonomous vehicles in the future.<br>Assuming I had access to an autonomous vehicle, I predict that I would not use it.*	The individual's motivational readiness to use or not to use autonomous driving.	Choi and Ji (2015); Nees (2016); Venkatesh (2000)

*Reverse-coded item.

Appendix D Factor loadings (in bold) and cross loadings.

	ATT	CO	EA	DEC	EN	PC	PE	PEOU	PI	PU	RA	SN	TR	UI
**ATT01**	**0.93**	0.63	0.12	−0.40	0.78	0.32	0.63	0.49	0.33	0.73	0.73	0.55	0.67	0.77
**ATT02**	**0.90**	0.63	0.09	−0.50	0.70	0.30	0.61	0.53	0.39	0.73	0.74	0.56	0.74	0.79
**ATT03**	**0.94**	0.68	0.13	−0.46	0.78	0.32	0.64	0.54	0.39	0.75	0.74	0.59	0.73	0.78
**ATT04**	**0.71**	0.53	0.14	−0.25	0.70	0.24	0.46	0.31	0.26	0.54	0.53	0.41	0.43	0.51
**ATT05**	**0.92**	0.63	0.10	−0.40	0.72	0.33	0.60	0.54	0.33	0.76	0.76	0.55	0.68	0.72
**ATT06**	**0.90**	0.61	0.13	−0.40	0.69	0.28	0.61	0.49	0.34	0.72	0.76	0.53	0.65	0.72
**CO01**	0.65	**0.93**	0.17	−0.42	0.60	0.34	0.64	0.43	0.43	0.67	0.63	0.53	0.61	0.65
**CO02**	0.60	**0.92**	0.08	−0.39	0.58	0.33	0.51	0.43	0.34	0.58	0.56	0.51	0.56	0.59
**CO03**	0.65	**0.88**	0.10	−0.40	0.60	0.35	0.58	0.45	0.41	0.61	0.61	0.49	0.57	0.57
**EA01**	0.05	0.09	**0.77**	−0.05	0.12	0.02	0.04	0.02	0.17	0.09	0.12	0.11	0.12	0.07
**EA02**	0.12	0.16	**0.87**	−0.05	0.14	0.02	0.15	0.05	0.09	0.15	0.15	0.08	0.14	0.11
**EA03**	0.07	0.07	**0.82**	0.06	0.15	0.04	0.09	0.03	0.07	0.08	0.11	0.08	0.07	0.04
**EA04**	0.16	0.08	**0.84**	−0.02	0.21	0.02	0.05	0.05	0.03	0.12	0.17	0.16	0.13	0.13
**DEC01**	−0.37	−0.34	0.02	**0.87**	−0.33	−0.33	−0.30	−0.35	−0.20	−0.36	−0.33	−0.27	−0.46	−0.36
**DEC02**	−0.44	−0.42	0.02	**0.92**	−0.35	−0.32	−0.38	−0.38	−0.20	−0.39	−0.36	−0.31	−0.51	−0.43
**DEC03**	−0.35	−0.38	−0.10	**0.76**	−0.29	−0.24	−0.31	−0.26	−0.20	−0.32	−0.34	−0.35	−0.42	−0.35
**EN01**	0.85	0.69	0.16	−0.46	**0.92**	0.39	0.68	0.51	0.40	0.76	0.80	0.58	0.76	0.79
**EN02**	0.58	0.43	0.13	−0.20	**0.83**	0.28	0.44	0.29	0.28	0.47	0.51	0.40	0.39	0.45
**EN03**	0.67	0.54	0.21	−0.28	**0.90**	0.34	0.55	0.32	0.27	0.57	0.62	0.46	0.54	0.56
**PC01**	0.33	0.38	0.04	−0.34	0.39	**0.95**	0.34	0.31	0.26	0.31	0.35	0.31	0.39	0.30
**PC02**	0.34	0.38	0.05	−0.33	0.41	**0.96**	0.35	0.34	0.25	0.31	0.36	0.33	0.39	0.32
**PC03**	0.20	0.21	0.01	−0.25	0.23	**0.73**	0.23	0.26	0.17	0.18	0.23	0.15	0.29	0.21
**PC04**	0.30	0.33	−0.01	−0.32	0.34	**0.90**	0.30	0.31	0.29	0.31	0.34	0.30	0.36	0.28
**PE01**	0.58	0.49	0.11	−0.33	0.57	0.32	**0.81**	0.32	0.29	0.57	0.54	0.44	0.51	0.51
**PE02**	0.63	0.62	0.10	−0.37	0.61	0.31	**0.96**	0.38	0.38	0.64	0.62	0.48	0.59	0.62
**PE03**	0.61	0.61	0.09	−0.36	0.59	0.31	**0.95**	0.38	0.35	0.63	0.62	0.46	0.59	0.61
**PEOU01**	0.48	0.41	−0.01	−0.35	0.38	0.29	0.38	**0.91**	0.26	0.47	0.48	0.40	0.56	0.42
**PEOU02**	0.58	0.49	0.11	−0.39	0.48	0.32	0.41	**0.92**	0.32	0.53	0.56	0.42	0.57	0.48
**PEOU03**	0.33	0.32	−0.01	−0.27	0.27	0.30	0.20	**0.75**	0.21	0.32	0.40	0.30	0.39	0.26
**PI01**	0.14	0.23	0.08	−0.15	0.07	0.10	0.18	0.18	**0.70**	0.18	0.14	0.11	0.23	0.18
**PI02**	0.34	0.37	0.07	−0.19	0.32	0.19	0.31	0.29	**0.84**	0.39	0.40	0.26	0.39	0.32
**PI03**	0.37	0.39	0.09	−0.23	0.38	0.32	0.38	0.30	**0.88**	0.42	0.40	0.33	0.42	0.34
**PI04**	0.27	0.36	0.05	−0.18	0.27	0.21	0.31	0.22	**0.82**	0.33	0.27	0.32	0.31	0.28
**PI05**	0.35	0.35	0.15	−0.16	0.34	0.23	0.28	0.24	**0.76**	0.32	0.33	0.26	0.36	0.34
**PU01**	0.61	0.56	0.03	−0.27	0.54	0.26	0.54	0.36	0.41	**0.78**	0.63	0.54	0.51	0.55
**PU02**	0.60	0.56	0.18	−0.39	0.62	0.30	0.55	0.39	0.31	**0.82**	0.66	0.50	0.66	0.59

**PU03**	0.60	0.55	0.19	−0.34	0.50	0.21	0.51	0.47	0.32	**0.83**	0.70	0.49	0.61	0.53
**PU04**	0.82	0.60	0.07	−0.39	0.68	0.28	0.64	0.50	0.37	**0.88**	0.80	0.56	0.70	0.68
**RA01**	0.74	0.59	0.16	−0.33	0.68	0.34	0.61	0.53	0.39	0.78	**0.89**	0.59	0.67	0.62
**RA02**	0.43	0.36	0.19	−0.17	0.50	0.23	0.37	0.24	0.13	0.54	**0.69**	0.45	0.38	0.38
**RA03**	0.72	0.60	0.10	−0.31	0.62	0.31	0.55	0.53	0.42	0.68	**0.85**	0.50	0.59	0.54
**RA04**	0.73	0.61	0.10	−0.36	0.69	0.30	0.61	0.44	0.36	0.73	**0.87**	0.57	0.64	0.66
**RA05**	0.59	0.49	0.17	−0.44	0.57	0.29	0.48	0.50	0.28	0.70	**0.76**	0.44	0.73	0.52
**SN01**	0.54	0.50	0.12	−0.32	0.46	0.26	0.47	0.39	0.31	0.56	0.55	**0.88**	0.46	0.48
**SN02**	0.54	0.53	0.12	−0.34	0.50	0.28	0.46	0.42	0.32	0.59	0.61	**0.89**	0.51	0.50
**SN03**	0.57	0.52	0.09	−0.33	0.55	0.30	0.47	0.39	0.28	0.58	0.58	**0.92**	0.51	0.53
**SN04**	0.52	0.48	0.13	−0.30	0.51	0.30	0.41	0.37	0.29	0.53	0.52	**0.90**	0.48	0.52
**TR01**	0.73	0.61	0.14	−0.52	0.67	0.39	0.60	0.56	0.43	0.73	0.71	0.53	**0.95**	0.72
**TR02**	0.69	0.60	0.11	−0.55	0.64	0.40	0.59	0.55	0.42	0.71	0.71	0.52	**0.96**	0.66
**TR03**	0.71	0.62	0.15	−0.54	0.67	0.39	0.60	0.54	0.42	0.73	0.72	0.52	**0.97**	0.68
**TR04**	0.67	0.62	0.15	−0.50	0.60	0.35	0.58	0.59	0.40	0.72	0.71	0.50	**0.95**	0.63
**TR05**	0.72	0.62	0.15	−0.53	0.65	0.39	0.60	0.59	0.41	0.74	0.73	0.53	**0.97**	0.71
**TR06**	0.73	0.61	0.14	−0.51	0.67	0.40	0.62	0.59	0.43	0.73	0.73	0.54	**0.96**	0.69
**UI01**	0.80	0.64	0.10	−0.39	0.67	0.27	0.61	0.45	0.35	0.69	0.65	0.49	0.67	**0.93**
**UI02**	0.78	0.65	0.10	−0.44	0.68	0.33	0.60	0.46	0.38	0.69	0.67	0.55	0.69	**0.95**
**UI03**	0.73	0.59	0.07	−0.39	0.65	0.30	0.59	0.44	0.36	0.64	0.61	0.53	0.65	**0.89**
**UI04**	0.42	0.36	0.14	−0.32	0.43	0.17	0.37	0.17	0.13	0.35	0.34	0.35	0.38	**0.61**

ATT: attitude; CO: compatibility; EA: ecological awareness; DEC: desire to exert control; EN: enjoyment; PC: privacy concerns; PE: price evaluation; PEU: perceived ease of use; PI: personal innovativeness; PU: perceived usefulness; RA: relative advantage; SN: subjective norm; TR: trust; UI: usage intention.

References

Acheampong, R.A., Cugurullo, F., 2019. Capturing the behavioural determinants behind the adoption of autonomous vehicles: conceptual frameworks and measurement models to predict public transport, sharing and ownership trends of self-driving cars. Transp. Res. Part F Traffic Psychol. Behav. 62, 349–375. https://doi.org/10.1016/j.trf.2019.01.009.

Adnan, N., Md Nordin, S., bin Bahruddin, M.A., Ali, M., 2018. How trust can drive forward the user acceptance to the technology? In-vehicle technology for autonomous vehicle. Transp. Res. Part A Policy Pract. 118, 819–836. https://doi.org/10.1016/j.tra.2018.10.019.

Anania, E.C., Rice, S., Walters, N.W., Pierce, M., Winter, S.R., Milner, M.N., 2018. The effects of positive and negative information on consumers' willingness to ride in a driverless vehicle. Transp. Policy Oxf. 72, 218–224. https://doi.org/10.1016/j.tranpol.2018.04.002.

Agarwal, R., Prasad, J., 1998. A conceptual and operational definition of personal innovativeness in the domain of information technology. Inf. Syst. Res. 9 (2), 204–215. https://doi.org/10.1287/isre.9.2.204.

Ajzen, I., 1991. The theory of planned behaviour. Organ. Behav. Hum. Decis. Process. 50 (2), 179–211. https://doi.org/10.1016/0749-5978(91)90020-T.

Ajzen, I., Fishbein, M., 1980. Understanding Attitudes and Predicting Social Behavior. Prentice-Hall, Englewood Cliffs.

Anderson, J.C., Gerbing, D.W., 1988. Structural equation modeling in practice. A review and recommended two-step approach. Psychol. Bull. 103 (3), 411–423. https://doi.org/10.1037/0033-2909.103.3.411.

Anderson, J.M., Kalra, N., Stanley, K.D., Sorensen, P., Samaras, C., Oluwatola, O.A., 2016. Autonomous Vehicle Technology: A Guide for Policymakers. RAND Corporation, Santa Monica, California.

Bagloee, S.A., Tavana, M., Asadi, M., Oliver, T., 2016. Autonomous vehicles: challenges, opportunities, and future implications for transportation policies. J. Mod. Transp. 24 (4), 284–303. https://doi.org/10.1007/s40534-016-0117-3.

Bagozzi, R., 2007. The legacy of the technology acceptance model and a proposal for a paradigm shift. J. Assoc. Inf. Syst. 8, 244–254. https://doi.org/10.17705/1jais.00122.

Bagozzi, R.P., Yi, Y., 1988. On the evaluation of structural equation models. J. Acad. Mark. Sci. 16 (1), 74–94. https://doi.org/10.1007/BF02723327.

Bansal, P., Kockelman, K.M., 2017. Forecasting Americans' long-term adoption of connected and autonomous vehicle technologies. Transp. Res. Part A Policy Pract. 95, 49–63. https://doi.org/10.1016/j.tra.2016.10.013.

Bansal, P., Kockelman, K.M., Singh, A., 2016. Assessing public opinions of and interest in new vehicle technologies: an Austin perspective. Transp. Res. Part C Emerg. Technol. 67, 1–14. https://doi.org/10.1016/j.trc.2016.01.019.

Becker, F., Axhausen, K.W., 2017. Literature review on surveys investigating the acceptance of automated vehicles. Transp. Amst. 44, 1293–1306. https://doi.org/10.1007/s11116-017-9808-9.

Begg, D., 2014. Vision for London : A 2050 Vision for London. Available at: https://www.transporttimes.co.uk/Admin/uploads/64165-transport-times_a-2050-vision-for-london_aw-web-ready.pdf.

Beiker, S.A., 2012. Legal aspects of autonomous driving. Santa Clara Law Rev. 52 (4), 1144–1156. https://digitalcommons.law.scu.edu/lawreview/vol52/iss4/1.

Benbasat, I., Barki, H., 2007. Quo vadis TAM? J. Assoc. Inf. Syst. 8, 211–218. https://doi.org/10.17705/1jais.00126.

Benleulmi, A.Z., Blecker, T., 2017. Investigating the factors influencing the acceptance of fully autonomous cars. In: Proceedings of the Hamburg International Conference of Logistics, pp. 99–115. https://doi.org/10.15480/882.1449.

Bernhard, C., Oberfeld, D., Hoffmann, C., Weismüller, D., Hecht, H., 2020. User acceptance of automated public transport: valence of an autonomous minibus experience. Transp. Res. Part F Traffic Psychol. Behav. 70, 109–123. https://doi.org/10.1016/j.trf.2020.02.008.

Bertoncello, M., Wee, D., 2015. Ten Ways Autonomous Driving Could Redefine the Automotive World. McKinsey & Company Technical reportAvailable at: http://www.mckinsey.com/practice-clients/automotive-assembly/ten-ways-autonomous-driving-could-redefine-the-automotive-world.

Bhattacherjee, A., Premkumar, G., 2004. Understanding changes in belief and attitude toward information technology usage: a theoretical model and longitudinal test. MIS Q. 28 (2), 229–254. https://doi.org/10.2307/25148634.

Bjørner, T., 2015. A priori user acceptance and the perceived driving pleasure in semi-autonomous and autonomous vehicles. In: Proceedings of the European Transport Conference. Frankfurt.

Boesch, P.M., Becker, F., Becker, H., Axhausen, K.W., 2018. Cost-based analysis of autonomous mobility services. Transp. Policy Oxf. 64, 76–91. https://doi.org/10.1016/j.tranpol.2017.09.005.

Bonnefon, J.F., Shariff, A., Rahwan, I., 2016. The social dilemma of autonomous vehicles. Science 352 (6293), 1573–1576. https://doi.org/10.1126/science.aaf2654.

Brandts, J., Charness, G., 2000. Hot vs. cold: sequential responses and preference stability in experimental games. Exp. Econ. 2 (3), 227–238. https://doi.org/10.1023/A:1009962612354.

Brell, T., Philipsen, R., Ziefle, M., 2019. sCARy! Risk perceptions in autonomous driving: the influence of experience on perceived benefits and barriers. Risk Anal. 39, 342–357. https://doi.org/10.1111/risa.13190.

Brendel, A.B., Brennecke, J.T., Nastjuk, I., 2018. Applying econophysics in the context of carsharing: development of a vehicle relocation algorithm and decision support system. In: Proceedings of the International Conference on Information Systems (ICIS). San Francisco. pp. 1–17.

Brown, A., Gonder, J., Repac, B., 2014. An analysis of possible energy impacts of automated vehicle. In: Meyer, G., Beiker, S. (Eds.), Road Vehicle Automation. Springer International Publishing, Switzerland, pp. 137–153.

Bruner, G.C., Kumar, A., 2005. Explaining consumer acceptance of handheld internet devices. J. Bus. Res. 58 (5), 556–558. https://doi.org/10.1016/j.jbusres.2003.08.002.

Buckley, L., Kaye, S.-A., Pradhan, A.K., 2018a. Psychosocial factors associated with intended use of automated vehicles: a simulated driving study. Accid. Anal. Prev. 115, 202–208. https://doi.org/10.1016/j.aap.2018.03.021.

Buckley, L., Kaye, S.-A., Pradhan, A.K., 2018b. A qualitative examination of drivers' responses to partially automated vehicles. Transp. Res. Part F Traffic Psychol. Behav. 56, 167–175. https://doi.org/10.1016/j.trf.2018.04.012.

Burger, J.M., Cooper, H.M., 1979. The desirability of control. Motiv. Emot. 3 (4), 381–393. https://doi.org/10.1007/BF00994052.

Carter, L., Bélanger, F., 2005. The utilization of egovernment services: citizen trust, innovation and acceptance factors. Inf. Syst. J. 15 (1), 5–25. https://doi.org/10.1111/j.1365-2575.2005.00183.x.

Casley, S.V., Jardim, A.S., Quartulli, A.M., 2013. A Study of Public Acceptance of Autonomous Cars, Interactive Qualifying Project. Worcester Polytechnic Institute Available at: https://web.wpi.edu/Pubs/E-project/Available/E-project-043013-155601/unrestricted/A_Study_of_Public_Acceptance_of_Autonomous_Cars.pdf.

Chenail, R.J., 2011. Interviewing the investigator: strategies for addressing instrumentation and researcher bias concerns in qualitative research. Qual. Rep. 16 (1), 255–262.

Chin, W.W., 1998. The partial least squares approach to structural equation modelling. Mod. Methods Bus. Res. 295 (2), 295–336.

Chin, W.W., Johnson, N., Schwarz, A., 2008. A fast form approach to measuring technology acceptance and other constructs. Manag. Inf. Syst. Q. 32 (4), 687–703. https://doi.org/10.2307/25148867.

Cho, Y., Park, J., Park, S., Jung, E.S., 2017. Technology Acceptance Modeling Based on User Experience for Autonomous Vehicles. J. Ergon. Soc. Korea 36 (2), 87–108.

Choi, J.K., Ji, Y.G., 2015. Investigating the importance of trust on adopting an autonomous vehicle. J. Hum. Comput. Interact. 31 (10), 692–702. https://doi.org/10.1080/10447318.2015.1070549.

Clifton, K.J., Handy, S.L., 2003. Qualitative methods in travel behaviour research. In:

Jones, P., Stopher, P.R. (Eds.), Transport Survey Quality and Innovation. Emerald Group Publishing Limited, New York, pp. 283–302.
Creswell, J.W., 2007. Qualitative Inquiry and Research Design: Choosing Among Five Approaches. Sage, Thousand Oaks.
Creswell, J.W., Plano Clark, V.L., Gutmann, M.L., Hanson, W.E., 2003. Advanced mixed methods research designs. In: Tashakkori, A., Teddlie, C. (Eds.), Handbook of Mixed Methods in Social and Behavioral Research. Sage, Thousand Oaks, pp. 209–240.
Dacko, S.G., Spalteholz, C., 2014. Upgrading the city: enabling intermodal travel behaviour. Technol. Forecast. Soc. Change 89, 222–235. https://doi.org/10.1016/j.techfore.2013.08.039.
Davis, F.D., 1989. Perceived usefulness, perceived ease of use, and user acceptance of information technology. Manag. Inf. Syst. Q. 13 (3), 319–340. https://doi.org/10.2307/249008.
Davis, F.D., Bagozzi, R.P., Warshaw, P.R., 1989. User acceptance of computer technology: a comparison of two theoretical models. Manag. Sci. 35 (8), 982–1003. https://doi.org/10.1287/mnsc.35.8.982.
Davis, F.D., Bagozzi, R.P., Warshaw, P.R., 1992. Extrinsic and intrinsic motivation to use computers in the workplace. J. Appl. Soc. Psychol. 22 (14), 1111–1132. https://doi.org/10.1111/j.1559-1816.1992.tb00945.x.
Daziano, R.A., Sarrias, M., Leard, B., 2017. Are consumers willing to pay to let cars drive for them? Analyzing response to autonomous vehicles. Transp. Res. Part C Emerg. Technol. 78, 150–164. https://doi.org/10.1016/j.trc.2017.03.003.
Dikmen, M., Burns, C.M., 2016. Autonomous driving in the real world: experiences with Tesla autopilot and summon. In: Proceedings of the 8th International Conference on Automotive User Interfaces and Interactive Vehicular Applications. Ann Arbor. pp. 225–228. https://doi.org/10.1145/3003715.3005465.
Dillon, A., Morris, M.G., 1996. User acceptance of information technology: theories and models. Annu. Rev. Inf. Sci. 14 (4), 3–32. http://hdl.handle.net/10150/105584.
Distler, V., Lallemand, C., Bellet, T., 2018. Acceptability and acceptance of autonomous mobility on demand: the impact of an immersive experience. In: Proceedings of the Conference on Human Factors in Computing Systems, https://doi.org/10.1145/3173574.3174186. April, 1–10.
Djamasbi, S., Fruhling, A.L., Loiacono, E., 2009. The influence of affect, attitude and usefulness in the acceptance of telemedicine systems. J. Inf. Technol. Theory Appl. 10 (1), 1–38.
Dodds, W.B., Monroe, K.B., Grewal, D., 1991. Effects of price, brand, and store information on buyers' product evaluations. J. Mark. Res. 28 (3), 307–319. https://doi.org/10.2307/3172866.
Douma, F., Palodichuk, S.A., 2012. Criminal liability issues created by autonomous vehicles. Santa Clara Law Rev. 52 (4), 1157–1169.
Eckoldt, K., Knobel, M., Hassenzahl, M., Schumann, J., 2012. An experiential perspective on advanced driver assistance systems. Inf. Technol. 54 (4), 165–171. https://doi.org/10.1524/itit.2012.0678.
Eugensson, A., Brännström, M., Frasher, D., Rothoff, M., Solyom, S., Robertsson, A., 2013. Environmental, safety, legal and societal implications of autonomous driving systems. In: Proceedings of the 23rd International Technical Conference on the Enhanced Safety of Vehicles (ESV). Seoul. pp. 1–15.
Fagan, M.H., Neill, S., Wooldridge, B.R., 2008. Exploring the intention to use computers: an empirical investigation of the role of intrinsic motivation, extrinsic motivation, and perceived ease of use. J. Comput. Inf. Syst. 48 (3), 31–37. https://doi.org/10.1080/08874417.2008.11646019.
Fagnant, D.J., Kockelman, K., 2015. Preparing a nation for autonomous vehicles: opportunities, barriers and policy recommendations. Transp. Res. Part A Policy Pract. 77, 167–181. https://doi.org/10.1016/j.tra.2015.04.003.
Fagnant, D.J., Kockelman, K., 2018. Dynamic ride-sharing and fleet sizing for a system of shared autonomous vehicles in Austin, Texas. Transp. Amst. 45 (1), 143–158. https://doi.org/10.1007/s11116-016-9729-z.
Fishman, E., Davies, L., 2016. Road user pricing: driverless cars, congestion and policy responses. In: Proceedings of the Australasian Transport Research Forum. Melbourne. pp. 1–17.
Fornell, C., Larcker, D.F., 1981. Evaluating structural equation models with unobservable variables and measurement error. J. Mark. Res. 18 (1), 39–50. https://doi.org/10.2307/3151312.
Fraedrich, E., Lenz, B., 2016. Societal and individual acceptance of autonomous driving. In: Maurer, M., Gerdes, J.C., Lenz, B., Winner, H. (Eds.), Autonomous Driving. Springer, Berlin, Heidelberg, pp. 621–640. https://doi.org/10.1007/978-3-662-48847-8_29.
Furneaux, B., Nevo, D., 2008. Beyond cognitions: a call for greater consideration of emotion in information systems decision theories. In: Proceedings of the 41st Annual Hawaii International Conference on System Sciences. Waikoloa, Hawaii. pp. 1–8. https://doi.org/10.1109/HICSS.2008.72.
Gkartzonikas, C., Gkritza, K., 2019. What have we learned? A review of stated preference and choice studies on autonomous vehicles. Transp. Res. Part C Emerg. Technol. 98, 323–337. https://doi.org/10.1016/j.trc.2018.12.003.
Glancy, D.J., 2012. Privacy in autonomous vehicles. Santa Clara Law Rev. 52, 1171–1239.
Grant-Muller, S., Usher, M., 2014. Intelligent transport systems: the propensity for environmental and economic benefits. Technol. Forecast. Soc. Change 82, 149–166. https://doi.org/10.1016/j.techfore.2013.06.010.
Greenblatt, J.B., Shaheen, S., 2015. Automated vehicles, on-demand mobility, and environmental impacts. Curr. Sustain. Renew. Energy Rep. 2 (3), 74–81. https://doi.org/10.1016/j.techfore.2013.06.010.
Grimsley, M., Meehan, A., 2007. e-Government information systems: evaluation-led design for public value and client trust. Eur. J. Inf. Syst. 16, 134–148. https://doi.org/10.1057/palgrave.ejis.3000674.
Haboucha, C.J., Ishaq, R., Shiftan, Y., 2017. User preferences regarding autonomous vehicles. Transp. Res. Part C Emerg. Technol. 78, 37–49. https://doi.org/10.1016/j.trc.2017.01.010.
Hacker, P., 2017. Personal data, exploitative contracts, and algorithmic fairness: autonomous vehicles meet the internet of things. Int. Data Priv. Law 7 (4), 266–286. https://doi.org/10.1093/idpl/ipx014.
Hair, J.F., Ringle, C.M., Sarstedt, M., 2011. PLS-SEM: indeed a silver bullet. J. Mark. Theory Pract. 19 (2), 139–151. https://doi.org/10.2753/MTP1069-6679190202.
Hair, J.F., Sarstedt, M., Ringle, C.M., Mena, J.A., 2012. An assessment of the use of partial least squares structural equation modeling in marketing research. J. Acad. Mark. Sci. 40 (3), 414–433. https://doi.org/10.1007/s11747-011-0261-6.
Hallgren, K.A., 2012. Computing inter-rater reliability for observational data: an overview and tutorial. Tutor. Quant. Methods Psychol. 8 (1), 23–34.
Hegner, S.M., Beldad, A.D., Brunswick, G.J., 2019. In automatic we trust: investigating the impact of trust, control, personality characteristics, and extrinsic and intrinsic motivations on the acceptance of autonomous vehicles. Int. J. Hum. Comput. Interact. 1–12. https://doi.org/10.1080/10447318.2019.1572353.
Heiskanen, E., Hyvönen, K., Niva, M., 2007. User involvement in radical innovation: are consumers conservative? Eur. J. Innov. Manag. 10 (4), 489–509. https://doi.org/10.1108/14601060710828790.
Hengstler, M., Enkel, E., Duelli, S., 2016. Applied artificial intelligence and trust: the case of autonomous vehicles and medical assistance devices. Technol. Forecast. Soc. Change 105, 105–120. https://doi.org/10.1016/j.techfore.2015.12.014.
Hevelke, A., Nida-Ruemelin, J., 2015. Responsibility for crashes of autonomous vehicles: an ethical analysis. Sci. Eng. Ethics 21 (3), 619–630. https://doi.org/10.1007/s11948-014-9565-5.
Hoff, K.A., Bashir, M., 2015. Trust in automation: integrating empirical evidence on factors that influence trust. Hum. Fact. J. Hum. Fact. Ergon. Soc. 57 (3), 407–434. https://doi.org/10.1177/0018720814547570.
Hohenberger, C., Spörrle, M., Welpe, I.M., 2017. Not fearless, but self-enhanced: the effects of anxiety on the willingness to use autonomous cars depend on individual levels of self-enhancement. Technol. Forecast. Soc. Change 116, 40–52. https://doi.org/10.1016/j.techfore.2016.11.011.
Hopf, C., 2004. Qualitative interviews: an overview. In: Flick, U., Kardorff, E.V., Steinke, I. (Eds.), A Companion to Qualitative Research. Sage Publications, Thousand Oaks, pp. 203–208.
Howard, D., Dai, D., 2014. Public perceptions of self-driving cars: the case of Berkeley, California. In: Proceedings of the 93rd Annual Meeting of the Transportation Research Board. Washington D.C.. pp. 1–21.
Hudson, J., Orviska, M., Hunady, J., 2019. People's attitudes to autonomous vehicles. Transp. Res. Part A Policy Pract. 121, 164–176. https://doi.org/10.1016/j.tra.2018.08.018.
Hulse, L.M., Xie, H., Galea, E.R., 2018. Perceptions of autonomous vehicles: relationships with road users, risk, gender and age. Saf. Sci. 102, 1–13. https://doi.org/10.1016/j.ssci.2017.10.001.
Hurt, H.T., Joseph, K., Cook, C.D., 1977. Scales for the measurement of innovativeness. Hum. Commun. Res. 4 (1), 58–65. https://doi.org/10.1111/j.1468-2958.1977.tb00597.x.
Kaur, K., Rampersad, G., 2018. Trust in driverless cars: investigating key factors influencing the adoption of driverless cars. J. Eng. Technol. Manag. 48, 87–96. https://doi.org/10.1016/j.jengtecman.2018.04.006.
Karahanna, E., Agarwal, R., Angst, C.M., 2006. Reconceptualizing compatibility beliefs in technology acceptance research. Manag. Inf. Syst. Q. 30 (4), 781–804. https://doi.org/10.2307/25148754.
King, W.R., He, J., 2006. A meta-analysis of the technology acceptance model. Inf. Manag. 43 (6), 740–755. https://doi.org/10.1016/j.im.2006.05.003.
Kilian-Yasin, K., Wöhr, M., Tangour, C., Fournier, G., 2016. Social acceptance of alternative mobility systems in Tunis. Transp. Res. Procedia 19, 135–146. https://doi.org/10.1016/j.trpro.2016.12.074.
Kleijnen, M., Lee, N., Wetzels, M., 2009. An exploration of consumer resistance to innovation and its antecedents. Journal of Economic Psychology 30 (3), 377–392.
Koerber, M., Bengler, K., 2014. Potential individual differences regarding automation effects in automated driving. In: Proceedings of the XV International Conference on Human Computer Interaction. Tenerife. pp. 1–7. https://doi.org/10.1145/2662253.2662275.
Kohl, C., Mostafa, D., Boehm, M., Krcmar, H., 2017. Disruption of individual mobility ahead? A longitudinal study of risk and benefit perceptions of self-driving cars on Twitter. In: Proceedings of the 13th International Conference on Wirtschaftsinformatik. St. Gallen. pp. 1220–1232.
Koo, J., Shin, D., Steinert, M., Leifer, L., 2016. Understanding driver responses to voice alerts of autonomous car operations. Int. J. Veh. Des. 70 (4), 377–392. https://doi.org/10.1504/IJVD.2016.076740.
König, M., Neumayr, L., 2017. Users' resistance towards radical innovations: the case of the self-driving car. Transp. Res. Part F Traffic Psychol. Behav. 44, 42–52. https://doi.org/10.1016/j.trf.2016.10.013.
Koscher, K., Czeskis, A., Roesner, F., Patel, S., Kohno, T., Checkoway, S., McCoy, D., Kantor, B., Anderson, D., Shacham, H., Savage, S., 2010. Experimental security analysis of a modern automobile. In: Proceedings of the IEEE Symposium on Security and Privacy. San Diego, California. pp. 447–462. https://doi.org/10.1109/SP.2010.34.
Koul, S., Eydgahi, A., 2018. Utilizing technology acceptance model (TAM) for driverless car technology adoption. J. Technol. Manag. Innov. 13 (4), 37–46. https://doi.org/10.4067/S0718-27242018000400037.
Kulviwat, S., Burner II, G.C., Kumar, A., Nasco, S.A., Clark, T., 2007. Toward a unified theory of consumer acceptance technology. Psychol. Mark. 24 (12), 1059–1084. https://doi.org/10.1002/mar.20196.
Kuo, Y.-.F., Yen, S.-.N., 2009. Towards an understanding of the behavioral intention to use 3G mobile value-added services. Comput. Hum. Behav. 25 (1), 103–110. https://doi.org/10.1016/j.chb.2008.07.007.
Kyriakidis, M., Happee, R., De Winter, J.C.F., 2015. Public opinion on automated driving: results of an international questionnaire among 5,000 respondents. Transp. Res. Part F Traffic Psychol. Behav. (32), 127–140. https://doi.org/10.1016/j.trf.2015.04.014.
Lee, Y., Kozar, K.A., Larsen, K.R.T., 2003. The technology acceptance model: past, present, and future. Commun. Assoc. Inf. Syst. 12 (1), 752–780. https://doi.org/10.4067/S0718-27242018000400037.
Lee, J., Lee, D., Park, Y., Lee, S., Ha, T., 2019. Autonomous vehicles can be shared, but a

feeling of ownership is important: examination of the influential factors for intention to use autonomous vehicles. Transp. Res. Part C Emerg. Technol. 107, 411–422. https://doi.org/10.1016/j.trc.2019.08.020.
Leicht, T., Chtourou, A., Ben Youssef, K., 2018. Consumer innovativeness and intentioned autonomous car adoption. J. High Technol. Manag. Res. 29, 1–11. https://doi.org/10.1016/j.hitech.2018.04.001.
Liljamo, T., Liimatainen, H., Pöllänen, M., 2018. Attitudes and concerns on automated vehicles. Transp. Res. Part F Traffic Psychol. Behav. 59, 24–44. https://doi.org/10.1016/j.trf.2018.08.010.
Lin, H.F., 2011. An empirical investigation of mobile banking adoption: the effect of innovation attributes and knowledge-based trust. Int. J. Inf. Manag. 31 (3), 252–260. https://doi.org/10.1016/j.ijinfomgt.2010.07.006.
Lin, C.-.H., Shih, H.-.Y., Sher, P.J., 2007. Integrating technology readiness into technology acceptance: the tram model. Psychol. Mark. 24 (7), 641–657. https://doi.org/10.1002/mar.20177.
Liu, P., Yang, R., Xu, Z., 2018. Public acceptance of fully automated driving: effects of social trust and risk/benefit perceptions. Risk Anal. 39, 326–341. https://doi.org/10.1111/risa.13143.
Lohmoeller, J.-.B., 1989. Latent Variable Path Modeling With Partial Least Squares. Physica, Heidelberg.
Madigan, R., Louw, T., Wilbrink, M., Schieben, A., Merat, N., 2017. What influences the decision to use automated public transport? Using UTAUT to understand public acceptance of automated road transport systems. Transp. Res. Part F Traffic Psychol. Behav. 50, 55–64. https://doi.org/10.1016/j.trf.2017.07.007.
Mahoney III, W.P., O'Sullivan, J.M., 2013. Realizing the potential of vehicle-based observations. Bull. Am. Meteorol. Soc. 94 (7), 1007–1018. https://doi.org/10.1175/BAMS-d-12-00044.1.
Manfreda, A., Ljubi, K., Groznik, A., 2019. Autonomous vehicles in the smart city era: an empirical study of adoption factors important for millennials. Int. J. Inf. Manag. 1–12. https://doi.org/10.1016/j.ijinfomgt.2019.102050.
Mao, E., Srite, M., Thatcher, J.B., Yaprak, O., 2005. A research model for mobile phone service behaviours: empirical validation in the U.S. and Turkey. J. Global Inf. Technol. Manag. 8 (4), 7–28. https://doi.org/10.1080/1097198X.2005.10856406.
Marletto, G., 2019. Who will drive the transition to self-driving? A socio-technical analysis of the future impact of automated vehicles. Technol. Forecast. Soc. Change 139, 1–14. https://doi.org/10.1016/j.techfore.2018.10.023.
Mayring, P., 2014. Qualitative Content Analysis: Theoretical Foundation, Basic Procedures and Software Solution. SSOAR Available at: https://www.ssoar.info/ssoar/bitstream/handle/document/39517/ssoar-2014-mayring-Qualitative_content_analysis_theoretical_foundation.pdf?sequence=1&isAllowed=y&lnkname=ssoar-2014-mayring-Qualitative_content_analysis_theoretical_foundation.pdf.
Merfeld, K., Wilhelms, M.P., Henkel, S., 2019a. Being driven autonomously: a qualitative study to elicit consumers' overarching motivational structures. Transp. Res. Part C Emerg. Technol. 107, 229–247. https://doi.org/10.1016/j.trc.2019.08.007.
Merfeld, K., Wilhelms, M.P., Henkel, S., Kreutzer, K., 2019b. Carsharing with shared autonomous vehicles: uncovering drivers, barriers and future developments: a four-stage Delphi study. Technol. Forecast. Soc. Change 144, 66–81. https://doi.org/10.1016/j.techfore.2019.03.012.
Meyer, J., Becker, H., Boesch, P.M., Axhausen, K.W., 2017. Autonomous vehicles: the next jump in accessibilities? Res. Transp. Econ. 62, 80–91. https://doi.org/10.1016/j.retrec.2017.03.005.
Midgley, D.F., Dowling, G.R., 1993. A longitudinal study of product form innovation: the interaction between predispositions and social messages. J. Consum. Res. 19, 611–625. https://doi.org/10.1086/209326.
Moore, G.C., Benbasat, I., 1991. Development of an instrument to measure the perceptions of adopting an information technology innovation. Inf. Syst. Res. 2 (3), 192–222. https://doi.org/10.1287/isre.2.3.192.
Motamedi, S., Wang, P., Zhang, T., Chan, C.Y., 2020. Acceptance of full driving automation: personally owned and shared-use concepts. Hum. Factors 62 (2), 288–309. https://doi.org/10.1177/0018720819870658.
Nasri, W., Charfeddine, L., 2012. Factors affecting the adoption of Internet banking in Tunisia: an integration theory of acceptance model and theory of planned behaviour. J. High Technol. Manag. Res. 23, 1–14. https://doi.org/10.1016/j.hitech.2012.03.001.
Nees, M.A., 2016. Acceptance of self-driving cars: an examination of idealized versus realistic portrayals with a self-driving car acceptance scale. Proc. Hum. Factors Ergon. Soc. Annu. Meet. 60 (1), 1449–1453. https://doi.org/10.1177/1541931213601332.
Nieuwenhuijsen, J., de Almeida Correia, G.H., Milakis, D., van Arem, B., van Daalen, E., 2018. Towards a quantitative method to analyze the long-term innovation diffusion of automated vehicles technology using system dynamics. Transp. Res. Part C Emerg. Technol. 86, 300–327. https://doi.org/10.1016/j.trc.2017.11.016.
Nishihori, Y., Kimura, K., Taniguchi, A., Morikawa, T., 2020. What affects social acceptance and use intention for autonomous vehicles–benefits, risk perception, or experience? Meta-analysis in Japan. Int. J. Intell. Transp. Syst. Res. 18 (1), 22–34. https://doi.org/10.1007/s13177-018-0170-x.
Nordhoff, S., van Arem, B., Happee, R., 2016. A conceptual model to explain, predict, and improve user acceptance of driverless vehicles. Transp. Res. Rec. J. Transp. Res. Board 2602 (1), 60–67. https://doi.org/10.3141/2F2602-08.
Nordhoff, S., de Winter, J., Kyriakidis, M., van Arem, B., Happee, R., 2018. Acceptance of driverless vehicles: results from a large cross-national questionnaire study. J. Adv. Transp. 2018, 1–22. https://doi.org/10.1155/2018/5382192.
Nordhoff, S., de Winter, J., Payre, W., van Arem, B., Happee, R., 2019. What impressions do users have after a ride in an automated shuttle? An interview study. Transp. Res. Part F Traffic Psychol. Behav. 63, 252–269. https://doi.org/10.1016/j.trf.2019.04.009.
Nunez, M., 2017. My First Ride in a Self-driving Car was Harrowing as Hell. GIZMODO Available at: https://www.gizmodo.com.au/2017/01/my-first-ride-in-a-self-driving-car-was-harrowing-as-hell/.
Nysveen, H., Pedersen, P.E., Thorbjørnsen, H., 2005. Intention to use mobile services: antecedents and cross-services comparisons. J. Acad. Mark. Sci. 33 (3), 330–346. https://doi.org/10.1177/0092070305276149.
O'Toole, R., 2014. Policy implications of autonomous vehicles. Policy Anal. 758, 1–16.
Pai, F.-.Y., Huang, K.-.I., 2011. Applying the technology acceptance model to the introduction of healthcare information systems. Technol. Forecast. Soc. Change 78, 650–660. https://doi.org/10.1016/j.techfore.2010.11.007.
Panagiotopoulos, I., Dimitrakopoulos, G., 2018. An empirical investigation on consumers' intentions towards autonomous driving. Transp. Res. Part C Emerg. Technol. 95, 773–784. https://doi.org/10.1016/j.trc.2018.08.013.
Paddeu, D., Parkhurst, G., Shergold, I., 2020. Passenger comfort and trust on first-time use of a shared autonomous shuttle vehicle. Transp. Res. Part C Emerg. Technol. 115, 102604. https://doi.org/10.1016/j.trc.2020.02.026.
Parasuraman, A., 2000. Technology readiness index (Tri): a multiple-item scale to measure readiness to embrace new technologies. J. Serv. Res. 2 (4), 307–320. https://doi.org/10.1177/109467050024001.
Patti, F.P., 2019. The European road to autonomous vehicles. Fordham Int. Law J. 43 (1), 125–162.
Pattinson, J.A., Chen, H., 2020. A barrier to innovation: Europe's ad-hoc cross-border framework for testing prototype autonomous vehicles. Int. Rev. Law Comput. Technol. 34 (1), 108–122. https://doi.org/10.1080/13600869.2019.1696651.
Payre, W., Cestac, J., Delhomme, P., 2014. Intention to use a fully automated car: attitudes and a priori acceptability. Transp. Res. Part F 27, 252–263. https://doi.org/10.1016/j.trf.2014.04.009.
Pedersen, P.E., 2005. Adoption of mobile internet services: an exploratory study of mobile commerce early adopters. J. Organ. Comput. Electron. Commer. 15 (2), 203–222. https://doi.org/10.1207/s15327744joce1503_2.
Penmetsa, P., Adanu, E.K., Wood, D., Wang, T., Jones, S.L., 2019. Perceptions and expectations of autonomous vehicles: a snapshot of vulnerable road user opinion. Technol. Forecast. Soc. Change 143, 9–13. https://doi.org/10.1016/j.techfore.2019.02.010.
Peters, A., Popp, M., Agosti, R., Ryf, B., 2011. Electric mobility: a survey of different consumer groups in Germany with regard to adoption. ECEEE Summer Study 2 (3), 983–994. http://publica.fraunhofer.de/documents/N-183480.html.
Phelps, J., Nowak, G., Ferrell, E., 2000. Privacy concerns and consumer willingness to provide personal information. J. Public Policy Mark. 191, 27–41. https://doi.org/10.1509/jppm.19.1.27.16941.
Planing, P., 2014. Innovation Acceptance: The Case of Advanced Driver-assistance Systems. Springer Fachmedien, Wiesbaden.
Podsakoff, P.M., MacKenzie, S.B., Lee, J.Y., Podsakoff, N.P., 2003. Common method biases in behavioural research: a critical review of the literature and recommended remedies. J. Appl. Psychol. 88 (5), 879–903. https://doi.org/10.1037/0021-9010.88.5.879.
Potter, W.J., Levine-Donnerstein, D., 1999. Rethinking validity and reliability in content analysis. J. Appl. Commun. Res. 27, 258–284. https://doi.org/10.1080/00909889909365539.
Rahman, M.M., Lesch, M.F., Horrey, W.J., Strawderman, L., 2017. Assessing the utility of TAM, TPB, and UTAUT for advanced driver assistance systems. Accident Anal. Prev. 108, 361–373. https://doi.org/10.1016/j.aap.2017.09.011.
Rahman, M., Deb, S., Strawderman, L., Burch, R., Smith, B., 2019. How the older population perceives self-driving vehicles. Transp. Res. Part F Psychol. Behav. 65, 242–257. https://doi.org/10.1016/j.iatssr.2019.05.004.
Reese, H., 2016. Updated: Autonomous Driving Levels 0 to 5: Understanding the Differences. Available at: https://www.techrepublic.com/article/autonomous-driving-levels-0-to-5-understanding-the-differences/.
Roberts, J.A., 1995. Profiling Levels of Socially Responsible Consumer Behavior: A Cluster Analytic Approach and Its Implications for Marketing. Journal of Marketing Theory and Practice 3 (4), 97–117.
Ro, Y., Ha, Y., 2019. A factor analysis of consumer expectations for autonomous cars. J. Comput. Inf. Syst. 59, 52–60. https://doi.org/10.1080/08874417.2017.1295791.
Roedel, C., Stadler, S., Meschtscherjakov, A., Tscheligi, M., 2014. Towards autonomous cars: the effect of autonomy levels on acceptance and user experience. In: Proceedings of the 6th International Conference on Automotive User Interfaces and Interactive Vehicular Applications. Seattle. pp. 1–8. https://doi.org/10.1145/2667317.2667330.
Rogers, E.M., 2003. Diffusion of Innovations, 5th edition. Free Press, New York.
Saadé, R., Bahli, B., 2005. The impact of cognitive absorption on perceived usefulness and perceived ease of use in on-line learning: an extension of the technology acceptance model. Inf. Manag. 422, 317–327. https://doi.org/10.1016/j.im.2003.12.013.
SAE International, 2018. Taxonomy and Definitions for Terms Related to Driving Automation Systems for On-road Motor Vehicles: J3016_201806. Available at: https://www.sae.org/standards/content/j3016_201806/.
Schepers, J., Wetzels, M., 2007. A meta-analysis of the technology acceptance model: investigating subjective norm and moderation effects. Inf. Manag. 44 (1), 90–103. https://doi.org/10.1016/j.im.2006.10.007.
Schoettle, B., Sivak, M., 2014. A survey of public opinion about connected vehicles in the US, the UK, and Australia. In: Proceedings of the International Conference on Connected Vehicles and Expo (ICCVE). IEEE, pp. 687–692. https://doi.org/10.1109/ICCVE.2014.7297637.
Schweitzer, N., Hofmann, R., Meinheit, A., 2019. Strategic customer foresight: from research to strategic decision-making using the example of highly automated vehicles. Technol. Forecast. Soc. Change 144, 49–65. https://doi.org/10.1016/j.techfore.2019.04.004.
Shabanpour, R., Mousavi, S.N.D., Golshani, N., Auld, J., Mohammadian, A., 2017. Consumer preferences of electric and automated vehicles. In: Proceedings of the 5th IEEE International Conference on Models and Technologies for Intelligent Transportation Systems (MT-ITS). Naples. pp. 716–720. https://doi.org/10.1109/MTITS.2017.8005606.
Shanker, R., Jonas, A., Devitt, S., Huberty, K., Flannery, S., Greene, W., Swinburne, B., Locraft, G., Wood, A., Weiss, K., Moore, J., Schenker, A., Jain, P., Ying, Y., Kakiuchi, S., Hoshino, R., Humphrey, A., 2013. Autonomous Cars: Self-Driving the New Auto Industry Paradigm. Morgan Stanley Blue Paper.

Shariff, A., Bonnefon, J.F., Rahwan, I., 2017. Psychological roadblocks to the adoption of self-driving vehicles. Nat. Hum. Behav. 1 (10), 694–696. https://doi.org/10.1038/s41562-017-0202-6.

Skeete, J.P., 2018. Level 5 autonomy: the new face of disruption in road transport. Technol. Forecast. Soc. Change 134, 22–34. https://doi.org/10.1016/j.techfore.2018.05.003.

Solove, D.J., 2006. A taxonomy of privacy. Univ. PA Law Rev. 154 (3), 477–564.

Sun, H., Zhang, P., 2006. Causal relationships between perceived enjoyment and perceived ease of use: an alternative approach. J. Assoc. Inf. Syst. 7 (9), 618–645.

Talebian, A., Mishra, S., 2018. Predicting the adoption of connected autonomous vehicles: a new approach based on the theory of diffusion of innovations. Transp. Res. Part C Emerg. Technol. 95, 363–380. https://doi.org/10.1016/j.trc.2018.06.005.

Taylor, S., Todd, P.A., 1995. Understanding information technology usage: a test of competing models. Inf. Syst. Res. 6 (2), 144–176. https://doi.org/10.1287/isre.6.2.144.

Teo, T.S.H., Pok, S.H., 2003. Adoption of WAP-enabled mobile phones among internet users. Omega Int. J. Manag. Sci. 31 (6), 483–498. https://doi.org/10.1016/j.omega.2003.08.005.

Taeihagh, A., Lim, H.S.M., 2019. Governing autonomous vehicles: emerging responses for safety, liability, privacy, cybersecurity, and industry risks. Transp. Rev. 39 (1), 103–128. https://doi.org/10.1080/01441647.2018.1494640.

Thatcher, J.B., McKnight, D., Baker, E.W., Arsal, R.E., Roberts, N.H., 2011. The role of trust in postadoption it exploration: an empirical examination of knowledge management systems. IEEE Trans. Eng. Manag. 58, 56–70. https://doi.org/10.1109/TEM.2009.2028320.

Thomopoulos, N., Givoni, M., 2015. The autonomous car—a blessing or a curse for the future of low carbon mobility? An exploration of likely vs. desirable outcomes. Eur. J. Futures Res. 3 (1), 1–11. https://doi.org/10.1007/s40309-015-0071-z.

Thongmak, M., 2012. Green ICTs? Awareness and adoption: a case study of university freshmen in Thailand. Eur. Conf. Inf. Syst. 1–14.

Thorpe, J., Motwani, E., 2017. Nudging People Into Autonomous Vehicles. PricewaterhouseCoopers Technical report.

Tornatzky, L.G., Klein, K.J., 1982. Innovation characteristics and innovation adoption-implementation: a meta-analysis of findings. IEEE Trans. Eng. Manag. 29 (1), 28–45. https://doi.org/10.1109/TEM.1982.6447463.

Varghese, J.Z., Boone, R.G., 2015. Overview of autonomous vehicle sensors and systems. In: Proceedings of the International Conference on Operations Excellence and Service Engineering. Florida. pp. 178–191.

Vellinga, N.E., 2017. From the testing to the deployment of self-driving cars: legal challenges to policymakers on the road ahead. Comput. Law Secur. Rev. 33 (6), 847–863. https://doi.org/10.1016/j.clsr.2017.05.006.

Venkatesh, V., 2000. Determinants of perceived ease of use: integrating control, intrinsic motivation, and emotion into the technology acceptance model. Inf. Syst. Res. 11 (4), 342–365. https://doi.org/10.1287/isre.11.4.342.11872.

Venkatesh, V., Bala, H., 2008. Technology acceptance model 3 and a research agenda on interventions. Decis. Sci. 39 (2), 273–315. https://doi.org/10.1111/j.1540-5915.2008.00192.x.

Venkatesh, V., Brown, S.A., Bala, H., 2013. Bridging the qualitative-quantitative divide: guidelines for conducting mixed methods research in information systems. MIS Q. 37 (1), 21–54. https://doi.org/10.25300/MISQ/2013/37.1.02.

Venkatesh, V., Morris, M.G., Davis, G.B., Davis, F.D., 2003. User acceptance of information technology: toward a unified view. MIS Q. 27 (3), 425–478. https://doi.org/10.2307/30036540.

Venkatesh, V., Davis, F.D., 2000. A theoretical extension of the technology acceptance model: four longitudinal field studies. Manag. Sci. 46 (2), 186–204. https://doi.org/10.1287/mnsc.46.2.186.11926.

Venkatesh, V., Davis, F.D., Morris, M.G., 2007. Dead or alive? The development, trajectory and future of technology adoption research. J. Assoc. Inf. Syst. 8 (4), 267–286. https://doi.org/10.17705/1jais.00120.

Venkatesh, V., Thong, J.Y.L., Xu, X., 2012. Consumer acceptance and use of information technology: extending the unified theory of acceptance and use of technology. Manag. Inf. Syst. Q. 36 (1), 157–178. https://doi.org/10.2307/41410412.

Vijayasarathy, L.R., 2004. Predicting consumer intentions to use on-line shopping: the case for an augmented technology acceptance model. Inf. Manag. 41 (6), 747–762. https://doi.org/10.1016/j.im.2003.08.011.

Xu, Z., Zhang, K., Min, H., Wang, Z., Zhao, X., Liu, P., 2018. What drives people to accept automated vehicles? Findings from a field experiment. Transp. Res. Part C Emerg. Technol. 95, 320–334. https://doi.org/10.1016/j.trc.2018.07.024.

Waymo, 2018. On the Road. Available at: https://waymo.com/journey/.

Wu, P.F., 2012. A mixed methods approach to technology acceptance research. J. Assoc. Inf. Syst. 13 (13), 172–187.

Wu, J.H., Wang, S.C., 2005. What drives mobile commerce? An empirical evaluation of the revised technology acceptance model. Inf. Manag. 42 (5), 719–729. https://doi.org/10.1016/j.im.2004.07.001.

Wu, J., Liao, H., Wang, J.W., Chen, T., 2019. The role of environmental concern in the public acceptance of autonomous electric vehicles: a survey from China. Transp. Res. Part F Traffic Psychol. Behav. 60, 37–46. https://doi.org/10.1016/j.trf.2018.09.029.

Yang, H.D., Yoo, Y., 2004. It's all about attitude: revisiting the technology acceptance model. Decis. Support Syst. 38 (1), 19–31. https://psycnet.apa.org/doi/10.1016/S0167-9236(03)00062-9.

Yuen, K.F., Wong, Y.D., Ma, F., Wang, X., 2020. The determinants of public acceptance of autonomous vehicles: an innovation diffusion perspective. J. Clean. Prod., 121904. https://doi.org/10.1016/j.jclepro.2020.121904.

Zeithaml, V.A., 1988. Consumer perceptions of price, quality, and value: a means–end model and synthesis of evidence. J. Mark. 52 (3), 2–22. https://doi.org/10.2307/1251446.

Zhang, T., Tao, D., Qu, X., Zhang, X., Lin, R., Zhang, W., 2019. The roles of initial trust and perceived risk in public's acceptance of automated vehicles. Transp. Res. Part C 98, 207–220. https://doi.org/10.1016/j.trc.2018.11.018.

Zhang, T., Tao, D., Qu, X., Zhang, X., Zeng, J., Zhu, H., 2020. Automated vehicle acceptance in China: social influence and initial trust are key determinants. Transp. Res. Part C 112, 220–233. https://doi.org/10.1016/j.trc.2020.01.027.

Zmud, J.P., Sener, I.N., Wagner, J., 2017. Towards an understanding of the travel behavior impact of autonomous vehicles. Transp. Res. Procedia 25, 2500–2519. https://doi.org/10.1016/j.trpro.2017.05.281.

Ilja Nastjuk Dr. Ilja Nastjuk is an associate member of the Smart Mobility Research Group (SMRG) at the Georg-August-University of Goettingen, Germany. He received a Ph.D. degree in Information Systems from the University of Göttingen and a Ph.D. degree in Accounting and Corporate Governance from Macquarie University. His-research interests span the influence of technology on stress and human behavior. His-work has been published in a number of peer-reviewed journals and conference proceedings, including Transportation Research Part D: Transport and Environment, Transportation Research Part F: Traffic Psychology, or International Conference on Information Systems

Bernd Herrenkind Bernd Herrenkind is a consultant at MHP - A Porsche Company in the area of Mobility Services and Strategy. He is a member of the Smart Mobility Research Group (SMRG) at the University of Goettingen. Since 2018, Bernd has been pursuing his Doctorate in Information Systems at the University of Goettingen. His-research focuses on technology acceptance, autonomous driving, and mobility services. His-work has been published in a number of peer-reviewed journals and conference proceedings, including Transportation Research Part D: Transport and Environment, HMD Praxis der Wirtschaftsinformatik or Internationale Tagung Wirtschaftsinformatik.

Mauricio Marrone Dr. Mauricio Marrone is a lecturer of business information systems at Macquarie University in Sydney. He teaches information systems, with a focus on digital transformation and entrepreneurship. He researches innovative approaches to boost research productivity and find application opportunities in Information Systems and beyond. He has developed a text mining tool that helps academics find research gaps. His-work has been published in a number of peer-reviewed journals and conference proceedings, including Business and Information Systems Engineering, Communications of the Association for Information Systems, Transportation Research Part F: Traffic Psychology and Behaviour, or International Conference on Information Systems.

Alfred Benedikt Brendel Dr. Alfred Benedikt Brendel is a postdoctoral researcher and head of the Smart Mobility Research Group (SMRG) at the University of Goettingen. He holds a Doctor's degree in Business Information Systems from the University of Goettingen. His-research focuses on the design of IS-based solutions for vehicle supply and demand management in carsharing and shared autonomous vehicle services. His-work has been published in a number of peer-reviewed journals and conference proceedings, including Transportation Research Part D: Transport and Environment, International Conference on Information Systems, or European Conference on Information Systems.

Lutz M. Kolbe Since 2007, Prof. Dr. Lutz M. Kolbe is a full professor and Chair of Information Management at University of Goettingen, Germany. Lutz holds a Doctor's degree in Business Administration from the Technical University Freiberg, Germany. Subsequently, he led Deutsche Bank's IT think tanks in Frankfurt, New York, and San Francisco. His-main research interests are IT Innovation Management and Sustainable Information Management. His-work has been published in a number of peer-reviewed journals and conference proceedings, including Information Systems Journal, Energy Policy, MIS Quarterly Executive, or International Conference on Information Systems.
