# Supplementary material for: A modification of technology acceptance model for investigating driver-vehicle interaction systems usage
Source: PLoS One. 2025 Apr 22;20(4):e0322221. doi: 10.1371/journal.pone.0322221 (PMC12013904; doi:10.1371/journal.pone.0322221)
Supplement: Attachment — Submitted filename: Response to Reviewers.docx [file pone.0322221.s005.docx]

Dear Editor,

Thank you for your questions and comments.

We have carefully addressed your concerns as shown in this response letter and revised the manuscript.

Please check the “Revised Manuscript with Track Changes” for tracking our revisions. The major changes made in the revised manuscript have been highlighted in yellow (the deleted contents and revisions made on language problems are not included). We have also uploaded a file called “Revised Manuscript with the Whole Track Changes” which covers all the change histories.

Based on the reviewers’ comments I suggest you to cite the following articles and compare your findings to enhance its scope.

**Reply:** Thank you for the references, we have carefully read the references and added the citations in the paper to enhance the scope of our discussion of perceived usefulness and enjoyment. Please check lines 156 to 159, 171 to 173, and 543 to 547.

Journal requirements

1. Please ensure that your manuscript meets PLOS ONE's style requirements, including those for file naming.

**Reply:** We have checked and modified the manuscript and file names to meet PLOS ONE's style requirements following the links.

2. We note that you have indicated that there are restrictions to data sharing for this study. PLOS only allows data to be available upon request if there are legal or ethical restrictions on sharing data publicly.

**Reply:** We have uploaded the data on the “Dryad”, DOI: 10.5061/dryad.4qrfj6qm5.

Please let us know if you have any questions or further feedback.

I hope our revisions can satisfy your comments and questions.

Sincerely yours,

Dong and Itoh

Dear Reviewer #1,

Thank you for your questions and comments.

We have carefully addressed your concerns as shown in this response letter and revised the manuscript.

Please check the “Revised Manuscript with Track Changes” for tracking our revisions. The major changes made in the revised manuscript have been highlighted in yellow (the deleted contents and revisions made on language problems are not included). We have also uploaded a file called “Revised Manuscript with the Whole Track Changes” which covers all the change histories.

Abstract:
It is necessary to include the following items in this section:
- In the “Abstract” section; no information has been provided regarding the type of study, the number of participants, the tools/questionnaires used, and so on. The research findings should be presented quantitatively in the "Abstract" section. Additionally, a general conclusion from the study should be provided as the "Conclusion".

**Reply:** We have added the type of study, the details of the participants, and the information about the questionnaire used in the “Abstract” from lines 30 to 37. The general conclusion of the study has been added from lines 48 to 51.

- Please state the significance level (p-value) in which this analysis was performed in the results section.
**Reply:** We have stated the significant level at lines 40, 44, and 46.

Introduction:
- There is no need to introduce the three determinant constructs of TAM (as illustrated in Fig 1) in the “Introduction” section. It is recommended to present it in the "Materials and Methods" section under a subsection titled “Technical acceptance model”.

**Reply:** We have moved the introduction of TAM from the “Introduction” section to the “Materials and methods” section where a subsection called “TAM” was created at line 91.

- The authors need to revise and rewrite the text to improve coherence between the paragraphs.

**Reply:** We have deleted some parts of “Introduction” and added a paragraph from lines 84 to 87 to improve the coherence between the sections “Introduction” and “Materials and methods”.

Material and methods:
- In the “Materials and Methods” section, the authors should present the model constructs′ explanations more concisely and avoid providing irrelevant details. It is recommended to present these constructs in a table along with their definitions and corresponding references. This approach will improve the clarity of the components, enhance readability for the reader, and ultimately make the text more concise.

**Reply:** We have deleted the irrelevant details and modified the sentences to improve the coherence between the paragraphs. The modifications are highlighted in yellow. In addition, a section named “Proposed model” was added to include the subsections of the model constructs and added the general introduction from lines 150 to 153. Regarding the table of the constructs, we have introduced the definitions of the constructs and their references in Table 1 at line 238.

- Figure 1 and its explanation should be presented in this section, not in the "Introduction" section.

**Reply:** Figure 1 has been moved to the “TAM” subsection at line 101.

- Please organize the study hypotheses into a table.

**Reply**: We have added a new subsection named “Hypotheses” and put the study hypotheses into Table 2 at line 256.

- In the “Materials and Methods” section; the authors need to provide information on the validity and reliability of the questionnaire. Additionally, please specify whether the questionnaire was distributed to participants in English or Japanese. If it was translated into Japanese, was its validity verified?

**Reply:** Because this study is a continuation of a previous study [19] on questionnaire design, we had proved the questionnaire’s validity and reliability with exploratory factor analysis in that study. Regarding the language of the questionnaire, we had used Japanese language translated from Chinese language. We had invited a bilingualist who has expertise in these two languages to check the contents of the questionnaire. We have added these explanations from lines 278 to 283 in the subsection “Questionnaire design”.

- In “Data analysis” subsection; Was the assumption of normality of the data evaluated? If yes, with what test?

**Reply:** Because the data is based on the questionnaire survey, we had used the absolute value of skewness and kurtosis to check the normality. We have added the values of skewness and kurtosis in Table 3. Please check the sentences from lines 297 to 300 and Table 3 at line 301. In addition, we have changed the subsection title to “Measurement and data analysis” which might be more suitable.

- In “Data analysis” subsection; Were outliers checked in the study? At what confidence level was the data analysis conducted?

**Reply:** We have added the strategy of deleting invalid data from lines 294 to 297 and added the confidence level from lines 306 to 307.

Discussion:
- The authors have listed the study's limitations separately under the subsection “Limitations”. It is essential to include this information as a paragraph at the end of the “Discussion” section. It is necessary to express the strengths and limitations of the present study more clearly. Can the authors state more limitations for the present study? Also, the authors should provide their suggestions for future studies (if any). I believe the stated limitations are unclear and may be difficult for the reader to understand.

**Reply:** We have rewritten the subsection of “Limitations” to make it clearer for reading. We have added and discussed 5 limitations in the “Limitations” subsection: the period of respondent’s consideration, actual system use, perceived safety, regional characteristics, and the correlation between satisfaction and demographics. For each limitation, we have explained our continued works and future plans. Please check the “Limitations” subsection from lines 574 to 604.

Conclusions:
- Authors are expected to present a complete and adequate conclusion of their study in this section. In the “Conclusion” section, authors should present a general conclusion of the study, not the study findings.

**Reply:** We have rewritten the section “Conclusion” to provide general conclusion. Please check the section “Conclusion” from lines 606 to 621.

- Some of the study's limitations are mentioned in the “Conclusion” section. These limitations should be consolidated and presented together in the final paragraph of the “Discussion” section.

**Rely:** We have merged these sentences into the subsection of “Limitations”.

Please let us know if you have any questions or further feedback.

I hope our revisions can satisfy your comments and questions.

Sincerely yours,

Dong and Itoh

Dear Reviewer #2,

Thank you for your questions and comments.

We have carefully addressed your concerns as shown in this response letter and revised the manuscript.

Please check the “Revised Manuscript with Track Changes” for tracking our revisions. The major changes made in the revised manuscript have been highlighted in yellow (the deleted contents and revisions made on language problems are not included). We have also uploaded a file called “Revised Manuscript with the Whole Track Changes” which covers all the change histories.

1. The topic is very interesting and applicable to present day scenario.

**Reply:** Thank you for your interest in this paper.

2. I would request that, as this paper is a questionnaire based study, the author could discuss "the degree of automation accepted" and "Safety of Driver and Public" before discussing the HMI of Driver-Vehicle Interaction. These two aspects would also influence the user experience and perceived usefulness under Technology Acceptance Model. Authors would appreciate the fact that if the Drivers are completely satisfied about safety aspects of automated driving, then only they will be able to divert their attention (without increasing the cognitive workload) to execute Non-Driving Related Tasks (NDRT), like, playing a game or watching a movie (Refer to the two enclosed files).

**Reply:** Thank you for the attached papers that inspired our revisions. We agreed with your idea that the paper should discuss safety as well as the degree of automation accepted. Because we are researching the safety, usability, and user acceptance of our designed driver-vehicle interface prototype in the continued work of this paper currently. Thus, we have added the discussion of safety in the section of “Introduction” from lines 54 to 66 and discussed the limitation of safety in the subsection of “Limitations” from lines 587 to 592. Please check these paragraphs.

3. Sentences should never start with abbreviations (e.g., refer paragraph starting at line no. 68). Kindly correct it throughout the manuscript.

**Reply**: We have corrected the problem. We have also checked the manuscript to correct similar problems.

4. The sentences have to be meaningful. Please correct it throughout the manuscript. For example, refer line 88, "Several studies.. introduced TAM ... systems." Instead of the word 'introduced' the word 'used' would be giving the meaning in better way. Logically several studies cannot introduce something. It is understood that for the first time something is introduced and after that it is used.

**Reply:** The word was changed to “used”, please check line 105. We have also checked the manuscript to correct similar problems.

5. Please use simple and easily understandable English. Please do not use words like 'adhibition' line no. 134. kindly rephrase this sentence and correct others throughout the manuscript.

**Reply:** The word was changed to “development”, please check line 146. We have also checked the manuscript for similar problems.

6. Kindly correct grammar throughout the manuscript. For example, refer line no. 390, remove 'the' in "On the one hand...". The manuscript appears to be written in short-cut language and is devoid of proper sentence construction at a number of instances.

**Reply:** We have deleted these words, please check line 337. We have also done the proofread to avoid similar problems.

7. Table 5 appears to be too long.

**Reply:** To shorten the tables, we have used abbreviations and reduced the size of the words. Please check the table at line 391.

8. Conclusion cannot be two pages long. Kindly condense it in 3-4 bulleted sentences.

**Reply:** We have rewritten the conclusion by condensing it. Please check the conclusion from lines 606 to 621.

9. Enclosed figures are neither legible nor readable. Please attach them in clear picture format.

**Reply:** We have adjusted and reuploaded the figures, please check them.

Please let us know if you have any questions or further feedback.

I hope our revisions can satisfy your comments and questions.

Sincerely yours,

Dong and Itoh
